# Functional Representation of Enzymes by Specific Peptides

**DOI:** 10.1371/journal.pcbi.0030167

**Published:** 2007-08-24

**Authors:** Vered Kunik, Yasmine Meroz, Zach Solan, Ben Sandbank, Uri Weingart, Eytan Ruppin, David Horn

**Affiliations:** 1 School of Computer Science, Tel Aviv University, Tel Aviv, Israel; 2 School of Physics and Astronomy, Tel Aviv University, Tel Aviv, Israel; 3 Sackler School of Medicine, Tel Aviv University, Tel Aviv, Israel; Columbia University, United States of America

## Abstract

Predicting the function of a protein from its sequence is a long-standing goal of bioinformatic research. While sequence similarity is the most popular tool used for this purpose, sequence motifs may also subserve this goal. Here we develop a motif-based method consisting of applying an unsupervised motif extraction algorithm (MEX) to all enzyme sequences, and filtering the results by the four-level classification hierarchy of the Enzyme Commission (EC). The resulting motifs serve as specific peptides (SPs), appearing on single branches of the EC. In contrast to previous motif-based methods, the new method does not require any preprocessing by multiple sequence alignment, nor does it rely on over-representation of motifs within EC branches. The SPs obtained comprise on average 8.4 ± 4.5 amino acids, and specify the functions of 93% of all enzymes, which is much higher than the coverage of 63% provided by ProSite motifs. The SP classification thus compares favorably with previous function annotation methods and successfully demonstrates an added value in extreme cases where sequence similarity fails. Interestingly, SPs cover most of the annotated active and binding site amino acids, and occur in active-site neighboring 3-D pockets in a highly statistically significant manner. The latter are assumed to have strong biological relevance to the activity of the enzyme. Further filtering of SPs by biological functional annotations results in reduced small subsets of SPs that possess very large enzyme coverage. Overall, SPs both form a very useful tool for enzyme functional classification and bear responsibility for the catalytic biological function carried out by enzymes.

## Author Summary

Sequence motifs are known to provide information about functional properties of proteins. In the past, many approaches have looked for deterministic motifs in protein sequences, by searching for functionally over-represented k-mers, with moderate levels of success. Here we revisit and renew the utility of deterministic motifs, by searching for them in a partially unsupervised and context-dependent manner. Using a novel motif extraction algorithm, MEX, deterministic sequence motifs are extracted from Swiss Prot data containing more than 50,000 enzymes. They are then filtered by the Enzyme Commission classification hierarchy to produce sets of specific peptides (SPs). The latter specify enzyme function for 93% of the data, comparing well with existing approaches for enzyme classification. Importantly, SPs are found to have biological significance. A majority of all known active and binding sites of enzymes are covered by SPs, and many SPs are found to lie within spatial pockets in the neighborhood of the active sites. Both these results have extremely high statistical significance. A user-friendly tool that displays the hits of SPs for any protein sequence that is presented as a query, together with the EC assignments due to these SPs, is available at http://adios.tau.ac.il/SPSearch.

## Introduction

One of the major efforts of computational research in molecular biology is to predict the function and spatial structure of proteins from the protein sequence of amino acids [1,2]. Conventional approaches to function prediction rely on sequence [3] or structure [4] similarity with proteins whose functions are known. This is sometimes misleading [4–6]. Alternatively, one may use motif approaches [7–12], trying to extract from the data subsequences that are responsible for particular functions. Motifs can be deterministic sequences of amino acids, regular expressions that allow various alternatives for specific locations within the motif, or stochastic structures specifying the probability of an amino acid at every location. This work aims to uncover deterministic sequence motifs, and considers their relationships with protein functionality. We focus on enzymes, whose functions are classified by the Enzyme Commission (EC) four-level hierarchy which is represented by four integers, n1.n2.n3.n4, corresponding to the different levels of classification. For example, the oxidoreductases class corresponds to n1 = 1, one of the six main divisions. For this class, n2 (subclass) specifies electron donors, n3 (sub-subclass) specifies electron acceptor, and n4 indicates the exact enzymatic activity.

Conventional sequence motif searches in enzymes are performed in a supervised fashion, using sequences of proteins that are known to have the same function and looking for (deterministic, regular-expression, or stochastic) motifs that are over-represented in this group of proteins. The motifs in question should then subserve such functions as [9] phosphorylation of protein kinases; metal binding sites for calcium, zinc, copper, and iron; enzyme active sites, etc. With the advent of studies of protein–protein interactions, interest grew in finding sequence motifs that are responsible for them, and span an “interaction space” [13,14].

**Figure 1:**
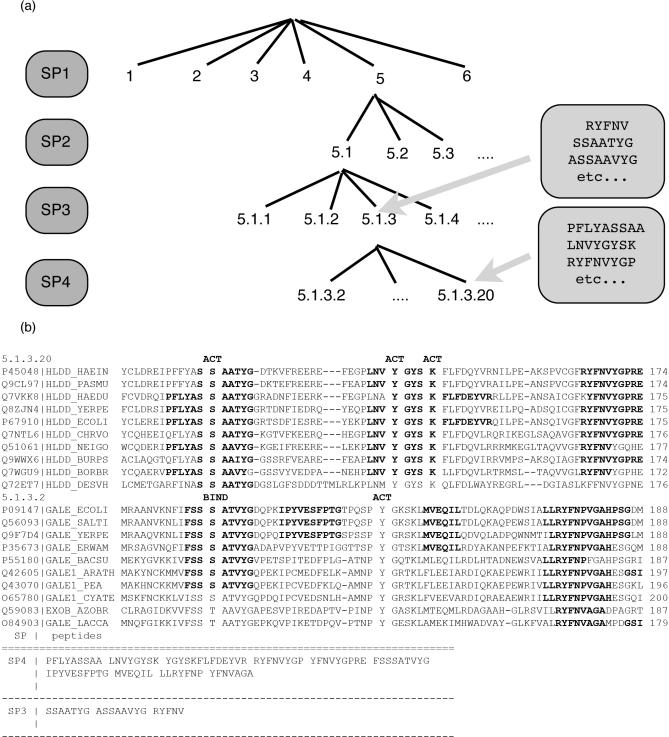
The Occurrence of Specific Peptides within the EC Hierarchy of Enzymes (A) A sketch of the EC hierarchy and the assignments of SPs to SP classes. SPs can be compared with those appearing in Figure 1B. (B) Aligned sequences of two groups of enzymes of level 4 that share the same third-level assignment. Alignment is performed according to SPs. The organisms in the upper group, 5.1.3.20, belong to proteobacteria, while those of the lower group, 5.1.3.2, also contain eukaryotes (ARATH, CYATE, and PEA). Boldfaced substrings denote SPs. Amino acids flanked by spaces denote active sites and binding sites, as indicated above. A list of all SPs and their assignments to SPN classes is presented below the sequences.

Here we perform a large-scale search for deterministic sequence motifs without specifying a priori their exact functional roles, using the unsupervised motif extraction (MEX) algorithm [15]. We have used one functional guidance: MEX was separately applied to each one of the six major EC classes. The same motifs may also appear in other classes, yet many of them turn out to occur in only one class, and belong to a specific EC branch. The latter (see Figure 1A) are termed specific peptides (SPs). By representing some 50,000 enzymes (of average length of 380 amino acids) in terms of about the same number of SPs (of average length 8.4), we obtain a largely compressed functional representation and an EC classification with 93% accuracy.

This may be compared with other methods based on e-motifs [16], sequence similarity [17], or physicochemical properties of the amino acids contained in the sequence [18,19]. Our results compare favorably with such methods, as will be shown below, yet our approach differs in several respects: we use a largely unsupervised motif extraction method, we perform a comprehensive study of all enzymes, and we put major emphasis on the biological relevance of the SPs themselves.

Importantly, in comparison with the large-scale and popular motif database ProSite [8], our approach displays a wide-margin advantage, their motifs coverage extending only to 63% of all enzymes in the database.

## Results

### The Specific Peptides

SPs, as defined above, are MEX motifs that are specific to a single branch of the EC hierarchical classification. Most belong to single branches of the fourth level of the hierarchy, to be denoted as SPs of level 4 (SP4) (see Figure 1A). SPs of higher hierarchy, SP3, SP2, and SP1, appear in more than one lower EC level. Thus, if a peptide is shared by two or more level 4 groups that belong to the same third EC level, and appears nowhere else, it is assigned to SP3. The SPs were further screened to eliminate any peptide that includes within it another peptide carrying the same SPN (N = 1,2,3,4) label.

The majority of SPs found at level 4 of the EC hierarchy (Table 1) are probably due to the high homology within this level, that often includes many orthologous genes. Thousands of SPs occur at higher levels of hierarchy, reflecting functional similarity among enzymes with lower sequence similarity. The occurrence of any one SP on the sequence of an enzyme specifies its EC functionality according to the specific branch N of its SPN. For example, enzyme P45048 (see Figure 1B) contains SSAATYG, an SP3 specific to 5.1.3, and LNVYGYSK, an SP4 specific to 5.1.3.20. The relationship of these SPs to the EC hierarchy of SP families is shown in Figure 1A.

**Table 1:**
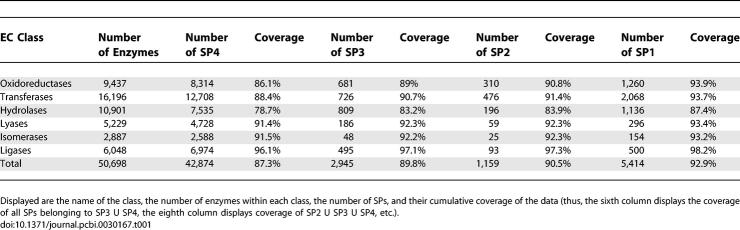
Specific Peptides in All Six Classes of Swiss-Prot Release 48.3

Table 1 shows that the SPs cover (i.e., appear on the sequence of) most enzymes in the dataset. The coverage columns display the cumulative coverage of all SPs to their left. Coverage is a measure of the success of the SP approach. Thus, from the sixth column one can deduce that functional classification at the third level of EC is specified by 45,819 peptides of SP3 and SP4, covering 89.8% of the data.

Information about the separate coverage of each SPN group is provided in Table S1. The length distribution of SPs is displayed in Figure S1 for all enzyme classes. No SP exists with a length shorter than four amino acids. The average SP length is 8.4 (s.d. 4.5). The distribution of the number of SPs occurring on enzymes is given in Figure S2. It is very flat. On average, 15.6 SPs appear on each enzyme and the standard deviation is 16. Enzyme sequences that share long SPs are highly similar, while sharing short SPs indicates smaller sequence similarity. This is displayed for short (smaller than nine amino acids) and medium length (between nine and 12 amino acids) SPs in Figures S3 and S4: most enzyme pairs that share SPs of length larger than 12 amino acids possess sequence identity of over 90%.

### Prediction of Enzyme Classes

The SwissProt 48.3 dataset contains 260 enzymes that have more than one annotation, and, therefore, have been excluded from the training set (see Methods). Using them as a test set, we find 849 hits of SPs on 157 of these enzymes. 711 of the 849 hits agree with one of the given annotations and 138 do not, thus obtaining an accuracy of 84%. The results are displayed in Table S2, comparing the Swiss-Prot EC annotations with SP predictions. For example, the first protein on the list has Swiss-Prot EC annotations of 2.7.2.4 and 1.1.1.3. Its sequence matches two SPs, one SP1 of class 1 and one SP4 of 2.7.2.4. This is counted as two correct matches. An analysis of Table S2 shows that predictions based on a single SP hit may be erroneous, while those based on more than two SPs whose EC assignments are consistent with one another are correct.

We have tested the generalization quality of our SP-based enzyme classification by running MEX on the Swiss-Prot 45 release (October 2004) and testing its predictions on 10,000 novel enzymes that are listed in the Swiss-Prot 48.3 release (for the relation between these two sets see Figure S5 and Table S3). Generalization quality is assessed in Table 2 by recall (matching SPs extracted from the 45 data on novel enzymes) and precision (correctness of the “45” EC assignment according to “48.3” annotations). Precision can be defined at the SP level, i.e., to what extent did the EC of this SP match the true EC of the enzyme that it hits. Precision can also be defined at the enzyme level: how many enzymes are correctly identified by all SPs that hit them. In other words, demanding the EC assignments of all SPs to be consistent with one another as well as with the “48.3” annotation of the enzyme. Overall recall is 84%. Precision at the SP level is almost perfect, 98.7%; nonetheless, at the enzyme level it reduces to 81.7%. The reason is that usually there are many SPs hitting each enzyme, and the small error at the SP level is magnified by the requirement that the EC labels of all SPs on the same enzyme should be consistent with each other.

**Table 2:**
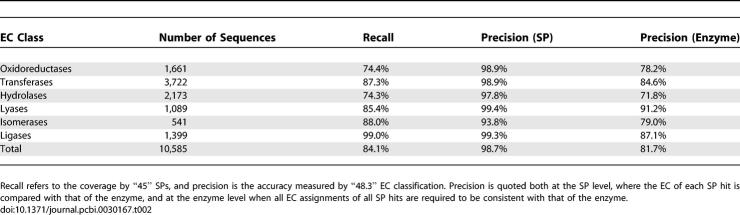
Performance of SPs Extracted from the Swiss-Prot 45 Dataset on Novel Enzyme Sequences in Swiss-Prot 48.3

This generalization test suffers from bias, i.e., there exist enzymes in the test set that have high sequence similarity to some enzymes in the training sets. In conventional machine-learning analysis of sequence to function classification [2], one often tries to eliminate bias by avoiding high sequence similarity between proteins in the test set and proteins in the training set. In our case this is problematic, because it effectively calls for eliminating from the test set all enzymes that have four-digit EC numbers appearing in the training set. Alternatively, one could produce for each enzyme in the test set a new training set that does not contain sequences with the same EC number, which is both unconventional and computationally very complex.

To overcome this predicament, we have used the following procedure: a) start with the test set consisting of all sequences of SwissProt release 48.3 that do not appear in release 45; b) blast each one of these (test set) sequences against the sequences of the training set (SwissProt release 45) that do not have the same four-digit EC number; c) include in the non-redundant test set only sequences whose BLAST score [20] with all other training sequences (including those with the same first three EC digits) is larger than 10^−3^; d) test generalization on this non-redundant set only for peptides in SP1, SP2, and SP3, thus avoiding the SP4 peptides that were extracted from the same fourth-level EC sequences as those of the non-redundant test set. It should be noted that removing the SP4 peptides makes the functional annotation task much more difficult because the coverage of enzymes by SPs is strongly reduced. Only 440 enzymes obey the BLAST > 10^−3^ condition, and less than 40% of them carry SP1, SP2, and SP3 matches.

The results are displayed in Table 3. We obtain correct classification with an accuracy of 88%. The test is that of precision of SP assignments, i.e., to what extent do the EC labels of the SPs, observed to exist on the enzyme sequences, correspond to “48.3” EC classifications.

**Table 3:**
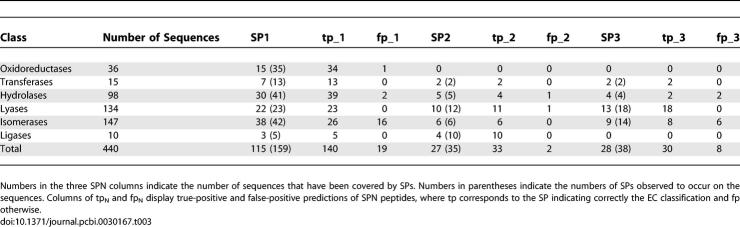
Coverage of a Non-Redundant Test Set by Motifs in SP1, SP2, and SP3

Whereas even the unbiased tests have high precision, we should emphasize that many successes of the SP approach are due to SP4 peptides, whose existence stems from high homology among different sequences that belong to the same EC number. These successes include the high coverage of enzymes (see Table 1) and the coverage of active and binding sites to be discussed below. The fact that these SPs have been extracted by MEX may be viewed as the essence of homology, as illustrated in Figure 1B, where the existence of SPs is displayed on various enzymes aligned according to their matching SPs.

We provide a Web tool, available at http://adios.tau.ac.il/SPMatch, which displays the hits of SPs for any protein sequence that is presented as a query, together with the EC assignments due to these SPs.

### Comparison with Other Methods

We have tested the usefulness of the SP approach by comparing it with conventional functional prediction methods. For this purpose we have used all oxidoreductases in the 48.3 data and divided them into training data and test data with a 75%:25% ratio. MEX was run on all data and SPs were selected from the MEX motifs according to the training data. Only this subset of motifs was then employed to classify the test data. This procedure has been repeated 45 times to gain statistics, and has been subjected to a support vector machine (SVM) analysis. It has been compared with a state-of-the-art method [17] based on an analogous SVM procedure, applied to the same data using the same divisions and relying on classification of (train and test) data according to a matrix of Smith-Waterman distances from all oxidoreductases. The results are displayed in Tables S4 and S5 and show a clear advantage to SP classification. For comparison, we use the Jaccard score defined as J = TP / (TP + FP + FN) where TP, FP, and FN denote true positives, false positives, and false negatives, accordingly. Whereas sequence similarity leads to an average Jaccard score of 0.86 on the second EC level and 0.82 on the third level, SP classification has average Jaccard scores of 0.93 and 0.92, accordingly. Comparing with yet another method, SVM-Prot [18,19], which classifies enzymes on the basis of physical and chemical features of their amino acids, we note that the latter achieves a Jaccard score of only 0.74 on all oxidoreductases data at the second EC level.

The common lore, that large sequence identity between two proteins implies that the two have the same function, has its exceptions. Motifs, although often extracted from homology, may serve as better measures for functional specification of proteins [21] than overall sequence similarity. Table 4 demonstrates this point, by contrasting SP predictions with Smith-Waterman similarity results for pairs of enzymes. These extreme cases have been posed as a problem by Ross [5] (see Table 1 there). All displayed EC assignments correspond to those of SPs located on the enzyme sequences, and match the correct EC numbers. As a more detailed example, we point out that the enzymes of the sixth pair in Table 4, GTFB_STRMU and AMY3B_ORYSA, have 42% sequence identity along an alignment of 105 amino acids. Nonetheless, the sequences are not identical at the SP locations. AMY3B_ORYSA contains 24 SPs, none of which have an exact match on GTFB_STRMU, and a single SP4 (GGAFLE) found on the latter matches correctly its EC number.

**Table 4:**
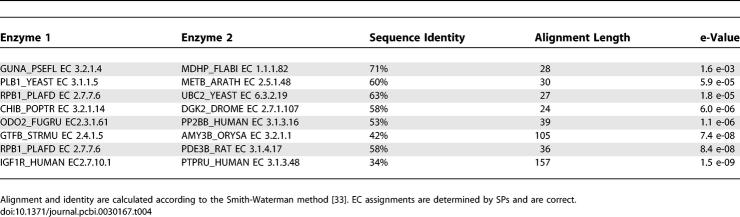
Enzymes with High Sequence Similarity and Different EC Assignments

It is of interest to compare our SPs with ProSite motifs [8], which are listed in the Swiss-Prot database as standard motif annotations on 63% of the enzymes. ProSite motifs are either regular expressions (of average length 18.3 amino acids) or weight matrices, while SPs are deterministic motifs (with average length of 8.4). We search for all appearances of ProSite regular expression motifs on enzymes. Each such appearance is noted on the enzyme sequence and checked whether it is also (partially) covered by an SP. Figure S6 compares the appearance of SPs and ProSite motifs on the data, and Figure S7 displays the relative coverage of ProSite motifs by SPs as function of the minimal percentage of amino acids belonging to the ProSite motif that are also located on SPs. Thus we find that if at least 40% of the amino acids of the ProSite motif also belong to SPs, which would be appropriate for an average SP to be located within an average ProSite motif, then SPs cover 48% of all ProSite motif occurrences. This may be compared with a random model (see Methods) which covers on average only 24% of ProSite motif occurrences, with a standard deviation of 0.06%. This extremely significant result (400 s.d.) demonstrates that SPs carry information that is highly correlated with that of ProSite motifs.

### Biological Roles of Specific Peptides

#### Coverage of active sites.

Next we turn to establishing some particular biological roles for SPs. First we investigate their coverage of active and binding sites. 42% of all enzymes in the Swiss Prot 48.3 database have annotations of loci of active sites and binding sites (single amino acids). For simplicity we will refer to both annotations as active sites. A few examples are shown in Figure 1B. Given these loci, we find that 65% of all active sites are covered by SPs. This can be compared with the coverage of random positions on the same enzyme sequences which, on average, is only 27% (off by 80 standard deviations, see Methods). We also construct a non-redundant set by choosing only one enzyme for each EC number (i.e., EC class of level 4). The results, displayed in Table 5, show some differences between the total and the non-redundant sets. Since the latter is unbiased, it should generalize better, and allow us to get a better estimate of active-site coverage had the annotations existed for all enzymes. This estimate is 12% and has very high statistical significance (zero *p*-value, see Methods).

**Table 5:**
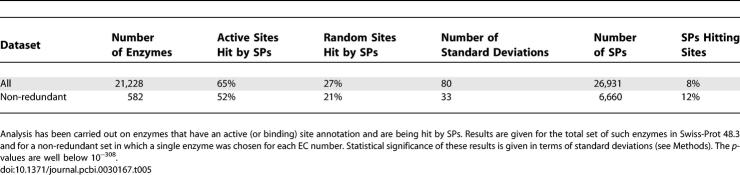
Occurrence of Specific Peptides on Active Sites

As an example of these features in the data, we display in Figure 1B aligned subsequences of enzymes, belonging to the same third level but to two different fourth levels of the EC hierarchy: six out of 35 enzymes of 5.1.3.2 and seven out of 29 enzymes of 5.1.3.20. Shown are strings belonging to the sequences that include active sites and binding sites as indicated in Swiss-Prot annotations, and boldfaced substrings denoting SPs from our lists. Whereas in 5.1.3.20, most active sites are covered by SPs, this is not the case for the active site of 5.1.3.2. Nonetheless, it turns out from investigating spatial structures of these enzymes that RYFNV, an SP that appears in both groups, is located within the same pocket in which the active site resides. This may be regarded as an indication that RYFNV plays an important role in fostering the biological function of this enzyme.

**Figure 2:**
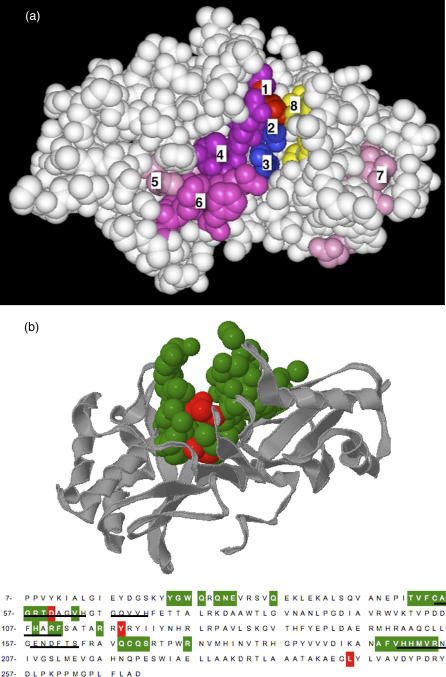
SPs Occurrence on a Spatial Structure of an Enzyme (A) 3-D display of enzyme P07649 (PDB code 1DJ0), belonging to 5.4.99.12, showing (1) an active site D at sequence location 60; (2) a binding site Y at location 118; (3) a binding site L at location 245. The active site is common to two SPs (4) containing (CAGRT(D)AGVH). Other shown SPs are (5) GQVVH at locations 67–71; (6) FHARF at 107–111, known to be a tentative RNA-binding peptide; (7) ENDFTS at 157–163; and (8) HMVRNI at 201–207, sharing a pocket with the active and binding sites. QVVH and ENDFTS belong to SP3, all other peptides belong to SP4. (B) A different display of the same enzyme focuses on the pocket containing the active site. The relevant section of the sequence is shown, with red residues signifying active and binding sites, green residues corresponding to other amino acids residing in the pocket, and underlined residues corresponding to SPs.

An example stressing the relationships among SPs and spatial structures is presented in Figure 2. This enzyme contains many SPs. Two SPs cover the active site, one—HMVRNI—shares a pocket with the active site and the two binding sites, and another one—FHARFV—plays the role of RNA binding in this tRNA pseudouridine synthase I.

FHARF is one example of previously discovered motifs [22]. Some other examples are: a) GFGRIG (SP of 1.1.1.26) [23], a conserved region of GAPDH that is active in the glycolytic pathway; b) HRDLKP (SP of 2.7.1.37) [24], appearing in protein kinases; c) IFIDEID (SP of 3.6.4.3), the Walker B motif of ATPase [25]; to name a few. However, most of the SPs have not been studied before.

These results raise the question how many SPs can be found in the neighborhood of active sites, as defined by the pockets in the spatial structures of enzymes. One is naturally tempted to assign importance to all SPs of this kind, not just those that carry the active site annotation (single amino acid). For this study we use the CASTp [26] database, which lists all amino acids belonging to pockets appearing in spatial structures of proteins. We select 1,031 enzymes that possess pockets including active (or binding) site annotations. There are 8,860 SPs that occur on these enzymes, 31% of which lie within these “active pockets,” i.e., have at least four amino acids that reside in the pocket. Defining a background model (see Methods) of random peptides selected for each event of an SP hitting an active pocket in a particular enzyme, we estimate that 11% of all SPs belong to events that pass an FDR limit [27] of 0.05. Most of them (70%) do not contain an active site; hence, they are of potential interest for experimental verification of their importance in defining and maintaining the enzymatic function. Table 6 summarizes the results of this analysis. Further details of all significant events are presented in Table S6. All 1,910 listed SP occurrences on enzymes should be of high relevance to the biological functions of these enzymes, and the elimination of any one of them from the enzyme sequence on which it occurs should be deleterious to the function of that enzyme or to its stability.

**Table 6:**
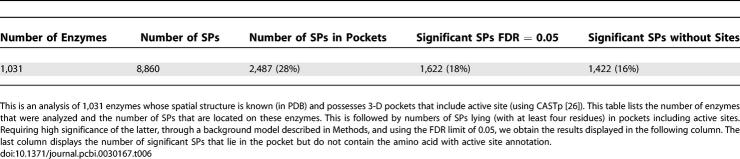
Occurrence of SPs in Spatial Proximity to Active Sites

SPs may also have biological roles that are not connected to active or binding sites. Examples are DNA and RNA binding, metal binding, protein–protein interactions, etc. Given the large number of SPs, we may look forward to a plethora of predictions.

### Minimal SP Sets with Maximal Coverage

We started our study with 50,698 enzymes from which 52,365 SPs were extracted. These SPs provided coverage of about 93% of all enzymes. By introducing further screening of SPs according to biological findings, a much reduced number of SPs may suffice for the purpose of classification. 21,228 enzymes carry active or binding site annotations in the 48.3 data. The number of SPs hitting these enzymes is 26,931; however, only 2,337 cover the active or binding sites. These 2,337 are found to occur on 79% of the 21,228 enzymes. Thus, instead of the approximately 1:1 ratio between the number of SPs and the number of enzymes they cover as found previously, we now obtain an order of magnitude parsimonious ratio, of about 1:8, while maintaining a similar level of classification accuracy.

The same SPs cover 36% of all original enzymes of our dataset. Performing a similar analysis on the 45 data, one finds that the 2,014 SPs that cover the annotated enzymes in it hit 75% of the relevant set of enzymes. Moreover, using the same SPs to classify the 10,585 novel enzymes contained in the 48.3 release and absent from the 45 release, one obtains coverage of 28% of them. This last fact demonstrates that the relatively large coverage reached by the small fraction of SPs that hit active sites is not limited to the dataset (training set) used to define the SPs. All these results are summarized in Table 7. It seems therefore quite reasonable to conclude that, adding information of biological markers, one can reduce the ratio of the number of SPs deduced from a certain number of enzymes and needed to label their EC classification from 1:1 to about 1:8.

**Table 7:**
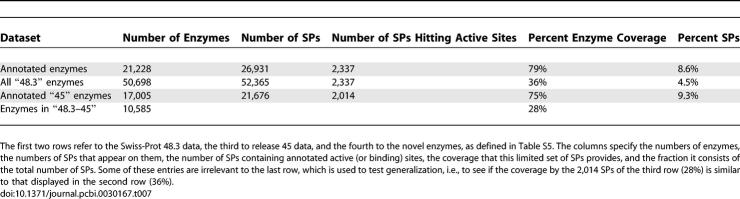
Small Sets of SPs that Contain Active Sites Suffice To Specify Functionality of Many Enzymes

This, however, does not mean that all other SPs should be disregarded. First, there exist good chances that they are of biological importance for various structural and functional reasons that may warrant further investigation. Second, when extreme classification issues come up, as in the cases displayed in Table 4, every single SP may count.

## Discussion

Conventional wisdom attributes protein functions to large domains, as well as to specific amino acids at strategic structural points on the protein. Large-scale studies often make use of multiple sequence alignment (MSA), phylogenetic information, and sophisticated mathematical models, thus leading to the plethora of algorithms and Web tools that permeate bioinformatics. While all that may be necessary to obtain a thorough understanding of the way proteins develop and perform, much can be gained by shifting attention to deterministic linear motifs on proteins. In doing so, we return to a way that has been often tried in the past. Thus, in the 1990s, many investigations looked for k-mers that are over-represented in sequences of proteins that have common functional properties. Some examples are ProSite [8,12], with which we have compared our results, and papers such as [28–30], where major emphasis has been put on finding a complete dictionary of motifs that cover all strings of amino acids that are of any importance. In the case of [30], the search has been an unsupervised one leading eventually to a coverage of 98% of all amino acids on the protein strings. Some reviews of the motif approaches of the 1990s are [7,9]. More recently, interests have shifted to automated prediction tools that may make use of motifs but are not limited to them. Examples are the GOtcha method [31] that uses sequence-identity searches of various genomes to predict functional annotation, and [32] who pursue the same goal using PSI-BLAST searches with varying resolution.

Our goal is more moderate, restricting ourselves to the functional classification of enzymes. By doing so, and by applying the MEX algorithm together with limiting ourselves to SPs within the EC hierarchy, we are able to classify all enzymes by SPs occurring on them with coverage between 87% to 93%, depending on the EC level that is being looked for (Table 1). Classification success of novel sequences that belong to the same type of data has coverage of 84% and precision of 99% at the SP level and 82% at the enzyme level (Table 2). Restricting ourselves to low bias (Table 3), we still have a large precision of 88% at the SP level. We have demonstrated that our results surpass the classification accuracy of sequence similarity (using Smith-Waterman [33]), and our SPs have a higher coverage than ProSite motifs. As such, they become a powerful tool that may be added to existing automated searches.

It should be noted that the SPs were extracted by an unsupervised motif search algorithm, applied to each one of the six EC classes. This is quite different from conventional supervised approaches. Our method may disregard motifs that obey some over-representation criterion, and choose others that do not satisfy such a global statistics measure. Another major difference from other approaches is that we do not make use of MSA. MEX finds significant motifs without requiring alignment as a preprocessing stage. In fact, MEX can serve as a source for MSA by employing its motifs for alignment (see Figure 1B).

SPs were selected from all MEX motifs by imposing the condition that they should be specific to particular levels of the EC hierarchy. This has led to a large number of SPs, as numerous as the set of all enzymes (but, obviously, providing a much more concise description). Imposing further biological conditions, one may find much smaller sets that suffice for classification. In an analysis of enzymes for which the active sites are known, we have shown that the set of SPs bearing these active sites, which comprises just 8.6% of all relevant SPs (i.e., those occurring anywhere on these enzymes), suffices to cover (and therefore label) all enzymes.

Conventional classification methods rely on homology. While large homology is also at the root of our success for most SPs of level 4 (see some examples in Figure 1B), we have demonstrated (in Table 4) that SPs can also be of importance in extreme cases, where straightforward comparison of an enzyme to another one with large sequence similarity may be misleading.

In conclusion, we have established a comprehensive and accurate classification scheme for enzymes based on the occurrence of short peptides on their sequences. The SPs contain, on average, just 8.4 amino acids, yet they suffice to correctly classify an overwhelming majority of known enzymes. Moreover, we have found indications for some of the biological roles of SPs, e.g., covering a majority of active sites. This study has laid the foundations for the further experimental investigation of these intriguing sets of SPs.

## Methods

### Motif extraction.

MEX is a motif extraction algorithm that serves as the basic unit of ADIOS [15], an unsupervised method for extraction of syntax from linguistic corpora. We apply it to the problem of finding sequence motifs in enzymes.

Each enzyme sequence is represented as a path over a graph containing 20 vertices, each vertex representing one amino acid. After uploading all enzyme sequences onto the graph, one counts the number of paths connecting vertices in order to define probabilities such as

p(e_j_|e_i_) = (number of paths proceeding from e_i_ to e_j_) / (total number of paths leaving e_i_)

p(e_k_|e_j_,e_i_) = ( number of paths proceeding from e_i_ to e_j_ to e_k_) / (number of paths proceeding from e_i_ to e_j_)

for all vertices e_i_ of the graph. These data-driven probabilities allow for the definition of a position-dependent variable-order Markov model describing the data.

A motif that is extracted by MEX is a subpath along the graph defined by probability-based criteria that account for convergence of many paths into the beginning point of a motif, and divergence of many paths from the endpoint of the motif. Motifs are not constrained by length, and may overlap with one another (see, e.g., the two SPs that overlap at the active site D in Figure 2B). The only two parameters of MEX are η, specifying a decrease in probability measures that determine convergence and divergence, and α specifying their statistical significance. For more details, see [15] and http://adios.tau.ac.il. Throughout this paper, we use η = 0.9 and α = 0.01.

### Data.

Protein sequences annotated with EC numbers were extracted from the Swiss-Prot database (Release 48.3, 25 October 2005). To obtain a high-quality, well-defined training dataset, the data were strictly screened and the following sequences were removed: sequences shorter than 100 amino acids or longer than 1,200 amino acids, sequences with uncertain annotation, and enzymes that catalyze more than one reaction (e.g., have more than one EC number).

### Random model for SP hits on ProSite motifs.

Enzyme sequences are searched for matches with regular expressions of ProSite motifs. The resulting strings of amino acids are checked for matches with SPs. The latter are compared with matches of a random model where, for each given enzyme, random peptides are selected with the same lengths as those of the SPs that hit this enzyme. The random model provides a probability distribution which serves as a zero model for calculating the significance of the SP hit on the ProSite motif. This comparison is being made for each enzyme and for varying fractions of amino acids that are shared by the SP with the ProSite motif.

### Significance of SP hits on active sites.

In analyzing the significance of SP coverage of active (and binding) sites, we compare this coverage with that of randomly chosen residues on enzyme sequences. This is carried out on all data (i.e., annotated enzymes with SP hits) and on a non-redundant set composed of only one enzyme from each EC number (i.e., EC classification at level 4). The deviations of the measurements from random distributions are very high, and are quoted in numbers of standard deviations. The corresponding *p*-values are zero according to Matlab accuracy, i.e., are well bellow 10^−308^.

### Significance of SP residing in active pockets.

Let us define an event as the occurrence of a given SP within an active pocket in a given enzyme. For each such event, we evaluate the probability that at least one of randomly selected sequences from this enzyme, which coincide in length with the various SPs that occur on this enzyme, lies (with at least four amino acids) within the active pocket. This defines the *p*-value that we assign to the event. We then select the significant events according to an FDR limit [33] of 0.05.

## Supporting Information

Figure S1 SP Length Distribution (377 KB JPG)

Figure S2 Distribution of the Numbers of SPs Occurring on Enzymes (500 KB JPG)

Figure S3 Distribution of Percentages of Sequence Identity for Pairs of Enzymes Sharing the Same SP3 or SP4 of Length Less Than Nine Amino Acids (172 KB JPG)

Figure S4 Distribution of Percentages of Sequence Identity for Sets of Enzymes That Share the Same SP3 or SP4 of Length between 9 and 12 (156 KB JPG)

Figure S5 Relation of Enzymes in Two Swiss-Prot Releases, 45 (October 2004) and 48.3 (October 2005) (118 KB JPG)

Figure S6 Data Coverage by ProSite Regular Expression Motifs and by SPs in the Swiss-Prot Database (183 KB JPG)

Figure S7 Coverage of ProSite Motifs by SPs versus the Required Minimal Amount of Amino Acids Shared by the Two Motifs For each ProSite motif (of average length 18 amino acids) occurrence on an annotated enzyme, SP matches were searched. The cumulative percentage of ProSite motifs that are covered by SPs is plotted as a function of the relative amount of coverage, i.e., the percent of the number of amino acids belonging to the ProSite motif that is shared by the SP. This is compared with the coverage of ProSite motifs by random motifs that have the same length and number as the SPs appearing on the enzymes
(360 KB JPG)

Table S1 Coverage by SPs of Enzymes in Swiss-Prot Release 48.3 (30 KB DOC)

Table S2 Comparison between Swiss-Prot Annotations and SP Predictions for Doubly Annotated Enzymes Columns indicate the protein ID according to Swiss-Prot, its two EC assignments, the EC assignments according to SP predictions, and the number of SP matches that have the same EC prediction (separated into correct and false predictions). An analysis of the data shows that predictions that are based on a single SP match in the enzyme sequence are often wrong (122 false predictions versus 80 true predictions). The appearance of two SPs whose EC assignments are consistent with each other leads to 19 true predictions and five false predictions. All predictions based on more than two consistent SPs are true. When counting enzymes (rather than SPs), we find that 92 of 157 had one false prediction and no true prediction. 48 enzymes have one false prediction; 31 of them have also one true prediction, and 17 have two true predictions. 65 enzymes have no false prediction; 43 of them have one true prediction and 22 have two true predictions. It should be noted that this list of enzymes contains many related enzymes (i.e., it has high bias), hence successes and failures in different enzymes are correlated. It seems safe, however, to conclude that predictions based on several SPs whose EC assignments are consistent with each other may be trusted.
(808 KB DOC)

Table S3 Numbers of Enzymes in Swiss-Prot Release 48.3 and Swiss-Prot Release 45 (34 KB DOC)

Table S4 Comparison of SP with Smith-Waterman Performance on Classification at the Subclass Level Classification based on SPs has been compared with classification based on sequence similarity using the Smith-Waterman (SW) method. This has been performed on the oxidoreductases data of the 48.3 release, using all subclasses and sub-subclasses that contain more than 20 enzyme sequences. The data were randomly partitioned into 75% training and 25% test sets. Features for the SP classification were determined by running MEX on all oxidoreductases and checking for their specificity using the training data only. These SPs were then used for defining, through training, the SP-SVM. Smith-Waterman analysis was carried out by defining a log(*p*-value) (with cutoff at p = e−06) distance matrix whose columns (features) were all oxidoreductases. The rows (instances) of the training-set enzymes were used to determine the SW-SVM classifications. 45 different partitions were performed to accumulate statistics. Same partitions were applied to both classification methods. Classification was performed using a soft-margin linear SVM, available online at http://svmlight.joachims.org.
Performance was measured by the Jaccard score J = TP / (TP + FP + FN).
(58 KB DOC)

Table S5 Comparison of SP with Smith-Waterman Performance on Classification at the Sub-Subclass Level (99 KB DOC)

Table S6 List of SPs That Lie in Active Pockets A list of all events of SPs lying in active pockets that have passed the FDR = 0.05 limit, ordered according to their *p*-values. Entries include the enzyme PDB ID and the details of the SP.
(2.1 MB DOC)

## Abbreviations

EC: Enzyme Commission
MSA: multiple sequence alignment
SP: specific peptides
SVM: support vector machine
