# Supplementary material for: Functional Representation of Enzymes by Specific Peptides
Source: PLoS Comput Biol. 2007 Aug 24;3(8):e167. doi: 10.1371/journal.pcbi.0030167 (PMC1950953; doi:10.1371/journal.pcbi.0030167)
Supplement: Supplementary file 1 [file pcbi.0030167.st001.doc]

**Table S1: Coverage by SPs of enzymes in Swiss-Prot release 48.3.**

| **EC class** | **SP4** | **SP3** | **SP2** | **SP1** |
| --- | --- | --- | --- | --- |
| Oxidoreductases | 86.1% | 27.6% | 18% | 75% |
| Transferases | 88.4% | 33.7% | 27.4% | 70% |
| Hydrolases | 78.7% | 27.7% | 19% | 57.8% |
| Lyases | 91.4% | 29.7% | 15.5% | 48.2% |
| Isomerases | 91.5% | 16.8% | 9.7% | 39.9% |
| Ligases | 96.1% | 55% | 18.2% | 64.1% |
| total | 87.3% | 32.47% | 20.52% | 63.8% |
