# Supplementary material for: Functional Representation of Enzymes by Specific Peptides
Source: PLoS Comput Biol. 2007 Aug 24;3(8):e167. doi: 10.1371/journal.pcbi.0030167 (PMC1950953; doi:10.1371/journal.pcbi.0030167)
Supplement: Supplementary file 2 [file pcbi.0030167.st002.doc]

**Table S2: Comparison between Swiss-Prot annotations and SP predictions for doubly annotated enzymes.** Columns indicate the protein id according to Swiss-Prot, its two EC assignments, the EC assignments according to SP predictions, and the number of SP matches that have the same EC prediction (separated into correct and false predictions).

An analysis of the data shows that predictions that are based on a single SP match in the enzyme sequence are often wrong (122 false predictions *vs* 80 true predictions). The appearance of two SPs whose EC assignments are consistent with each other leads to 19 true predictions and 5 false predictions. All predictions based on more than 2 consistent SPs are true. When counting enzymes (rather than SPs) we find that 92 of 157 had one false prediction and no true prediction. 48 enzymes have 1 false prediction; 31 of them have also 1 true prediction and 17 have 2 true predictions. 65 enzymes have no false prediction; 43 of them have 1 true prediction and 22 have 2 true predictions. It should be noted that this list of enzymes contains many related enzymes (i.e. it has high bias), hence successes and failures in different enzymes are correlated. It seems however safe to conclude that predictions based on several SPs whose EC assignments are consistent with each other may be trusted.

| **ID** | **EC1** | **EC2** | **SP Prediction** | **# correct matches** | **# false matches** |
| --- | --- | --- | --- | --- | --- |
| P00561 | 2.7.2.4 | 1.1.1.3 | 1 | 1 |  |
| P00561 | 2.7.2.4 | 1.1.1.3 | 2.7.2.4 | 1 |  |
| **P00561 Total** |  |  |  | 2 | 0 |
| P27725 | 2.7.2.4 | 1.1.1.3 | 1 | 1 |  |
| P27725 | 2.7.2.4 | 1.1.1.3 | 2.7.2.4 | 1 |  |
| P27725 | 2.7.2.4 | 1.1.1.3 | 6.3.4.2 | 0 | 1 |
| **P27725 Total** |  |  |  | 2 | 1 |
| P44505 | 2.7.2.4 | 1.1.1.3 | 1 | 1 |  |
| **P44505 Total** |  |  |  | 1 | 0 |
| Q9K3D6 | 4.3.2.1 | 2.3.1.1 | 4 | 1 |  |
| Q9K3D6 | 4.3.2.1 | 2.3.1.1 | 4.3.2.1 | 27 |  |
| **Q9K3D6 Total** |  |  |  | 28 | 0 |
| Q9K3D7 | 4.3.2.1 | 2.3.1.1 | 4 | 1 |  |
| Q9K3D7 | 4.3.2.1 | 2.3.1.1 | 4.3.2.1 | 27 |  |
| **Q9K3D7 Total** |  |  |  | 28 | 0 |
| Q5E2E8 | 4.3.2.1 | 2.3.1 | 4 | 1 |  |
| Q5E2E8 | 4.3.2.1 | 2.3.1 | 4.3.2.1 | 22 |  |
| **Q5E2E8 Total** |  |  |  | 23 | 0 |
| P59620 | 4.3.2.1 | 2.3.1 | 4 | 1 |  |
| P59620 | 4.3.2.1 | 2.3.1 | 4.3.2.1 | 27 |  |
| **P59620 Total** |  |  |  | 28 | 0 |
| Q8DCM9 | 4.3.2.1 | 2.3.1 | 4 | 1 |  |
| Q8DCM9 | 4.3.2.1 | 2.3.1 | 4.3.2.1 | 27 |  |
| **Q8DCM9 Total** |  |  |  | 28 | 0 |
| Q7MH73 | 4.3.2.1 | 2.3.1 | 4 | 1 |  |
| Q7MH73 | 4.3.2.1 | 2.3.1 | 4.3.2.1 | 27 |  |
| **Q7MH73 Total** |  |  |  | 28 | 0 |
| Q8XDZ3 | 4.1.1 | 2.1.2 | 2.1.2.9 | 2 |  |
| **Q8XDZ3 Total** |  |  |  | 2 | 0 |
| P77398 | 4.1.1 | 2.1.2 | 2.1.2.9 | 2 |  |
| **P77398 Total** |  |  |  | 2 | 0 |
| Q8Z540 | 4.1.1 | 2.1.2 | 2 | 1 |  |
| Q8Z540 | 4.1.1 | 2.1.2 | 2.1.2.9 | 1 |  |
| Q8Z540 | 4.1.1 | 2.1.2 | 3 | 0 | 1 |
| **Q8Z540 Total** |  |  |  | 2 | 1 |
| O52325 | 4.1.1 | 2.1.2 | 2 | 1 |  |
| O52325 | 4.1.1 | 2.1.2 | 2.1.2.9 | 1 |  |
| O52325 | 4.1.1 | 2.1.2 | 3 | 0 | 1 |
| **O52325 Total** |  |  |  | 2 | 1 |
| Q8RF47 | 4.2.3.4 | 3.6.1 | 3.6.1.11 | 1 |  |
| Q8RF47 | 4.2.3.4 | 3.6.1 | 4.2.3.4 | 2 |  |
| **Q8RF47 Total** |  |  |  | 3 | 0 |
| Q8G5X4 | 2.7.1.71 | 4.2.3.4 | 4.2 | 1 |  |
| Q8G5X4 | 2.7.1.71 | 4.2.3.4 | 4.2.3.4 | 6 |  |
| **Q8G5X4 Total** |  |  |  | 7 | 0 |
| Q9WYI3 | 2.7.1.71 | 4.2.3.4 | 4 | 1 |  |
| Q9WYI3 | 2.7.1.71 | 4.2.3.4 | 4.2 | 1 |  |
| Q9WYI3 | 2.7.1.71 | 4.2.3.4 | 4.2.3.4 | 2 |  |
| **Q9WYI3 Total** |  |  |  | 4 | 0 |
| P52081 | 3.5.1.28 | 3.2.1.96 | 2.7.2.3 | 0 | 1 |
| P52081 | 3.5.1.28 | 3.2.1.96 | 3 | 1 |  |
| **P52081 Total** |  |  |  | 1 | 1 |
| Q9Y8G7 | 1.14.14.1 | 1.6.2.4 | 1 | 1 |  |
| Q9Y8G7 | 1.14.14.1 | 1.6.2.4 | 1.4 | 0 | 1 |
| **Q9Y8G7 Total** |  |  |  | 1 | 1 |
| P23473 | 3.2.1.14 | 3.2.1.17 | 3.2.1 | 1 |  |
| **P23473 Total** |  |  |  | 1 | 0 |
| Q13057 | 2.7.7.3 | 2.7.1.24 | 2 | 1 |  |
| **Q13057 Total** |  |  |  | 1 | 0 |
| Q9DBL7 | 2.7.7.3 | 2.7.1.24 | 1 | 0 | 1 |
| Q9DBL7 | 2.7.7.3 | 2.7.1.24 | 2 | 1 |  |
| Q9DBL7 | 2.7.7.3 | 2.7.1.24 | 3 | 0 | 1 |
| **Q9DBL7 Total** |  |  |  | 1 | 2 |
| P14779 | 1.14.14.1 | 1.6.2.4 | 1 | 4 |  |
| **P14779 Total** |  |  |  | 4 | 0 |
| Q9ACU1 | 2.5.1 | 2.5.1.31 | 2 | 2 |  |
| Q9ACU1 | 2.5.1 | 2.5.1.31 | 2.5.1.31 | 2 |  |
| **Q9ACU1 Total** |  |  |  | 4 | 0 |
| Q57506 | 4.6.1.1 |  | 3.6.3.14 | 0 | 1 |
| **Q57506 Total** |  |  |  | 0 | 1 |
| P15318 | 4.6.1.1 |  | 3.6.3.14 | 0 | 1 |
| **P15318 Total** |  |  |  | 0 | 1 |
| Q05762 | 1.5.1.3 | 2.1.1.45 | 2 | 3 |  |
| Q05762 | 1.5.1.3 | 2.1.1.45 | 2.1.1 | 1 |  |
| Q05762 | 1.5.1.3 | 2.1.1.45 | 2.1.1.45 | 8 |  |
| **Q05762 Total** |  |  |  | 12 | 0 |
| Q05763 | 1.5.1.3 | 2.1.1.45 | 2 | 2 |  |
| Q05763 | 1.5.1.3 | 2.1.1.45 | 2.1.1 | 1 |  |
| Q05763 | 1.5.1.3 | 2.1.1.45 | 2.1.1.45 | 8 |  |
| Q05763 | 1.5.1.3 | 2.1.1.45 | 5.1.1.7 | 0 | 1 |
| **Q05763 Total** |  |  |  | 11 | 1 |
| Q23695 | 1.5.1.3 | 2.1.1.45 | 2.1.1 | 1 |  |
| Q23695 | 1.5.1.3 | 2.1.1.45 | 2.1.1.45 | 5 |  |
| **Q23695 Total** |  |  |  | 6 | 0 |
| P45350 | 1.5.1.3 | 2.1.1.45 | 1.5.1.3 | 1 |  |
| P45350 | 1.5.1.3 | 2.1.1.45 | 2 | 3 |  |
| P45350 | 1.5.1.3 | 2.1.1.45 | 2.1.1.45 | 9 |  |
| P45350 | 1.5.1.3 | 2.1.1.45 | 5.1.1.7 | 0 | 1 |
| **P45350 Total** |  |  |  | 13 | 1 |
| P16126 | 1.5.1.3 | 2.1.1.45 | 2 | 2 |  |
| P16126 | 1.5.1.3 | 2.1.1.45 | 2.1 | 1 |  |
| P16126 | 1.5.1.3 | 2.1.1.45 | 2.1.1 | 1 |  |
| P16126 | 1.5.1.3 | 2.1.1.45 | 2.1.1.45 | 8 |  |
| **P16126 Total** |  |  |  | 12 | 0 |
| P07382 | 1.5.1.3 | 2.1.1.45 | 2 | 2 |  |
| P07382 | 1.5.1.3 | 2.1.1.45 | 2.1.1 | 1 |  |
| P07382 | 1.5.1.3 | 2.1.1.45 | 2.1.1.45 | 8 |  |
| P07382 | 1.5.1.3 | 2.1.1.45 | 6.1.1 | 0 | 1 |
| **P07382 Total** |  |  |  | 11 | 1 |
| O81395 | 1.5.1.3 | 2.1.1.45 | 1.5.1.3 | 1 |  |
| O81395 | 1.5.1.3 | 2.1.1.45 | 2 | 2 |  |
| O81395 | 1.5.1.3 | 2.1.1.45 | 2.1 | 1 |  |
| O81395 | 1.5.1.3 | 2.1.1.45 | 2.1.1 | 1 |  |
| O81395 | 1.5.1.3 | 2.1.1.45 | 2.1.1.45 | 9 |  |
| **O81395 Total** |  |  |  | 14 | 0 |
| Q5UQG3 | 1.5.1.3 | 2.1.1.45 | 2.1.1.45 | 1 |  |
| **Q5UQG3 Total** |  |  |  | 1 | 0 |
| Q27828 | 1.5.1.3 | 2.1.1.45 | 1.5.1.3 | 1 |  |
| Q27828 | 1.5.1.3 | 2.1.1.45 | 2 | 1 |  |
| Q27828 | 1.5.1.3 | 2.1.1.45 | 2.1.1 | 1 |  |
| Q27828 | 1.5.1.3 | 2.1.1.45 | 2.1.1.45 | 8 |  |
| **Q27828 Total** |  |  |  | 11 | 0 |
| Q27713 | 1.5.1.3 | 2.1.1.45 | 1.5.1.3 | 1 |  |
| Q27713 | 1.5.1.3 | 2.1.1.45 | 2 | 2 |  |
| Q27713 | 1.5.1.3 | 2.1.1.45 | 2.1 | 1 |  |
| Q27713 | 1.5.1.3 | 2.1.1.45 | 2.1.1 | 1 |  |
| Q27713 | 1.5.1.3 | 2.1.1.45 | 2.1.1.45 | 8 |  |
| **Q27713 Total** |  |  |  | 13 | 0 |
| P20712 | 1.5.1.3 | 2.1.1.45 | 2 | 2 |  |
| P20712 | 1.5.1.3 | 2.1.1.45 | 2.1 | 1 |  |
| P20712 | 1.5.1.3 | 2.1.1.45 | 2.1.1 | 1 |  |
| P20712 | 1.5.1.3 | 2.1.1.45 | 2.1.1.45 | 8 |  |
| **P20712 Total** |  |  |  | 12 | 0 |
| P13922 | 1.5.1.3 | 2.1.1.45 | 2 | 2 |  |
| P13922 | 1.5.1.3 | 2.1.1.45 | 2.1 | 1 |  |
| P13922 | 1.5.1.3 | 2.1.1.45 | 2.1.1 | 1 |  |
| P13922 | 1.5.1.3 | 2.1.1.45 | 2.1.1.45 | 8 |  |
| **P13922 Total** |  |  |  | 12 | 0 |
| O02604 | 1.5.1.3 | 2.1.1.45 | 2 | 1 |  |
| O02604 | 1.5.1.3 | 2.1.1.45 | 2.1 | 1 |  |
| O02604 | 1.5.1.3 | 2.1.1.45 | 2.1.1 | 1 |  |
| O02604 | 1.5.1.3 | 2.1.1.45 | 2.1.1.45 | 8 |  |
| O02604 | 1.5.1.3 | 2.1.1.45 | 4 | 0 | 1 |
| **O02604 Total** |  |  |  | 11 | 1 |
| P51820 | 1.5.1.3 | 2.1.1.45 | 2 | 3 |  |
| P51820 | 1.5.1.3 | 2.1.1.45 | 2.1.1 | 1 |  |
| P51820 | 1.5.1.3 | 2.1.1.45 | 2.1.1.45 | 11 |  |
| P51820 | 1.5.1.3 | 2.1.1.45 | 5.1.1.7 | 0 | 1 |
| **P51820 Total** |  |  |  | 15 | 1 |
| Q07422 | 1.5.1.3 | 2.1.1.45 | 1.1.1.1 | 0 | 1 |
| Q07422 | 1.5.1.3 | 2.1.1.45 | 2 | 1 |  |
| Q07422 | 1.5.1.3 | 2.1.1.45 | 2.1.1 | 1 |  |
| Q07422 | 1.5.1.3 | 2.1.1.45 | 2.1.1.45 | 7 |  |
| **Q07422 Total** |  |  |  | 9 | 1 |
| Q27783 | 1.5.1.3 | 2.1.1.45 | 2 | 2 |  |
| Q27783 | 1.5.1.3 | 2.1.1.45 | 2.1.1 | 1 |  |
| Q27783 | 1.5.1.3 | 2.1.1.45 | 2.1.1.45 | 7 |  |
| **Q27783 Total** |  |  |  | 10 | 0 |
| Q27793 | 1.5.1.3 | 2.1.1.45 | 2 | 2 |  |
| Q27793 | 1.5.1.3 | 2.1.1.45 | 2.1.1 | 1 |  |
| Q27793 | 1.5.1.3 | 2.1.1.45 | 2.1.1.45 | 7 |  |
| **Q27793 Total** |  |  |  | 10 | 0 |
| Q9CGE3 | 2.7.6.3 | 3.5.4.16 | 3.5.4.16 | 3 |  |
| **Q9CGE3 Total** |  |  |  | 3 | 0 |
| Q8GJP4 | 2.7.6.3 | 3.5.4.16 | 3.5.4.16 | 3 |  |
| **Q8GJP4 Total** |  |  |  | 3 | 0 |
| Q10663 | 4.1.3.1 | 2.3.3.9 | 2 | 1 |  |
| Q10663 | 4.1.3.1 | 2.3.3.9 | 2.3 | 1 |  |
| Q10663 | 4.1.3.1 | 2.3.3.9 | 2.3.3.9 | 5 |  |
| Q10663 | 4.1.3.1 | 2.3.3.9 | 4.1 | 1 |  |
| Q10663 | 4.1.3.1 | 2.3.3.9 | 4.1.3 | 1 |  |
| Q10663 | 4.1.3.1 | 2.3.3.9 | 4.1.3.1 | 4 |  |
| **Q10663 Total** |  |  |  | 13 | 0 |
| Q7TQ49 | 5.1.3.14 | 2.7.1.60 | 2.7.1 | 1 |  |
| **Q7TQ49 Total** |  |  |  | 1 | 0 |
| Q9Y223 | 5.1.3.14 | 2.7.1.60 | 2.7.1 | 1 |  |
| **Q9Y223 Total** |  |  |  | 1 | 0 |
| Q91WG8 | 5.1.3.14 | 2.7.1.60 | 2.7.1 | 1 |  |
| **Q91WG8 Total** |  |  |  | 1 | 0 |
| O35826 | 5.1.3.14 | 2.7.1.60 | 2.7.1 | 1 |  |
| **O35826 Total** |  |  |  | 1 | 0 |
| P17114 | 2.7.7.23 | 2.3.1.157 | 2.7.1.40 | 0 | 1 |
| **P17114 Total** |  |  |  | 0 | 1 |
| P43675 | 6.3.1.8 | 3.5.1.78 | 2 | 0 | 1 |
| **P43675 Total** |  |  |  | 0 | 1 |
| Q92G13 | 2.1.1 | 2.1.1.33 | 2 | 2 |  |
| Q92G13 | 2.1.1 | 2.1.1.33 | 2.1.1.33 | 2 |  |
| Q92G13 | 2.1.1 | 2.1.1.33 | 2.4 | 0 | 1 |
| Q92G13 | 2.1.1 | 2.1.1.33 | 3.1.3.48 | 0 | 1 |
| **Q92G13 Total** |  |  |  | 4 | 2 |
| Q9ZCB3 | 2.1.1 | 2.1.1.33 | 2 | 1 |  |
| Q9ZCB3 | 2.1.1 | 2.1.1.33 | 2.1.1.33 | 2 |  |
| **Q9ZCB3 Total** |  |  |  | 3 | 0 |
| Q83B60 | 2.7.1 | 2.7.7 | 2.1.2.9 | 0 | 1 |
| **Q83B60 Total** |  |  |  | 0 | 1 |
| Q7AAQ7 | 2.7.1 | 2.7.7 | 3.6.3 | 0 | 1 |
| **Q7AAQ7 Total** |  |  |  | 0 | 1 |
| Q8FDH5 | 2.7.1 | 2.7.7 | 3.6.3 | 0 | 1 |
| **Q8FDH5 Total** |  |  |  | 0 | 1 |
| P76658 | 2.7.1 | 2.7.7 | 3.6.3 | 0 | 1 |
| **P76658 Total** |  |  |  | 0 | 1 |
| Q74BF6 | 2.7.1 | 2.7.7 | 5 | 0 | 1 |
| Q74BF6 | 2.7.1 | 2.7.7 | 6 | 0 | 1 |
| **Q74BF6 Total** |  |  |  | 0 | 2 |
| Q9ZKZ0 | 2.7.1 | 2.7.7 | 1 | 0 | 1 |
| **Q9ZKZ0 Total** |  |  |  | 0 | 1 |
| Q9CME6 | 2.7.1 | 2.7.7 | 3.4.21 | 0 | 1 |
| **Q9CME6 Total** |  |  |  | 0 | 1 |
| Q88D93 | 2.7.1 | 2.7.7 | 2.7 | 1 |  |
| **Q88D93 Total** |  |  |  | 1 | 0 |
| Q87VF4 | 2.7.1 | 2.7.7 | 2.7 | 1 |  |
| **Q87VF4 Total** |  |  |  | 1 | 0 |
| Q98I54 | 2.7.1 | 2.7.7 | 1 | 0 | 1 |
| **Q98I54 Total** |  |  |  | 0 | 1 |
| Q6N2R5 | 2.7.1 | 2.7.7 | 1.18.6.1 | 0 | 1 |
| **Q6N2R5 Total** |  |  |  | 0 | 1 |
| Q8XEW9 | 2.7.1 | 2.7.7 | 3.6.3 | 0 | 1 |
| **Q8XEW9 Total** |  |  |  | 0 | 1 |
| Q7CPR9 | 2.7.1 | 2.7.7 | 3.6.3 | 0 | 1 |
| **Q7CPR9 Total** |  |  |  | 0 | 1 |
| Q7UBI8 | 2.7.1 | 2.7.7 | 3.6.3 | 0 | 1 |
| **Q7UBI8 Total** |  |  |  | 0 | 1 |
| Q9Z5B5 | 2.7.1 | 2.7.7 | 6.1.1.7 | 0 | 1 |
| **Q9Z5B5 Total** |  |  |  | 0 | 1 |
| Q8YD09 | 3.5.2.7 | 4.3.1.3 | 3 | 1 |  |
| Q8YD09 | 3.5.2.7 | 4.3.1.3 | 3.5.2.7 | 10 |  |
| Q8YD09 | 3.5.2.7 | 4.3.1.3 | 4 | 1 |  |
| Q8YD09 | 3.5.2.7 | 4.3.1.3 | 4.3.1 | 1 |  |
| Q8YD09 | 3.5.2.7 | 4.3.1.3 | 4.3.1.3 | 16 |  |
| **Q8YD09 Total** |  |  |  | 29 | 0 |
| Q58270 | 2.5.1.1 | 2.5.1.10 | 6.1.1.19 | 0 | 1 |
| **Q58270 Total** |  |  |  | 0 | 1 |
| Q58999 | 2.7.1.147 | 2.7.1.146 | 2 | 1 |  |
| Q58999 | 2.7.1.147 | 2.7.1.146 | 2.7.1 | 1 |  |
| Q58999 | 2.7.1.147 | 2.7.1.146 | 2.7.1.146 | 1 |  |
| **Q58999 Total** |  |  |  | 3 | 0 |
| Q55928 | 2.7.7.1 | 3.6.1 | 2 | 1 |  |
| Q55928 | 2.7.7.1 | 3.6.1 | 2.7.7.1 | 1 |  |
| **Q55928 Total** |  |  |  | 2 | 0 |
| O54820 | 2.7.7.4 | 2.7.1.25 | 2.7.1.25 | 1 |  |
| O54820 | 2.7.7.4 | 2.7.1.25 | 3.4.11.18 | 0 | 1 |
| O54820 | 2.7.7.4 | 2.7.1.25 | 6 | 0 | 1 |
| **O54820 Total** |  |  |  | 1 | 2 |
| O43252 | 2.7.7.4 | 2.7.1.25 | 2.7.1.25 | 1 |  |
| O43252 | 2.7.7.4 | 2.7.1.25 | 3.4.11.18 | 0 | 1 |
| O43252 | 2.7.7.4 | 2.7.1.25 | 6 | 0 | 1 |
| **O43252 Total** |  |  |  | 1 | 2 |
| Q60967 | 2.7.7.4 | 2.7.1.25 | 2.7.1.25 | 1 |  |
| Q60967 | 2.7.7.4 | 2.7.1.25 | 3.4.11.18 | 0 | 1 |
| Q60967 | 2.7.7.4 | 2.7.1.25 | 6 | 0 | 1 |
| **Q60967 Total** |  |  |  | 1 | 2 |
| O95340 | 2.7.7.4 | 2.7.1.25 | 2.7 | 1 |  |
| O95340 | 2.7.7.4 | 2.7.1.25 | 2.7.1.25 | 2 |  |
| O95340 | 2.7.7.4 | 2.7.1.25 | 6 | 0 | 1 |
| **O95340 Total** |  |  |  | 3 | 1 |
| O88428 | 2.7.7.4 | 2.7.1.25 | 2.7 | 1 |  |
| O88428 | 2.7.7.4 | 2.7.1.25 | 2.7.1.25 | 2 |  |
| O88428 | 2.7.7.4 | 2.7.1.25 | 6 | 0 | 1 |
| **O88428 Total** |  |  |  | 3 | 1 |
| Q27128 | 2.7.7.4 | 2.7.1.25 | 2.7 | 1 |  |
| Q27128 | 2.7.7.4 | 2.7.1.25 | 2.7.1 | 1 |  |
| Q27128 | 2.7.7.4 | 2.7.1.25 | 2.7.1.25 | 4 |  |
| Q27128 | 2.7.7.4 | 2.7.1.25 | 3.1 | 0 | 1 |
| Q27128 | 2.7.7.4 | 2.7.1.25 | 6 | 0 | 1 |
| **Q27128 Total** |  |  |  | 6 | 2 |
| P36204 | 2.7.2.3 | 5.3.1.1 | 2 | 4 |  |
| P36204 | 2.7.2.3 | 5.3.1.1 | 2.7 | 4 |  |
| P36204 | 2.7.2.3 | 5.3.1.1 | 2.7.2.3 | 29 |  |
| P36204 | 2.7.2.3 | 5.3.1.1 | 5.3.1.1 | 11 |  |
| **P36204 Total** |  |  |  | 48 | 0 |
| O13911 | 3.1.3.32 | 2.7.1.78 | 3 | 1 |  |
| O13911 | 3.1.3.32 | 2.7.1.78 | 3.1.3.18 | 0 | 1 |
| **O13911 Total** |  |  |  | 1 | 1 |
| Q96T60 | 3.1.3.32 | 2.7.1.78 | 1.17.4.3 | 0 | 1 |
| Q96T60 | 3.1.3.32 | 2.7.1.78 | 3 | 1 |  |
| **Q96T60 Total** |  |  |  | 1 | 1 |
| Q9JLV6 | 3.1.3.32 | 2.7.1.78 | 3 | 1 |  |
| Q9JLV6 | 3.1.3.32 | 2.7.1.78 | 3.1.3.18 | 0 | 1 |
| **Q9JLV6 Total** |  |  |  | 1 | 1 |
| P20772 | 6.3.4.13 | 6.3.3.1 | 2.6.1.52 | 0 | 1 |
| P20772 | 6.3.4.13 | 6.3.3.1 | 6 | 1 |  |
| P20772 | 6.3.4.13 | 6.3.3.1 | 6.3 | 1 |  |
| P20772 | 6.3.4.13 | 6.3.3.1 | 6.3.3.1 | 9 |  |
| P20772 | 6.3.4.13 | 6.3.3.1 | 6.3.4.13 | 6 |  |
| **P20772 Total** |  |  |  | 17 | 1 |
| Q99148 | 6.3.4.13 | 6.3.3.1 | 6 | 1 |  |
| Q99148 | 6.3.4.13 | 6.3.3.1 | 6.3 | 1 |  |
| Q99148 | 6.3.4.13 | 6.3.3.1 | 6.3.3.1 | 10 |  |
| Q99148 | 6.3.4.13 | 6.3.3.1 | 6.3.4.13 | 6 |  |
| **Q99148 Total** |  |  |  | 18 | 0 |
| P07244 | 6.3.4.13 | 6.3.3.1 | 6 | 1 |  |
| P07244 | 6.3.4.13 | 6.3.3.1 | 6.3.3.1 | 11 |  |
| P07244 | 6.3.4.13 | 6.3.3.1 | 6.3.4.13 | 4 |  |
| **P07244 Total** |  |  |  | 16 | 0 |
| Q8A155 | 2.1.2.3 | 3.5.4.10 | 2 | 1 |  |
| **Q8A155 Total** |  |  |  | 1 | 0 |
| Q89WU7 | 2.1.2.3 | 3.5.4.10 | 4.2.1.24 | 0 | 1 |
| **Q89WU7 Total** |  |  |  | 0 | 1 |
| P57143 | 2.1.2.3 | 3.5.4.10 | 2 | 1 |  |
| **P57143 Total** |  |  |  | 1 | 0 |
| Q8KA70 | 2.1.2.3 | 3.5.4.10 | 2 | 1 |  |
| **Q8KA70 Total** |  |  |  | 1 | 0 |
| Q9ABY4 | 2.1.2.3 | 3.5.4.10 | 4.2.1.24 | 0 | 1 |
| **Q9ABY4 Total** |  |  |  | 0 | 1 |
| P31335 | 2.1.2.3 | 3.5.4.10 | 2 | 1 |  |
| **P31335 Total** |  |  |  | 1 | 0 |
| Q892X3 | 2.1.2.3 | 3.5.4.10 | 2.7.1.37 | 0 | 1 |
| **Q892X3 Total** |  |  |  | 0 | 1 |
| Q9RHX6 | 2.1.2.3 | 3.5.4.10 | 3.6.3.14 | 0 | 1 |
| Q9RHX6 | 2.1.2.3 | 3.5.4.10 | 5.3.1.16 | 0 | 1 |
| **Q9RHX6 Total** |  |  |  | 0 | 2 |
| Q8X611 | 2.1.2.3 | 3.5.4.10 | 2 | 1 |  |
| Q8X611 | 2.1.2.3 | 3.5.4.10 | 2.5.1 | 0 | 1 |
| Q8X611 | 2.1.2.3 | 3.5.4.10 | 4.2.1.24 | 0 | 1 |
| Q8X611 | 2.1.2.3 | 3.5.4.10 | 5 | 0 | 1 |
| **Q8X611 Total** |  |  |  | 1 | 3 |
| Q8FB68 | 2.1.2.3 | 3.5.4.10 | 2 | 1 |  |
| Q8FB68 | 2.1.2.3 | 3.5.4.10 | 2.5.1 | 0 | 1 |
| Q8FB68 | 2.1.2.3 | 3.5.4.10 | 4.2.1.24 | 0 | 1 |
| Q8FB68 | 2.1.2.3 | 3.5.4.10 | 5 | 0 | 1 |
| **Q8FB68 Total** |  |  |  | 1 | 3 |
| P15639 | 2.1.2.3 | 3.5.4.10 | 2 | 1 |  |
| P15639 | 2.1.2.3 | 3.5.4.10 | 2.5.1 | 0 | 1 |
| P15639 | 2.1.2.3 | 3.5.4.10 | 4.2.1.24 | 0 | 1 |
| P15639 | 2.1.2.3 | 3.5.4.10 | 5 | 0 | 1 |
| **P15639 Total** |  |  |  | 1 | 3 |
| P43852 | 2.1.2.3 | 3.5.4.10 | 2 | 1 |  |
| P43852 | 2.1.2.3 | 3.5.4.10 | 4.2.1.24 | 0 | 1 |
| P43852 | 2.1.2.3 | 3.5.4.10 | 5.3.1.9 | 0 | 1 |
| **P43852 Total** |  |  |  | 1 | 2 |
| P31939 | 2.1.2.3 | 3.5.4.10 | 2 | 1 |  |
| P31939 | 2.1.2.3 | 3.5.4.10 | 5.99.1.3 | 0 | 1 |
| **P31939 Total** |  |  |  | 1 | 1 |
| Q9CWJ9 | 2.1.2.3 | 3.5.4.10 | 2 | 1 |  |
| **Q9CWJ9 Total** |  |  |  | 1 | 0 |
| P67542 | 2.1.2.3 | 3.5.4.10 | 6.1.1.20 | 0 | 1 |
| **P67542 Total** |  |  |  | 0 | 1 |
| Q9RAJ5 | 2.1.2.3 | 3.5.4.10 | 2.7.1.37 | 0 | 1 |
| Q9RAJ5 | 2.1.2.3 | 3.5.4.10 | 6.1.1.20 | 0 | 1 |
| **Q9RAJ5 Total** |  |  |  | 0 | 2 |
| P67541 | 2.1.2.3 | 3.5.4.10 | 6.1.1.20 | 0 | 1 |
| **P67541 Total** |  |  |  | 0 | 1 |
| P57828 | 2.1.2.3 | 3.5.4.10 | 2 | 1 |  |
| P57828 | 2.1.2.3 | 3.5.4.10 | 4.2.1.24 | 0 | 1 |
| **P57828 Total** |  |  |  | 1 | 1 |
| Q9HUV9 | 2.1.2.3 | 3.5.4.10 | 4.2.1.24 | 0 | 1 |
| **Q9HUV9 Total** |  |  |  | 0 | 1 |
| Q88DK3 | 2.1.2.3 | 3.5.4.10 | 4.2.1.24 | 0 | 1 |
| **Q88DK3 Total** |  |  |  | 0 | 1 |
| Q87VR9 | 2.1.2.3 | 3.5.4.10 | 4.2.1.24 | 0 | 1 |
| **Q87VR9 Total** |  |  |  | 0 | 1 |
| Q8Z335 | 2.1.2.3 | 3.5.4.10 | 2 | 1 |  |
| Q8Z335 | 2.1.2.3 | 3.5.4.10 | 2.5.1 | 0 | 1 |
| Q8Z335 | 2.1.2.3 | 3.5.4.10 | 4.2.1.24 | 0 | 1 |
| Q8Z335 | 2.1.2.3 | 3.5.4.10 | 5 | 0 | 1 |
| **Q8Z335 Total** |  |  |  | 1 | 3 |
| P26978 | 2.1.2.3 | 3.5.4.10 | 2 | 1 |  |
| P26978 | 2.1.2.3 | 3.5.4.10 | 2.5.1 | 0 | 1 |
| P26978 | 2.1.2.3 | 3.5.4.10 | 4.2.1.24 | 0 | 1 |
| P26978 | 2.1.2.3 | 3.5.4.10 | 5 | 0 | 1 |
| **P26978 Total** |  |  |  | 1 | 3 |
| O74928 | 2.1.2.3 | 3.5.4.10 | 4.2.1.11 | 0 | 1 |
| **O74928 Total** |  |  |  | 0 | 1 |
| Q5HH11 | 2.1.2.3 | 3.5.4.10 | 2.1 | 1 |  |
| **Q5HH11 Total** |  |  |  | 1 | 0 |
| P67543 | 2.1.2.3 | 3.5.4.10 | 2.1 | 1 |  |
| **P67543 Total** |  |  |  | 1 | 0 |
| P67544 | 2.1.2.3 | 3.5.4.10 | 2.1 | 1 |  |
| **P67544 Total** |  |  |  | 1 | 0 |
| Q6GI11 | 2.1.2.3 | 3.5.4.10 | 2.1 | 1 |  |
| **Q6GI11 Total** |  |  |  | 1 | 0 |
| Q6GAE0 | 2.1.2.3 | 3.5.4.10 | 2.1 | 1 |  |
| **Q6GAE0 Total** |  |  |  | 1 | 0 |
| Q8NX88 | 2.1.2.3 | 3.5.4.10 | 2.1 | 1 |  |
| **Q8NX88 Total** |  |  |  | 1 | 0 |
| P67545 | 2.1.2.3 | 3.5.4.10 | 4.2.1.24 | 0 | 1 |
| **P67545 Total** |  |  |  | 0 | 1 |
| P67546 | 2.1.2.3 | 3.5.4.10 | 4.2.1.24 | 0 | 1 |
| **P67546 Total** |  |  |  | 0 | 1 |
| Q8DWK8 | 2.1.2.3 | 3.5.4.10 | 4.2.1.24 | 0 | 1 |
| **Q8DWK8 Total** |  |  |  | 0 | 1 |
| Q8K8Y6 | 2.1.2.3 | 3.5.4.10 | 4.2.1.24 | 0 | 1 |
| **Q8K8Y6 Total** |  |  |  | 0 | 1 |
| Q5XEF2 | 2.1.2.3 | 3.5.4.10 | 4.2.1.24 | 0 | 1 |
| **Q5XEF2 Total** |  |  |  | 0 | 1 |
| Q8P310 | 2.1.2.3 | 3.5.4.10 | 4.2.1.24 | 0 | 1 |
| **Q8P310 Total** |  |  |  | 0 | 1 |
| Q97T99 | 2.1.2.3 | 3.5.4.10 | 4.2.1.24 | 0 | 1 |
| **Q97T99 Total** |  |  |  | 0 | 1 |
| Q8DRM1 | 2.1.2.3 | 3.5.4.10 | 4.2.1.24 | 0 | 1 |
| **Q8DRM1 Total** |  |  |  | 0 | 1 |
| Q9F1T4 | 2.1.2.3 | 3.5.4.10 | 4.2.1.24 | 0 | 1 |
| **Q9F1T4 Total** |  |  |  | 0 | 1 |
| Q9KV80 | 2.1.2.3 | 3.5.4.10 | 2 | 1 |  |
| Q9KV80 | 2.1.2.3 | 3.5.4.10 | 4.2.1.24 | 0 | 1 |
| **Q9KV80 Total** |  |  |  | 1 | 1 |
| Q5E257 | 2.1.2.3 | 3.5.4.10 | 4.2.1.24 | 0 | 1 |
| **Q5E257 Total** |  |  |  | 0 | 1 |
| Q87KT0 | 2.1.2.3 | 3.5.4.10 | 4.2.1.24 | 0 | 1 |
| **Q87KT0 Total** |  |  |  | 0 | 1 |
| Q8DD06 | 2.1.2.3 | 3.5.4.10 | 4.2.1.24 | 0 | 1 |
| **Q8DD06 Total** |  |  |  | 0 | 1 |
| Q7MGT5 | 2.1.2.3 | 3.5.4.10 | 4.2.1.24 | 0 | 1 |
| **Q7MGT5 Total** |  |  |  | 0 | 1 |
| Q8PQ19 | 2.1.2.3 | 3.5.4.10 | 4.2.1.24 | 0 | 1 |
| Q8PQ19 | 2.1.2.3 | 3.5.4.10 | 6.3.4.5 | 0 | 1 |
| **Q8PQ19 Total** |  |  |  | 0 | 2 |
| Q8PD47 | 2.1.2.3 | 3.5.4.10 | 4.2.1.24 | 0 | 1 |
| **Q8PD47 Total** |  |  |  | 0 | 1 |
| Q9PC10 | 2.1.2.3 | 3.5.4.10 | 2 | 1 |  |
| Q9PC10 | 2.1.2.3 | 3.5.4.10 | 4.2.1.24 | 0 | 1 |
| **Q9PC10 Total** |  |  |  | 1 | 1 |
| Q87D58 | 2.1.2.3 | 3.5.4.10 | 2 | 1 |  |
| Q87D58 | 2.1.2.3 | 3.5.4.10 | 4.2.1.24 | 0 | 1 |
| **Q87D58 Total** |  |  |  | 1 | 1 |
| Q8ZAR3 | 2.1.2.3 | 3.5.4.10 | 2 | 1 |  |
| Q8ZAR3 | 2.1.2.3 | 3.5.4.10 | 4.2.1.24 | 0 | 1 |
| **Q8ZAR3 Total** |  |  |  | 1 | 1 |
| P09546 | 1.5.99.8 | 1.5.1.12 | 1 | 1 |  |
| P09546 | 1.5.99.8 | 1.5.1.12 | 1.2.1 | 0 | 2 |
| **P09546 Total** |  |  |  | 1 | 2 |
| O52485 | 1.5.99.8 | 1.5.1.12 | 1 | 1 |  |
| O52485 | 1.5.99.8 | 1.5.1.12 | 1.2.1 | 0 | 2 |
| O52485 | 1.5.99.8 | 1.5.1.12 | 4.2.1.20 | 0 | 1 |
| **O52485 Total** |  |  |  | 1 | 3 |
| P95629 | 1.5.99.8 | 1.5.1.12 | 1 | 1 |  |
| P95629 | 1.5.99.8 | 1.5.1.12 | 1.2.1 | 0 | 1 |
| P95629 | 1.5.99.8 | 1.5.1.12 | 2 | 0 | 1 |
| **P95629 Total** |  |  |  | 1 | 2 |
| P10503 | 1.5.99.8 | 1.5.1.12 | 1 | 1 |  |
| P10503 | 1.5.99.8 | 1.5.1.12 | 1.2.1 | 0 | 1 |
| **P10503 Total** |  |  |  | 1 | 1 |
| Q7VJ82 | 2.7.7.6 |  | 1 | 0 | 1 |
| Q7VJ82 | 2.7.7.6 |  | 2 | 2 |  |
| Q7VJ82 | 2.7.7.6 |  | 2.4.1.1 | 0 | 1 |
| Q7VJ82 | 2.7.7.6 |  | 2.7 | 3 |  |
| Q7VJ82 | 2.7.7.6 |  | 2.7.7.6 | 9 |  |
| **Q7VJ82 Total** |  |  |  | 14 | 2 |
| Q9ZK23 | 2.7.7.6 |  | 1 | 0 | 2 |
| Q9ZK23 | 2.7.7.6 |  | 2 | 1 |  |
| Q9ZK23 | 2.7.7.6 |  | 2.4.1.1 | 0 | 1 |
| Q9ZK23 | 2.7.7.6 |  | 2.7 | 3 |  |
| Q9ZK23 | 2.7.7.6 |  | 2.7.7.6 | 9 |  |
| Q9ZK23 | 2.7.7.6 |  | 5.3.1.16 | 0 | 1 |
| Q9ZK23 | 2.7.7.6 |  | 6.1.1.4 | 0 | 1 |
| Q9ZK23 | 2.7.7.6 |  | 6.2.1.1 | 0 | 1 |
| **Q9ZK23 Total** |  |  |  | 13 | 6 |
| O25806 | 2.7.7.6 |  | 1 | 0 | 2 |
| O25806 | 2.7.7.6 |  | 2 | 1 |  |
| O25806 | 2.7.7.6 |  | 2.4.1.1 | 0 | 1 |
| O25806 | 2.7.7.6 |  | 2.7 | 3 |  |
| O25806 | 2.7.7.6 |  | 2.7.7.6 | 9 |  |
| O25806 | 2.7.7.6 |  | 5.3.1.16 | 0 | 1 |
| O25806 | 2.7.7.6 |  | 6.1.1.4 | 0 | 1 |
| **O25806 Total** |  |  |  | 13 | 5 |
| Q7MA56 | 2.7.7.6 |  | 1 | 0 | 1 |
| Q7MA56 | 2.7.7.6 |  | 2 | 1 |  |
| Q7MA56 | 2.7.7.6 |  | 2.7 | 3 |  |
| Q7MA56 | 2.7.7.6 |  | 2.7.7.6 | 9 |  |
| Q7MA56 | 2.7.7.6 |  | 6.2.1.1 | 0 | 1 |
| **Q7MA56 Total** |  |  |  | 13 | 2 |
| Q85FR6 | 2.7.7.6 |  | 2 | 2 |  |
| Q85FR6 | 2.7.7.6 |  | 2.7 | 4 |  |
| Q85FR6 | 2.7.7.6 |  | 2.7.1 | 0 | 1 |
| Q85FR6 | 2.7.7.6 |  | 2.7.7.6 | 24 |  |
| **Q85FR6 Total** |  |  |  | 30 | 1 |
| P28668 | 6.1.1.17 | 6.1.1.15 | 2 | 0 | 1 |
| P28668 | 6.1.1.17 | 6.1.1.15 | 2.4.2.29 | 0 | 1 |
| P28668 | 6.1.1.17 | 6.1.1.15 | 6 | 1 |  |
| P28668 | 6.1.1.17 | 6.1.1.15 | 6.1.1 | 3 |  |
| **P28668 Total** |  |  |  | 4 | 2 |
| P07814 | 6.1.1.17 | 6.1.1.15 | 2 | 0 | 1 |
| P07814 | 6.1.1.17 | 6.1.1.15 | 6 | 1 |  |
| P07814 | 6.1.1.17 | 6.1.1.15 | 6.1.1 | 2 |  |
| P07814 | 6.1.1.17 | 6.1.1.15 | 6.1.1.18 | 0 | 1 |
| **P07814 Total** |  |  |  | 3 | 2 |
| Q8CGC7 | 6.1.1.17 | 6.1.1.15 | 2 | 0 | 1 |
| Q8CGC7 | 6.1.1.17 | 6.1.1.15 | 6 | 1 |  |
| Q8CGC7 | 6.1.1.17 | 6.1.1.15 | 6.1.1 | 2 |  |
| Q8CGC7 | 6.1.1.17 | 6.1.1.15 | 6.1.1.18 | 0 | 1 |
| **Q8CGC7 Total** |  |  |  | 3 | 2 |
| Q58635 | 6.1.1.15 | 6.1.1.16 | 6.1.1 | 1 |  |
| **Q58635 Total** |  |  |  | 1 | 0 |
| P61422 | 2.5.1.3 | 2.7.4.7 | 2.5.1.3 | 1 |  |
| P61422 | 2.5.1.3 | 2.7.4.7 | 2.7.4.7 | 1 |  |
| **P61422 Total** |  |  |  | 2 | 0 |
| Q8YRC9 | 1.5.3 |  | 1 | 1 |  |
| **Q8YRC9 Total** |  |  |  | 1 | 0 |
| Q92F56 | 6.3.4 | 2.4.2.8 | 2 | 1 |  |
| Q92F56 | 6.3.4 | 2.4.2.8 | 2.4.2.8 | 4 |  |
| **Q92F56 Total** |  |  |  | 5 | 0 |
| Q724J4 | 6.3.4 | 2.4.2.8 | 2 | 1 |  |
| Q724J4 | 6.3.4 | 2.4.2.8 | 2.4.2.8 | 4 |  |
| **Q724J4 Total** |  |  |  | 5 | 0 |
| Q8YAC7 | 6.3.4 | 2.4.2.8 | 2 | 1 |  |
| Q8YAC7 | 6.3.4 | 2.4.2.8 | 2.4.2.8 | 4 |  |
| **Q8YAC7 Total** |  |  |  | 5 | 0 |
| Q8R6G8 | 2 | 2.1.1.33 | 2.1.1.33 | 1 |  |
| **Q8R6G8 Total** |  |  |  | 1 | 0 |
| P46843 | 1.8.1.9 | 1.6.4.5 | 1 | 1 |  |
| P46843 | 1.8.1.9 | 1.6.4.5 | 1.8.1.9 | 4 |  |
| **P46843 Total** |  |  |  | 5 | 0 |
| P31625 | 3.4.23 | 3.6.1.23 | 3.6.1.23 | 1 |  |
| **P31625 Total** |  |  |  | 1 | 0 |
| P29127 | 3.2.1.8 |  | 3.2.1.8 | 2 |  |
| P29127 | 3.2.1.8 |  | 6.3.5.2 | 0 | 2 |
| **P29127 Total** |  |  |  | 2 | 2 |
| P29126 | 3.2.1.8 |  | 3 | 1 |  |
| P29126 | 3.2.1.8 |  | 3.2.1.8 | 1 |  |
| **P29126 Total** |  |  |  | 2 | 0 |
| **Grand Total** |  |  |  | 719 | 132 |
