# Supplementary material for: Functional Representation of Enzymes by Specific Peptides
Source: PLoS Comput Biol. 2007 Aug 24;3(8):e167. doi: 10.1371/journal.pcbi.0030167 (PMC1950953; doi:10.1371/journal.pcbi.0030167)
Supplement: Supplementary file 3 [file pcbi.0030167.st003.doc]

**Table S3: Numbers of enzymes in Swiss-Prot release 48.3 and Swiss-Prot release 45.**

| **EC class** | **R45 ∩ R48** | **R48 ∩ not in R45** | **R45 ∩ not in R48** |
| --- | --- | --- | --- |
| **Oxidoreductases** | 7776 | 1661 | 142 |
| **Transferases** | 12474 | 3722 | 333 |
| **Hydrolases** | 8728 | 2173 | 254 |
| **Lyases** | 4140 | 1089 | 492 |
| **Isomerases** | 2348 | 541 | 33 |
| **Ligases** | 4649 | 1399 | 43 |
| **Total** | **40115** | **10585** | **1297** |
