# Supplementary material for: Functional Representation of Enzymes by Specific Peptides
Source: PLoS Comput Biol. 2007 Aug 24;3(8):e167. doi: 10.1371/journal.pcbi.0030167 (PMC1950953; doi:10.1371/journal.pcbi.0030167)
Supplement: Supplementary file 4 [file pcbi.0030167.st004.doc]

| subclass | SP avg | SP std | SW avg | SW std | #seqs |
| --- | --- | --- | --- | --- | --- |
| 1.1 | 0.944 | 0.009 | 0.932 | 0.013 | 2444 |
| 1.10 | 0.921 | 0.043 | 0.724 | 0.077 | 148 |
| 1.11 | 0.927 | 0.022 | 0.933 | 0.018 | 379 |
| 1.12 | 0.806 | 0.108 | 0.673 | 0.117 | 67 |
| 1.13 | 0.894 | 0.053 | 0.577 | 0.097 | 147 |
| 1.14 | 0.911 | 0.019 | 0.773 | 0.155 | 777 |
| 1.15 | 0.957 | 0.027 | 0.978 | 0.014 | 310 |
| 1.16 | 0.924 | 0.046 | 0.735 | 0.066 | 76 |
| 1.17 | 0.937 | 0.024 | 0.981 | 0.014 | 423 |
| 1.18 | 0.911 | 0.055 | 0.695 | 0.07 | 171 |
| 1.2 | 0.921 | 0.015 | 0.897 | 0.022 | 1013 |
| 1.3 | 0.889 | 0.029 | 0.696 | 0.038 | 641 |
| 1.4 | 0.929 | 0.027 | 0.737 | 0.056 | 299 |
| 1.5 | 0.898 | 0.047 | 0.399 | 0.062 | 218 |
| 1.6 | 0.936 | 0.015 | 0.835 | 0.029 | 970 |
| 1.7 | 0.924 | 0.042 | 0.641 | 0.059 | 196 |
| 1.8 | 0.933 | 0.026 | 0.824 | 0.036 | 513 |
| 1.9 | 0.951 | 0.016 | 0.942 | 0.016 | 575 |
| 1.97 | 0.941 | 0.081 | 0.673 | 0.168 | 34 |
|  |  |  |  |  |  |
| TOTAL | 0.929 | 0.021 | 0.845 | 0.147 |  |

**Table S4: Comparison of SP with Smith-Waterman performance on classification at the subclass level.**

Classification based on SPs has been compared with classification based on sequence similarity using the Smith-Waterman (SW) method. This has been performed on the oxidoreductases data of the 48.3 release, using all subclasses and sub-subclasses that contain more than 20 enzyme sequences. The data were randomly partitioned into 75% training and 25% test sets. Features for the SP classification were determined by running MEX on all oxidoreductases and checking for their specificity using the training data only. These SPs were then used for defining, through training, the SP-SVM. Smith-Waterman analysis was carried out by defining a log(p-value) (with cutoff at p=e-06) distance matrix whose columns (features) were all oxidoreductases. The rows (instances) of the training-set enzymes were used to determine the SW-SVM classifications. 45 different partitions were performed to accumulate statistics. Same partitions were applied to both classification methods. Classification was performed using a soft-margin linear SVM, available online at [http://svmlight.joachims.org](http://svmlight.joachims.org/). Performance was measured by the Jaccard score J=TP/(TP+FP+FN).
