# Supplementary material for: Functional Representation of Enzymes by Specific Peptides
Source: PLoS Comput Biol. 2007 Aug 24;3(8):e167. doi: 10.1371/journal.pcbi.0030167 (PMC1950953; doi:10.1371/journal.pcbi.0030167)
Supplement: Supplementary file 5 [file pcbi.0030167.st005.doc]

**Table S5: Comparison of SP with Smith-Waterman performance on classification at the sub-subclass level.**

| subsubclass | SP avg | SP std | SW avg | SW std | #seqs |
| --- | --- | --- | --- | --- | --- |
| 1.10.2 | 0.859 | 0.079 | 0.62 | 0.094 | 69 |
| 1.10.3 | 0.915 | 0.079 | 0.717 | 0.09 | 42 |
| 1.10.99 | 0.902 | 0.071 | 0.863 | 0.078 | 37 |
| 1.1.1 | 0.943 | 0.011 | 0.948 | 0.007 | 2309 |
| 1.11.1 | 0.92 | 0.026 | 0.937 | 0.015 | 379 |
| 1.12.99 | 0.862 | 0.115 | 0.542 | 0.292 | 30 |
| 1.13.11 | 0.89 | 0.074 | 0.628 | 0.06 | 128 |
| 1.14.11 | 0.878 | 0.101 | 0.318 | 0.095 | 64 |
| 1.14.12 | 0.882 | 0.079 | 0.838 | 0.077 | 63 |
| 1.14.13 | 0.853 | 0.063 | 0.442 | 0.089 | 163 |
| 1.14.14 | 0.912 | 0.032 | 0.892 | 0.02 | 248 |
| 1.14.17 | 0.96 | 0.088 | 0.643 | 0.087 | 32 |
| 1.14.18 | 0.84 | 0.159 | 0.31 | 0.246 | 26 |
| 1.14.19 | 0.919 | 0.102 | 0.922 | 0.079 | 29 |
| 1.14.99 | 0.912 | 0.053 | 0.31 | 0.061 | 90 |
| 1.15.1 | 0.954 | 0.028 | 0.987 | 0.009 | 310 |
| 1.17.1 | 0.963 | 0.033 | 0.98 | 0.014 | 151 |
| 1.17.4 | 0.914 | 0.027 | 0.98 | 0.016 | 271 |
| 1.18.1 | 0.862 | 0.116 | 0.21 | 0.067 | 46 |
| 1.18.6 | 0.925 | 0.042 | 0.953 | 0.037 | 125 |
| 1.1.99 | 0.932 | 0.068 | 0.525 | 0.065 | 75 |
| 1.2.1 | 0.944 | 0.02 | 0.952 | 0.009 | 825 |
| 1.2.4 | 0.874 | 0.076 | 0.902 | 0.079 | 86 |
| 1.2.7 | 0.879 | 0.099 | 0.265 | 0.131 | 56 |
| 1.2.99 | 0.65 | 0.161 | 0.617 | 0.109 | 39 |
| 1.3.1 | 0.834 | 0.045 | 0.642 | 0.057 | 233 |
| 1.3.3 | 0.922 | 0.026 | 0.913 | 0.043 | 267 |
| 1.3.5 | 0.88 | 0.129 | 0.2 | 0.163 | 23 |
| 1.3.7 | 0.895 | 0.188 | 0.967 | 0.075 | 25 |
| 1.3.99 | 0.835 | 0.078 | 0.148 | 0.035 | 93 |
| 1.4.1 | 0.952 | 0.045 | 0.927 | 0.071 | 99 |
| 1.4.3 | 0.856 | 0.075 | 0.407 | 0.061 | 104 |
| 1.4.99 | 0.964 | 0.048 | 0.735 | 0.062 | 46 |
| 1.5.1 | 0.892 | 0.049 | 0.563 | 0.1 | 168 |
| 1.6.1 | 0.898 | 0.126 | 0.208 | 0.164 | 26 |
| 1.6.2 | 0.898 | 0.116 | 0.167 | 0.137 | 23 |
| 1.6.5 | 0.888 | 0.023 | 0.885 | 0.026 | 713 |
| 1.6.99 | 0.734 | 0.081 | 0.242 | 0.067 | 208 |
| 1.7.1 | 0.97 | 0.034 | 0.878 | 0.049 | 94 |
| 1.7.2 | 0.929 | 0.102 | 0.395 | 0.15 | 35 |
| 1.8.1 | 0.917 | 0.039 | 0.893 | 0.045 | 239 |
| 1.9.3 | 0.957 | 0.017 | 0.938 | 0.004 | 575 |
| 1.97.1 | 0.89 | 0.116 | 0.682 | 0.114 | 34 |
|  |  |  |  |  |  |
|  |  |  |  |  |  |
|  |  |  |  |  |  |
| TOTAL | 0.916 | 0.047 | 0.833 | 0.231 |  |
