# Supplementary material for: Functional Representation of Enzymes by Specific Peptides
Source: PLoS Comput Biol. 2007 Aug 24;3(8):e167. doi: 10.1371/journal.pcbi.0030167 (PMC1950953; doi:10.1371/journal.pcbi.0030167)
Supplement: Supplementary file 6 [file pcbi.0030167.st006.doc]

**Table S6: List of SPs that lie in active pockets.**

A list of all events of SPs lying in active pockets that have passed the FDR=0.05 limit, ordered according to their p-values. Entries include the enzyme PDB-id and the details of the SP.

|  | P-value | PDB | SP |
| --- | --- | --- | --- |
| 1 | 0.00E+000 | 2cdhG | AAREGAS |
| 2 | 0.00E+000 | 2cdhG | GQANY |
| 3 | 0.00E+000 | 2cdhG | NNAGIT |
| 4 | 0.00E+000 | 2cdhG | TGASRG |
| 5 | 0.00E+000 | 2cdhG | VNNAGI |
| 6 | 0.00E+000 | 2cdhG | VNVVCPG |
| 7 | 0.00E+000 | 2cdhG | AAKAGVIG |
| 8 | 0.00E+000 | 2cdhG | GTIDVVVNNAGITRD |
| 9 | 0.00E+000 | 2cdhG | KGRIINIASVVGL |
| 10 | 0.00E+000 | 2cdhG | KVESPVVV |
| 11 | 0.00E+000 | 2cdhG | NYAAAKAG |
| 12 | 0.00E+000 | 2cdhG | QANYAA |
| 13 | 0.00E+000 | 2cdhG | RMKKSQW |
| 14 | 0.00E+000 | 2cdhG | VTGASRGIGKAIALSLGKAGCKVLVNYARS |
| 15 | 0.00E+000 | 2cdhG | YGGQAITFGGDVSK |
| 16 | 0.00E+000 | 1d7kA | AASTFNGF |
| 17 | 0.00E+000 | 1d7kA | ELMKVAR |
| 18 | 0.00E+000 | 1d7kA | GAYTVAAAS |
| 19 | 0.00E+000 | 1d7kA | GSDDEDES |
| 20 | 0.00E+000 | 1d7kA | KLKFEEIT |
| 21 | 0.00E+000 | 1d7kA | NDGVYG |
| 22 | 0.00E+000 | 1d7kA | RLSVKFGA |
| 23 | 0.00E+000 | 1d7kA | SQIKYAA |
| 24 | 0.00E+000 | 1d7kA | VINPALDKYFPSDSGVRIIAEPGRYYVASAFTLAVNIIAKK |
| 25 | 0.00E+000 | 1d7kA | YSSSIWGPTCDGLDRIVER |
| 26 | 0.00E+000 | 1d7kA | YYVNDG |
| 27 | 0.00E+000 | 2g76A | ASTKEA |
| 28 | 0.00E+000 | 2g76A | ARGGIVDE |
| 29 | 0.00E+000 | 2g76A | LISDSLDPCCRKILQDGGLQVVEKQNLSKEELIAELQDCEGLIVRSATKVTADVINAAEKLQVVGRAGTGVDNVDLEAATRKG |
| 30 | 0.00E+000 | 2g76A | LVMNTPNGNSLSAAELTCGM |
| 31 | 0.00E+000 | 1ae4 | ECHPY |
| 32 | 0.00E+000 | 1ae4 | GHPLYP |
| 33 | 0.00E+000 | 1ae4 | GYRHID |
| 34 | 0.00E+000 | 1ae4 | PLIGLGT |
| 35 | 0.00E+000 | 1ae4 | DLYLMHWP |
| 36 | 0.00E+000 | 1ae4 | GLGTWKS |
| 37 | 0.00E+000 | 1ae4 | IPKSVTPSRI |
| 38 | 0.00E+000 | 1ae4 | VTAYSPLGS |
| 39 | 0.00E+000 | 1ae4 | EPVLLEEPVV |
| 40 | 0.00E+000 | 1ae4 | GKAVPREELFVTSKLWNTKHHPEDVEPA |
| 41 | 0.00E+000 | 1ae4 | RSPAQILLRWQVQRKVICIPKS |
| 42 | 0.00E+000 | 1ae4 | VFDFTFSPEEMKQL |
| 43 | 0.00E+000 | 1tt9A | GEHPRMGALDVCPFIPVRGV |
| 44 | 0.00E+000 | 1tt9A | GPSTNRTVYTFVG |
| 45 | 0.00E+000 | 1tt9A | GVFGAYFNVLINL |
| 46 | 0.00E+000 | 1tt9A | LREQGRGKDQPGRLKKVQ |
| 47 | 0.00E+000 | 1tt9A | QTPGCVLLDVD |
| 48 | 0.00E+000 | 1qkiA | LALPPT |
| 49 | 0.00E+000 | 1qkiA | MVQNLM |
| 50 | 0.00E+000 | 1qkiA | TYKWVN |
| 51 | 0.00E+000 | 1qkiA | YFDEFG |
| 52 | 0.00E+000 | 1qkiA | GFQYEG |
| 53 | 0.00E+000 | 1qkiA | AYERLILDVFCG |
| 54 | 0.00E+000 | 1qkiA | DDPTVP |
| 55 | 0.00E+000 | 1qkiA | EKPFGRD |
| 56 | 0.00E+000 | 1qkiA | FGTEGR |
| 57 | 0.00E+000 | 1qkiA | FTPLLH |
| 58 | 0.00E+000 | 1qkiA | GASGDLAK |
| 59 | 0.00E+000 | 1qkiA | GRGGYFD |
| 60 | 0.00E+000 | 1qkiA | GTYKWV |
| 61 | 0.00E+000 | 1qkiA | GVPFIL |
| 62 | 0.00E+000 | 1qkiA | GYLDDPTV |
| 63 | 0.00E+000 | 1qkiA | IVEKPFG |
| 64 | 0.00E+000 | 1qkiA | IYPTIWWLF |
| 65 | 0.00E+000 | 1qkiA | MMTKKPGMFFNPEESELDLTYGN |
| 66 | 0.00E+000 | 1qkiA | NELVIR |
| 67 | 0.00E+000 | 1qkiA | PDAYERL |
| 68 | 0.00E+000 | 1qkiA | QIYRIDH |
| 69 | 0.00E+000 | 1qkiA | QNHLLQ |
| 70 | 0.00E+000 | 1qkiA | RDEKVKV |
| 71 | 0.00E+000 | 1qkiA | RIDHYLGKE |
| 72 | 0.00E+000 | 1qkiA | RNSYVAGQYD |
| 73 | 0.00E+000 | 1qkiA | VCGILREEL |
| 74 | 0.00E+000 | 1qkiA | VIRVQP |
| 75 | 0.00E+000 | 1qkiA | VLRFANR |
| 76 | 0.00E+000 | 1qkiA | VQNLMVLRF |
| 77 | 0.00E+000 | 1qkiA | WNRDNI |
| 78 | 0.00E+000 | 1qkiA | WWLFRD |
| 79 | 0.00E+000 | 1wmkA | LHDVYE |
| 80 | 0.00E+000 | 1wmkA | EFKNIFGTPEFVAPEIVNYEPLGLEADMWSIGVITYILLSGASPFLG |
| 81 | 0.00E+000 | 1wmkA | EYAAKFIKKR |
| 82 | 0.00E+000 | 1wmkA | GEELGSGQFAIV |
| 83 | 0.00E+000 | 1wmkA | TDVVLILELVSGGELFDFLA |
| 84 | 0.00E+000 | 1wmkA | TSELAKDFIR |
| 85 | 0.00E+000 | 1zmcA | SKALLNN |
| 86 | 0.00E+000 | 1zmcA | VIGAGVIG |
| 87 | 0.00E+000 | 1zmcA | VIGSGPGG |
| 88 | 0.00E+000 | 1zmcA | AVKALTGGIA |
| 89 | 0.00E+000 | 1zmcA | CLNVGCIP |
| 90 | 0.00E+000 | 1zmcA | EDIARVCHAHPT |
| 91 | 0.00E+000 | 1zmcA | GATKKSDG |
| 92 | 0.00E+000 | 1zmcA | GCIPSKAL |
| 93 | 0.00E+000 | 1zmcA | GGTCLN |
| 94 | 0.00E+000 | 1zmcA | GGYVAAI |
| 95 | 0.00E+000 | 1zmcA | GKFPFAANSRAKTNADTDGMVKILG |
| 96 | 0.00E+000 | 1zmcA | GPGGYV |
| 97 | 0.00E+000 | 1zmcA | HPTLSE |
| 98 | 0.00E+000 | 1zmcA | IKAAQLG |
| 99 | 0.00E+000 | 1zmcA | LALEYGAS |
| 100 | 0.00E+000 | 1zmcA | LGLEELG |
| 101 | 0.00E+000 | 1zmcA | PEVAWVG |
| 102 | 0.00E+000 | 1zmcA | PMLAHK |
| 103 | 0.00E+000 | 1zmcA | QRLGADVTA |
| 104 | 0.00E+000 | 1zmcA | TKNILIATGSEVTPFPG |
| 105 | 0.00E+000 | 1zmcA | VIYTHPE |
| 106 | 0.00E+000 | 1npdA | VGMKPLEN |
| 107 | 0.00E+000 | 1npdA | VSMPNK |
| 108 | 0.00E+000 | 1npdA | AIEGLKALKMRGTGVSMPNKQLACEYVDELTPAAKLVGAINTIVNDDGYLRGYNTDGTGHIRAIKESGFD |
| 109 | 0.00E+000 | 1npdA | GKTMVLLGAGGA |
| 110 | 0.00E+000 | 1npdA | LLVTECVYNPHMTKLLQQAQQAGCKTIDGYGMLLWQGAEQF |
| 111 | 0.00E+000 | 1npdA | YVKQVMGF |
| 112 | 2.25E-047 | 1occA | AELGQP |
| 113 | 2.25E-047 | 1occA | GCPPP |
| 114 | 2.25E-047 | 1occA | GKKEPF |
| 115 | 2.25E-047 | 1occA | HFHYV |
| 116 | 2.25E-047 | 1occA | LLLSLPV |
| 117 | 2.25E-047 | 1occA | NIKWSP |
| 118 | 2.25E-047 | 1occA | QYQTPL |
| 119 | 2.25E-047 | 1occA | VMPIM |
| 120 | 2.25E-047 | 1occA | WFFGH |
| 121 | 2.25E-047 | 1occA | ATMIIA |
| 122 | 2.25E-047 | 1occA | DMAFPR |
| 123 | 2.25E-047 | 1occA | DPAGGGDP |
| 124 | 2.25E-047 | 1occA | DQIYNV |
| 125 | 2.25E-047 | 1occA | DVDTRA |
| 126 | 2.25E-047 | 1occA | FGAWAG |
| 127 | 2.25E-047 | 1occA | FSWLAT |
| 128 | 2.25E-047 | 1occA | FVMIFF |
| 129 | 2.25E-047 | 1occA | FWLLPP |
| 130 | 2.25E-047 | 1occA | GAGTGW |
| 131 | 2.25E-047 | 1occA | GASVDL |
| 132 | 2.25E-047 | 1occA | GDPILYQHL |
| 133 | 2.25E-047 | 1occA | GGDPIL |
| 134 | 2.25E-047 | 1occA | GGFGNW |
| 135 | 2.25E-047 | 1occA | GGLTGI |
| 136 | 2.25E-047 | 1occA | GVSSILG |
| 137 | 2.25E-047 | 1occA | GYTLND |
| 138 | 2.25E-047 | 1occA | HDTYYVV |
| 139 | 2.25E-047 | 1occA | HFHYVL |
| 140 | 2.25E-047 | 1occA | HPEVYILI |
| 141 | 2.25E-047 | 1occA | HWFPLF |
| 142 | 2.25E-047 | 1occA | IGAPDM |
| 143 | 2.25E-047 | 1occA | IGGFGN |
| 144 | 2.25E-047 | 1occA | IIAIPT |
| 145 | 2.25E-047 | 1occA | INMKPP |
| 146 | 2.25E-047 | 1occA | IRAELGQPG |
| 147 | 2.25E-047 | 1occA | KKEPFGY |
| 148 | 2.25E-047 | 1occA | LLGDDQI |
| 149 | 2.25E-047 | 1occA | LLLASSMVE |
| 150 | 2.25E-047 | 1occA | LLLLASS |
| 151 | 2.25E-047 | 1occA | LLLLSLPVLA |
| 152 | 2.25E-047 | 1occA | LSLLIRAEL |
| 153 | 2.25E-047 | 1occA | LTTTNLEW |
| 154 | 2.25E-047 | 1occA | LYQHLF |
| 155 | 2.25E-047 | 1occA | MIFFM |
| 156 | 2.25E-047 | 1occA | NHKDIGTLY |
| 157 | 2.25E-047 | 1occA | NNMSFW |
| 158 | 2.25E-047 | 1occA | PLFVWS |
| 159 | 2.25E-047 | 1occA | PPPYHT |
| 160 | 2.25E-047 | 1occA | PPSFLLLLAS |
| 161 | 2.25E-047 | 1occA | QYQTPLF |
| 162 | 2.25E-047 | 1occA | RAYFT |
| 163 | 2.25E-047 | 1occA | SLHLAG |
| 164 | 2.25E-047 | 1occA | STNHK |
| 165 | 2.25E-047 | 1occA | VGMDVD |
| 166 | 2.25E-047 | 1occA | VTAHAF |
| 167 | 2.25E-047 | 1occA | VWAHHM |
| 168 | 2.25E-047 | 1occA | WAGMVG |
| 169 | 2.25E-047 | 1occA | WAKIHF |
| 170 | 2.25E-047 | 1occA | WAMMSI |
| 171 | 2.25E-047 | 1occA | WTVYPPL |
| 172 | 6.74E-035 | 1xfbA | TLLKPN |
| 173 | 6.74E-035 | 1xfbA | AMATVTAL |
| 174 | 6.74E-035 | 1xfbA | CPLPRPW |
| 175 | 6.74E-035 | 1xfbA | CQYVTEKVLAA |
| 176 | 6.74E-035 | 1xfbA | DFAKWRCVLKI |
| 177 | 6.74E-035 | 1xfbA | DHHVYLEGTLLKPNM |
| 178 | 6.74E-035 | 1xfbA | EENRRLYRQ |
| 179 | 6.74E-035 | 1xfbA | ENANVLARYA |
| 180 | 6.74E-035 | 1xfbA | ENTEENRR |
| 181 | 6.74E-035 | 1xfbA | ETLYQK |
| 182 | 6.74E-035 | 1xfbA | ETTTQG |
| 183 | 6.74E-035 | 1xfbA | FSYGRALQ |
| 184 | 6.74E-035 | 1xfbA | GIKVDKG |
| 185 | 6.74E-035 | 1xfbA | GILAADES |
| 186 | 6.74E-035 | 1xfbA | GIVVGIK |
| 187 | 6.74E-035 | 1xfbA | GRALQAS |
| 188 | 6.74E-035 | 1xfbA | GVVPLAGT |
| 189 | 6.74E-035 | 1xfbA | LEGTLLKPNMVT |
| 190 | 6.74E-035 | 1xfbA | LSGGQSE |
| 191 | 6.74E-035 | 1xfbA | NLNAIN |
| 192 | 6.74E-035 | 1xfbA | PDGDHDL |
| 193 | 6.74E-035 | 1xfbA | PPAVPG |
| 194 | 6.74E-035 | 1xfbA | PWALTFS |
| 195 | 6.74E-035 | 1xfbA | QGLDGL |
| 196 | 6.74E-035 | 1xfbA | RCAQYKKDG |
| 197 | 6.74E-035 | 1xfbA | VLAAVYKAL |
| 198 | 6.74E-035 | 1xfbA | YKALSDHHV |
| 199 | 2.30E-025 | 1gq2A | SPFDPV |
| 200 | 2.30E-025 | 1gq2A | VYTPT |
| 201 | 2.30E-025 | 1gq2A | ASGSPF |
| 202 | 2.30E-025 | 1gq2A | FNDDIQ |
| 203 | 2.30E-025 | 1gq2A | ILGLGD |
| 204 | 2.30E-025 | 1gq2A | IQFEDF |
| 205 | 2.30E-025 | 1gq2A | IVVTDG |
| 206 | 2.30E-025 | 1gq2A | LLKDPLY |
| 207 | 2.30E-025 | 1gq2A | LYTACGG |
| 208 | 2.30E-025 | 1gq2A | PGQGNN |
| 209 | 2.30E-025 | 1gq2A | QFEDFA |
| 210 | 2.30E-025 | 1gq2A | RNEKLFY |
| 211 | 2.30E-025 | 1gq2A | YTPTVG |
| 212 | 2.30E-025 | 1gq2A | DSKGLIV |
| 213 | 2.30E-025 | 1gq2A | FASGSPFDPV |
| 214 | 2.30E-025 | 1gq2A | FMPIVYTPTVGLACQ |
| 215 | 2.30E-025 | 1gq2A | FNDDIQGTASV |
| 216 | 2.30E-025 | 1gq2A | GMGIPVGKLALYTA |
| 217 | 2.30E-025 | 1gq2A | LAALRIT |
| 218 | 2.30E-025 | 1gq2A | LIQFEDFAN |
| 219 | 1.21E-024 | 1qk1A | PSAEYP |
| 220 | 1.21E-024 | 1qk1A | CPSNLGT |
| 221 | 1.21E-024 | 1qk1A | DLDASKI |
| 222 | 1.21E-024 | 1qk1A | IQTGVDNPG |
| 223 | 1.21E-024 | 1qk1A | LIDDHFLF |
| 224 | 1.21E-024 | 1qk1A | NEEDH |
| 225 | 1.21E-024 | 1qk1A | NERLGYI |
| 226 | 1.21E-024 | 1qk1A | RAERREVE |
| 227 | 1.21E-024 | 1qk1A | SSRVRTGR |
| 228 | 1.21E-024 | 1qk1A | DNPGHPFI |
| 229 | 1.21E-024 | 1qk1A | EQQQLIDDHFLFDKPVSPLL |
| 230 | 1.21E-024 | 1qk1A | ERGWEFMWNERLGY |
| 231 | 1.21E-024 | 1qk1A | GLKGDLAGRYY |
| 232 | 1.21E-024 | 1qk1A | IQTGVDNPGHP |
| 233 | 1.21E-024 | 1qk1A | LTCPSNLGTGLR |
| 234 | 1.21E-024 | 1qk1A | MKHTTDLDASKI |
| 235 | 1.21E-024 | 1qk1A | NEEDHTRVISMEKGGNMK |
| 236 | 1.21E-024 | 1qk1A | RHNGYDPR |
| 237 | 1.21E-024 | 1qk1A | YPDLRKHNNCMA |
| 238 | 1.21E-024 | 1qk1A | YVLSSRVRTGRSI |
| 239 | 1.01E-021 | 1atjA | SCPNV |
| 240 | 1.01E-021 | 1atjA | GGHTFGK |
| 241 | 1.01E-021 | 1atjA | LSGGHT |
| 242 | 1.01E-021 | 1atjA | ACPRTVSCAD |
| 243 | 1.01E-021 | 1atjA | DLVALSG |
| 244 | 1.01E-021 | 1atjA | DPRIAAS |
| 245 | 1.01E-021 | 1atjA | DQELFSSPNATDTIPLVR |
| 246 | 1.01E-021 | 1atjA | FDNKYYV |
| 247 | 1.01E-021 | 1atjA | FHDCF |
| 248 | 1.01E-021 | 1atjA | FIMDRLYNF |
| 249 | 1.01E-021 | 1atjA | HDCFV |
| 250 | 1.01E-021 | 1atjA | HTFGKNQC |
| 251 | 1.01E-021 | 1atjA | KGLIQSDQ |
| 252 | 1.01E-021 | 1atjA | NANSARG |
| 253 | 1.01E-021 | 1atjA | NLPAPFFTL |
| 254 | 1.01E-021 | 1atjA | PDPTLN |
| 255 | 1.01E-021 | 1atjA | PLTGTQG |
| 256 | 1.01E-021 | 1atjA | QSDQELFS |
| 257 | 1.01E-021 | 1atjA | SFRTEKDA |
| 258 | 1.01E-021 | 1atjA | VPLGRRD |
| 259 | 4.89E-021 | 1aa1B | ETGEIK |
| 260 | 4.89E-021 | 1aa1B | ALYKAQ |
| 261 | 4.89E-021 | 1aa1B | ALRLEDLRIP |
| 262 | 4.89E-021 | 1aa1B | AYVKTF |
| 263 | 4.89E-021 | 1aa1B | CYHIEPV |
| 264 | 4.89E-021 | 1aa1B | DRYKGR |
| 265 | 4.89E-021 | 1aa1B | FRVLAK |
| 266 | 4.89E-021 | 1aa1B | GEENQYI |
| 267 | 4.89E-021 | 1aa1B | GFTANT |
| 268 | 4.89E-021 | 1aa1B | GNVFGFKA |
| 269 | 4.89E-021 | 1aa1B | HRAMHA |
| 270 | 4.89E-021 | 1aa1B | IEPVAGEE |
| 271 | 4.89E-021 | 1aa1B | KAQAETGE |
| 272 | 4.89E-021 | 1aa1B | KPKLGLS |
| 273 | 4.89E-021 | 1aa1B | KTFQGP |
| 274 | 4.89E-021 | 1aa1B | LFCAEA |
| 275 | 4.89E-021 | 1aa1B | LLHIHRAMH |
| 276 | 4.89E-021 | 1aa1B | LRGGLDFTKDDEN |
| 277 | 4.89E-021 | 1aa1B | LRLSGGD |
| 278 | 4.89E-021 | 1aa1B | LTYYTP |
| 279 | 4.89E-021 | 1aa1B | MHDYLT |
| 280 | 4.89E-021 | 1aa1B | PIVMHD |
| 281 | 4.89E-021 | 1aa1B | QAETGEIK |
| 282 | 4.89E-021 | 1aa1B | QKNHG |
| 283 | 4.89E-021 | 1aa1B | QYICY |
| 284 | 4.89E-021 | 1aa1B | RALRLED |
| 285 | 4.89E-021 | 1aa1B | SGGDHIH |
| 286 | 4.89E-021 | 1aa1B | TWTTVWTD |
| 287 | 4.89E-021 | 1aa1B | YKGRCY |
| 288 | 4.89E-021 | 1aa1B | YYTPEY |
| 289 | 7.61E-021 | 1l1fA | ASEKQLT |
| 290 | 7.61E-021 | 1l1fA | ENFINE |
| 291 | 7.61E-021 | 1l1fA | SYFEW |
| 292 | 7.61E-021 | 1l1fA | ANGPTT |
| 293 | 7.61E-021 | 1l1fA | GGVTVSYFEW |
| 294 | 7.61E-021 | 1l1fA | MTYKCA |
| 295 | 7.61E-021 | 1l1fA | NAGGVTVS |
| 296 | 7.61E-021 | 1l1fA | QGFGNVG |
| 297 | 7.61E-021 | 1l1fA | DCDILIPAA |
| 298 | 7.61E-021 | 1l1fA | DINAHACVTGKPI |
| 299 | 7.61E-021 | 1l1fA | DVPAPDM |
| 300 | 7.61E-021 | 1l1fA | EEQKRNRVR |
| 301 | 7.61E-021 | 1l1fA | ELAKKGFIGPG |
| 302 | 7.61E-021 | 1l1fA | FEWLKNLNHVSYGRLTFKYER |
| 303 | 7.61E-021 | 1l1fA | GASEKDIVHS |
| 304 | 7.61E-021 | 1l1fA | ISQGGIHGRISATGRG |
| 305 | 7.61E-021 | 1l1fA | KAKIIAEGANGPTTP |
| 306 | 7.61E-021 | 1l1fA | MVEGFFDRGASIVEDKLV |
| 307 | 7.61E-021 | 1l1fA | QGFGNVGLHSMRYLHRFGAKC |
| 308 | 7.61E-021 | 1l1fA | RTAAYVNAIEKVFKVY |
| 309 | 7.61E-021 | 1l1fA | RTPCKGGIRYS |
| 310 | 7.61E-021 | 1l1fA | SATGRGVFHG |
| 311 | 7.61E-021 | 1l1fA | TAMKYNLGLDLRTAAYVN |
| 312 | 7.61E-021 | 1l1fA | TGEREMSWIADTYA |
| 313 | 7.61E-021 | 1l1fA | VDVPFGGAKAG |
| 314 | 7.61E-021 | 1l1fA | VPTAEFQD |
| 315 | 7.61E-021 | 1l1fA | YTMERSAR |
| 316 | 3.27E-018 | 1gytA | AEGRLV |
| 317 | 3.27E-018 | 1gytA | DIAGTAW |
| 318 | 3.27E-018 | 1gytA | NTDAEGRL |
| 319 | 3.27E-018 | 1gytA | AEQLDKISDGYIS |
| 320 | 3.27E-018 | 1gytA | AYLASQAR |
| 321 | 3.27E-018 | 1gytA | DRAWRLPL |
| 322 | 3.27E-018 | 1gytA | DVATLTGACVIALGHHI |
| 323 | 3.27E-018 | 1gytA | FADMANIGGRPGGAITA |
| 324 | 3.27E-018 | 1gytA | GATGRPV |
| 325 | 3.27E-018 | 1gytA | GKPGQTLLL |
| 326 | 3.27E-018 | 1gytA | IQKTINTLN |
| 327 | 3.27E-018 | 1gytA | ITAGCFLSRF |
| 328 | 3.27E-018 | 1gytA | LTYVERFEPE |
| 329 | 3.27E-018 | 1gytA | NHNPLAHEL |
| 330 | 3.27E-018 | 1gytA | TGSMEAVCFLTELHVKGR |
| 331 | 3.27E-018 | 1gytA | TMSGQT |
| 332 | 3.27E-018 | 1gytA | VFNVPTRREL |
| 333 | 3.27E-018 | 1gytA | YWKVRQAVE |
| 334 | 1.20E-017 | 1q2lA | DSALAAV |
| 335 | 1.20E-017 | 1q2lA | HQAVVE |
| 336 | 1.20E-017 | 1q2lA | SGVLAIS |
| 337 | 1.20E-017 | 1q2lA | STDSALA |
| 338 | 1.20E-017 | 1q2lA | TNANNG |
| 339 | 1.20E-017 | 1q2lA | VQQALKD |
| 340 | 1.20E-017 | 1q2lA | DRGNMR |
| 341 | 1.20E-017 | 1q2lA | ALLEGYFSY |
| 342 | 1.20E-017 | 1q2lA | AMDSARNQV |
| 343 | 1.20E-017 | 1q2lA | ANGYTQRLPQLF |
| 344 | 1.20E-017 | 1q2lA | AQIKLLTPQKLADFFHQAVVEPQGMAILSQI |
| 345 | 1.20E-017 | 1q2lA | DFRYPSITRDMDYVEWLADTMIRVPV |
| 346 | 1.20E-017 | 1q2lA | FALNDYLAG |
| 347 | 1.20E-017 | 1q2lA | FRSKTDEL |
| 348 | 1.20E-017 | 1q2lA | FSGGNLETLSDKPGNPVQQAL |
| 349 | 1.20E-017 | 1q2lA | HGGSHNASTAPYRTAFYLEVENDALPGAVDRLADAIA |
| 350 | 1.20E-017 | 1q2lA | KKYAERERNAVNAELTMARTRDGMRMAQVSAETINPAHPGS |
| 351 | 1.20E-017 | 1q2lA | LGQIVQPWFYNQLRTEEQLGYAVFAFPMSVGRQWGMGFLLQSNDKQPS |
| 352 | 1.20E-017 | 1q2lA | LMGSKKYPQADSLAEYLK |
| 353 | 1.20E-017 | 1q2lA | LPSITLKEV |
| 354 | 1.20E-017 | 1q2lA | LREKGIDK |
| 355 | 1.20E-017 | 1q2lA | LSKDFDRGNMRFDSRDKI |
| 356 | 1.20E-017 | 1q2lA | LTDKGLANRD |
| 357 | 1.20E-017 | 1q2lA | MVVLLVSDPQAVKSLSALVVPV |
| 358 | 1.20E-017 | 1q2lA | NIADRYD |
| 359 | 1.20E-017 | 1q2lA | QGLAHYLEHM |
| 360 | 1.20E-017 | 1q2lA | RLAMMTPQNARIWYISP |
| 361 | 1.20E-017 | 1q2lA | SDPIVNGNSGV |
| 362 | 1.20E-017 | 1q2lA | SQVPYFSRDERR |
| 363 | 1.20E-017 | 1q2lA | VVAAIFSYLN |
| 364 | 1.20E-017 | 1q2lA | YLIGNRSPGTLSDWLQKQGLVEGISA |
| 365 | 1.53E-017 | 1gk8A | PIDLFE |
| 366 | 1.53E-017 | 1gk8A | AAESSTGT |
| 367 | 1.53E-017 | 1gk8A | AGVKDY |
| 368 | 1.53E-017 | 1gk8A | AYVKTF |
| 369 | 1.53E-017 | 1gk8A | CRDNG |
| 370 | 1.53E-017 | 1gk8A | DLFEEGS |
| 371 | 1.53E-017 | 1gk8A | DRYKGR |
| 372 | 1.53E-017 | 1gk8A | GFTANT |
| 373 | 1.53E-017 | 1gk8A | GNVFGFKA |
| 374 | 1.53E-017 | 1gk8A | HGIHFRVL |
| 375 | 1.53E-017 | 1gk8A | HRAMHA |
| 376 | 1.53E-017 | 1gk8A | IQVERD |
| 377 | 1.53E-017 | 1gk8A | KAQAETGE |
| 378 | 1.53E-017 | 1gk8A | KPKLGLS |
| 379 | 1.53E-017 | 1gk8A | LFEEGSVTN |
| 380 | 1.53E-017 | 1gk8A | LLHIHRAMH |
| 381 | 1.53E-017 | 1gk8A | LRGGLDFTKDDEN |
| 382 | 1.53E-017 | 1gk8A | MHDYLT |
| 383 | 1.53E-017 | 1gk8A | QVERDK |
| 384 | 1.53E-017 | 1gk8A | RWRDRF |
| 385 | 1.53E-017 | 1gk8A | TCEEM |
| 386 | 1.53E-017 | 1gk8A | TWTTVWTD |
| 387 | 1.53E-017 | 1gk8A | VASGGIH |
| 388 | 1.53E-017 | 1gk8A | VERDKL |
| 389 | 1.53E-017 | 1gk8A | VGKLEG |
| 390 | 1.53E-017 | 1gk8A | WRDRFLF |
| 391 | 8.97E-017 | 2hakA | AKFRQI |
| 392 | 8.97E-017 | 2hakA | GSLPFD |
| 393 | 8.97E-017 | 2hakA | QNLKELR |
| 394 | 8.97E-017 | 2hakA | VWSLG |
| 395 | 8.97E-017 | 2hakA | DVWSLG |
| 396 | 8.97E-017 | 2hakA | LYLVME |
| 397 | 8.97E-017 | 2hakA | VDVWS |
| 398 | 8.97E-017 | 2hakA | VHRDLK |
| 399 | 8.97E-017 | 2hakA | WSLGVI |
| 400 | 8.97E-017 | 2hakA | GEVFDYLV |
| 401 | 8.97E-017 | 2hakA | GPEVDVWS |
| 402 | 8.97E-017 | 2hakA | GSPPYA |
| 403 | 8.97E-017 | 2hakA | GVILYTLV |
| 404 | 8.97E-017 | 2hakA | IVSAVQYCHQK |
| 405 | 8.97E-017 | 2hakA | KRIDIMV |
| 406 | 8.97E-017 | 2hakA | LNHPNIVKLFEVIETEKTLYL |
| 407 | 8.97E-017 | 2hakA | LVLNPIKRG |
| 408 | 8.97E-017 | 2hakA | NLKELRERVLRGKYR |
| 409 | 8.97E-017 | 2hakA | PFYMSTDCE |
| 410 | 8.97E-017 | 2hakA | SLQKLFREVRIMK |
| 411 | 8.97E-017 | 2hakA | TFCGS |
| 412 | 4.01E-016 | 1eq2A | FQAVADA |
| 413 | 4.01E-016 | 1eq2A | LNNGES |
| 414 | 4.01E-016 | 1eq2A | NNGESP |
| 415 | 4.01E-016 | 1eq2A | YMDKE |
| 416 | 4.01E-016 | 1eq2A | RYFNV |
| 417 | 4.01E-016 | 1eq2A | SSAATYG |
| 418 | 4.01E-016 | 1eq2A | DILVVDNLK |
| 419 | 4.01E-016 | 1eq2A | FHEGACS |
| 420 | 4.01E-016 | 1eq2A | FKTVAEG |
| 421 | 4.01E-016 | 1eq2A | GACSSTTEW |
| 422 | 4.01E-016 | 1eq2A | GSENFKRDF |
| 423 | 4.01E-016 | 1eq2A | IADYMDKE |
| 424 | 4.01E-016 | 1eq2A | LKGRYQ |
| 425 | 4.01E-016 | 1eq2A | LNVYGYSK |
| 426 | 4.01E-016 | 1eq2A | MASVAFH |
| 427 | 4.01E-016 | 1eq2A | NLVDLNIADY |
| 428 | 4.01E-016 | 1eq2A | NNYQY |
| 429 | 4.01E-016 | 1eq2A | PFLYASSAA |
| 430 | 4.01E-016 | 1eq2A | PKLFEGS |
| 431 | 4.01E-016 | 1eq2A | RYFNVYGP |
| 432 | 4.01E-016 | 1eq2A | TTEWDGKY |
| 433 | 4.01E-016 | 1eq2A | VADATLAYHKKG |
| 434 | 4.01E-016 | 1eq2A | YFNVYGPRE |
| 435 | 4.01E-016 | 1eq2A | YGYSKFLFDEYVR |
| 436 | 4.01E-016 | 1eq2A | YMMDNN |
| 437 | 6.61E-016 | 1f1hA | GKMFDG |
| 438 | 6.61E-016 | 1f1hA | LYDLPP |
| 439 | 6.61E-016 | 1f1hA | DIRSEMCL |
| 440 | 6.61E-016 | 1f1hA | GKEQHVT |
| 441 | 6.61E-016 | 1f1hA | GKMFDGSS |
| 442 | 6.61E-016 | 1f1hA | GNKGHRP |
| 443 | 6.61E-016 | 1f1hA | GPEPEF |
| 444 | 6.61E-016 | 1f1hA | INESDM |
| 445 | 6.61E-016 | 1f1hA | KGGYFP |
| 446 | 6.61E-016 | 1f1hA | KYVVHNVA |
| 447 | 6.61E-016 | 1f1hA | MGLVVEAHHHE |
| 448 | 6.61E-016 | 1f1hA | NGSGMH |
| 449 | 6.61E-016 | 1f1hA | NRSASIRIP |
| 450 | 6.61E-016 | 1f1hA | NSYKRL |
| 451 | 6.61E-016 | 1f1hA | RLVPGYEAP |
| 452 | 6.61E-016 | 1f1hA | SIGGWK |
| 453 | 6.61E-016 | 1f1hA | SISGSHVAIDDIEGAWNSST |
| 454 | 6.61E-016 | 1f1hA | TIPAHQVN |
| 455 | 1.71E-014 | 1hwxA | AQHSQH |
| 456 | 1.71E-014 | 1hwxA | ASEKQLT |
| 457 | 1.71E-014 | 1hwxA | IVEDKL |
| 458 | 1.71E-014 | 1hwxA | SYFEW |
| 459 | 1.71E-014 | 1hwxA | GGVTVSYFEW |
| 460 | 1.71E-014 | 1hwxA | GYRAQH |
| 461 | 1.71E-014 | 1hwxA | MTYKCA |
| 462 | 1.71E-014 | 1hwxA | DCDILIPAA |
| 463 | 1.71E-014 | 1hwxA | DDPNFF |
| 464 | 1.71E-014 | 1hwxA | DGSIWNPDGIDPKELED |
| 465 | 1.71E-014 | 1hwxA | DINAHACVTGKPI |
| 466 | 1.71E-014 | 1hwxA | EEQKRNRVR |
| 467 | 1.71E-014 | 1hwxA | ELAKKGFIGPG |
| 468 | 1.71E-014 | 1hwxA | ISQGGIHGRISATGRG |
| 469 | 1.71E-014 | 1hwxA | KAKIIAEGANGPTTP |
| 470 | 1.71E-014 | 1hwxA | MVEGFFDRGASIVEDKLV |
| 471 | 1.71E-014 | 1hwxA | QGFGNVGLHSMRYLHRFGAKC |
| 472 | 1.71E-014 | 1hwxA | RRHYSE |
| 473 | 1.71E-014 | 1hwxA | RTPCKGGIRYS |
| 474 | 1.71E-014 | 1hwxA | SATGRGVFHG |
| 475 | 1.71E-014 | 1hwxA | VDVPFGGAKAG |
| 476 | 1.71E-014 | 1hwxA | VPTAEFQD |
| 477 | 1.71E-014 | 1hwxA | WEVIEGYRAQHS |
| 478 | 3.28E-014 | 1yg6B | IYSRLL |
| 479 | 3.28E-014 | 1yg6B | NSPGG |
| 480 | 3.28E-014 | 1yg6B | AASMGAFLL |
| 481 | 3.28E-014 | 1yg6B | AQMLFLEAE |
| 482 | 3.28E-014 | 1yg6B | ATDIEI |
| 483 | 3.28E-014 | 1yg6B | DIEIHA |
| 484 | 3.28E-014 | 1yg6B | DIYSRLL |
| 485 | 3.28E-014 | 1yg6B | DTERDRF |
| 486 | 3.28E-014 | 1yg6B | EQTSRGER |
| 487 | 3.28E-014 | 1yg6B | ERVIFL |
| 488 | 3.28E-014 | 1yg6B | GQAASMG |
| 489 | 3.28E-014 | 1yg6B | GQATDI |
| 490 | 3.28E-014 | 1yg6B | HQPLGG |
| 491 | 3.28E-014 | 1yg6B | INSPGG |
| 492 | 3.28E-014 | 1yg6B | LPNSRV |
| 493 | 3.28E-014 | 1yg6B | MANLIVAQ |
| 494 | 3.28E-014 | 1yg6B | MIHQP |
| 495 | 3.28E-014 | 1yg6B | MNELMA |
| 496 | 3.28E-014 | 1yg6B | PMVIEQ |
| 497 | 3.28E-014 | 1yg6B | VEDHMA |
| 498 | 3.28E-014 | 1yg6B | VIEQTSRGE |
| 499 | 1.26E-013 | 1f76A | KAHYDG |
| 500 | 1.26E-013 | 1f76A | KNKDTP |
| 501 | 1.26E-013 | 1f76A | TGGLSG |
| 502 | 1.26E-013 | 1f76A | YGEALD |
| 503 | 1.26E-013 | 1f76A | AAGLDK |
| 504 | 1.26E-013 | 1f76A | AEGLINR |
| 505 | 1.26E-013 | 1f76A | ALDDLLTAIKNKQNDLQ |
| 506 | 1.26E-013 | 1f76A | AVKIAPDL |
| 507 | 1.26E-013 | 1f76A | CMGLTF |
| 508 | 1.26E-013 | 1f76A | EALVRQKVP |
| 509 | 1.26E-013 | 1f76A | EEELIQVADSL |
| 510 | 1.26E-013 | 1f76A | GLRTLQYG |
| 511 | 1.26E-013 | 1f76A | GNDKPRLFR |
| 512 | 1.26E-013 | 1f76A | HHKYVP |
| 513 | 1.26E-013 | 1f76A | IGKNKDT |
| 514 | 1.26E-013 | 1f76A | IGVGGIDS |
| 515 | 1.26E-013 | 1f76A | LQYGEAL |
| 516 | 1.26E-013 | 1f76A | NISSPN |
| 517 | 1.26E-013 | 1f76A | NRMGFNN |
| 518 | 1.26E-013 | 1f76A | PIIGVGG |
| 519 | 1.26E-013 | 1f76A | TFQQLRRIT |
| 520 | 1.26E-013 | 1f76A | TNTTLDR |
| 521 | 1.26E-013 | 1f76A | VIATNTT |
| 522 | 3.05E-011 | 1b3rA | CNIFS |
| 523 | 3.05E-011 | 1b3rA | FSTQDH |
| 524 | 3.05E-011 | 1b3rA | SVTKSK |
| 525 | 3.05E-011 | 1b3rA | AGKVAVV |
| 526 | 3.05E-011 | 1b3rA | EETTTG |
| 527 | 3.05E-011 | 1b3rA | EGRLVNL |
| 528 | 3.05E-011 | 1b3rA | GHPSFVMS |
| 529 | 3.05E-011 | 1b3rA | GRLVNLGCA |
| 530 | 3.05E-011 | 1b3rA | GVHNLYKM |
| 531 | 3.05E-011 | 1b3rA | IKPQVD |
| 532 | 3.05E-011 | 1b3rA | ITEIDPI |
| 533 | 3.05E-011 | 1b3rA | KPQVDRY |
| 534 | 3.05E-011 | 1b3rA | KRATDVM |
| 535 | 3.05E-011 | 1b3rA | LPKKLD |
| 536 | 3.05E-011 | 1b3rA | NIGHFD |
| 537 | 3.05E-011 | 1b3rA | SCNIFS |
| 538 | 3.05E-011 | 1b3rA | SFVMSNSF |
| 539 | 3.05E-011 | 1b3rA | TDVMIAGK |
| 540 | 3.05E-011 | 1b3rA | VTKSKFDN |
| 541 | 3.05E-011 | 1b3rA | VVAGYG |
| 542 | 4.93E-011 | 1v8zA | AKFMGK |
| 543 | 4.93E-011 | 1v8zA | RMKLLG |
| 544 | 4.93E-011 | 1v8zA | AETGAGQHG |
| 545 | 4.93E-011 | 1v8zA | CVGGGSN |
| 546 | 4.93E-011 | 1v8zA | EGIIPALE |
| 547 | 4.93E-011 | 1v8zA | ERQKMNVFRM |
| 548 | 4.93E-011 | 1v8zA | GGGSNAMG |
| 549 | 4.93E-011 | 1v8zA | GLDYPG |
| 550 | 4.93E-011 | 1v8zA | GRPTPL |
| 551 | 4.93E-011 | 1v8zA | GSRTLKDA |
| 552 | 4.93E-011 | 1v8zA | HGGAHK |
| 553 | 4.93E-011 | 1v8zA | IGSVVGPHP |
| 554 | 4.93E-011 | 1v8zA | NVFRMKL |
| 555 | 4.93E-011 | 1v8zA | QILEAEG |
| 556 | 4.93E-011 | 1v8zA | RMKLLGA |
| 557 | 4.93E-011 | 1v8zA | SGRGDKD |
| 558 | 4.93E-011 | 1v8zA | TLKDAIN |
| 559 | 4.93E-011 | 1v8zA | VGGGSNA |
| 560 | 7.68E-011 | 1ffyA | LHDGP |
| 561 | 7.68E-011 | 1ffyA | KKMSKS |
| 562 | 7.68E-011 | 1ffyA | KMSKSL |
| 563 | 7.68E-011 | 1ffyA | LAEAEIE |
| 564 | 7.68E-011 | 1ffyA | LHMGH |
| 565 | 7.68E-011 | 1ffyA | DGPPYANG |
| 566 | 7.68E-011 | 1ffyA | DVWFDSG |
| 567 | 7.68E-011 | 1ffyA | DWRTKKP |
| 568 | 7.68E-011 | 1ffyA | EGSDQYRGWFNSS |
| 569 | 7.68E-011 | 1ffyA | GDHVTTD |
| 570 | 7.68E-011 | 1ffyA | GHALNKI |
| 571 | 7.68E-011 | 1ffyA | HVTTDAGTGCVHTAPGHGEDD |
| 572 | 7.68E-011 | 1ffyA | ISRQRVWG |
| 573 | 7.68E-011 | 1ffyA | KDFIVRYKTMQGFYAPYVPGWDTHGLPIEQALTKKGVDRKKMS |
| 574 | 7.68E-011 | 1ffyA | LAEAEIEY |
| 575 | 7.68E-011 | 1ffyA | LLEVDRYLLNRLREFTASTINNYENFDYLNIYQEVQNFINVELSNFYLDYGKDILYIE |
| 576 | 7.68E-011 | 1ffyA | NMVRDRG |
| 577 | 7.68E-011 | 1ffyA | PYKFLLSHGFVMDGEGKKMSKSLGNVIVPDQVVKQKGADIARLWVSSTDYL |
| 578 | 7.68E-011 | 1ffyA | TFMNLRDDVNRALE |
| 579 | 7.68E-011 | 1ffyA | TLLMPKTDFPMRGGLPNKEPQIQE |
| 580 | 7.68E-011 | 1ffyA | WGKTRIYNM |
| 581 | 7.68E-011 | 1ffyA | WTTTPWT |
| 582 | 7.68E-011 | 1ffyA | YRKIRNT |
| 583 | 1.40E-010 | 1ltkA | DAFGTA |
| 584 | 1.40E-010 | 1ltkA | IGGGMA |
| 585 | 1.40E-010 | 1ltkA | KANKEDVE |
| 586 | 1.40E-010 | 1ltkA | LGGAKV |
| 587 | 1.40E-010 | 1ltkA | STGGGA |
| 588 | 1.40E-010 | 1ltkA | AILGGAK |
| 589 | 1.40E-010 | 1ltkA | DAFGTAHR |
| 590 | 1.40E-010 | 1ltkA | DKIQLIKN |
| 591 | 1.40E-010 | 1ltkA | DVFINDAF |
| 592 | 1.40E-010 | 1ltkA | EEEGKG |
| 593 | 1.40E-010 | 1ltkA | EKAKAKNV |
| 594 | 1.40E-010 | 1ltkA | GGAKVSDK |
| 595 | 1.40E-010 | 1ltkA | GNKVKA |
| 596 | 1.40E-010 | 1ltkA | HRAHSS |
| 597 | 1.40E-010 | 1ltkA | ILGGAKV |
| 598 | 1.40E-010 | 1ltkA | KVLNNM |
| 599 | 1.40E-010 | 1ltkA | LMKKEL |
| 600 | 1.40E-010 | 1ltkA | MAYTF |
| 601 | 1.40E-010 | 1ltkA | NDAFGT |
| 602 | 1.40E-010 | 1ltkA | RAHSSMVG |
| 603 | 1.40E-010 | 1ltkA | RVDFNVP |
| 604 | 1.40E-010 | 1ltkA | SHVSTG |
| 605 | 1.40E-010 | 1ltkA | SLFDEAG |
| 606 | 1.40E-010 | 1ltkA | STGGGASL |
| 607 | 1.70E-010 | 1tyfA | AQMLFLE |
| 608 | 1.70E-010 | 1tyfA | EYGLVD |
| 609 | 1.70E-010 | 1tyfA | IYSRLL |
| 610 | 1.70E-010 | 1tyfA | VAQMLFL |
| 611 | 1.70E-010 | 1tyfA | AQMLFLEAE |
| 612 | 1.70E-010 | 1tyfA | ATDIEI |
| 613 | 1.70E-010 | 1tyfA | DIEIHA |
| 614 | 1.70E-010 | 1tyfA | DIYSRLL |
| 615 | 1.70E-010 | 1tyfA | EAVEYGL |
| 616 | 1.70E-010 | 1tyfA | ERVIFL |
| 617 | 1.70E-010 | 1tyfA | FLEAENP |
| 618 | 1.70E-010 | 1tyfA | GAKGKR |
| 619 | 1.70E-010 | 1tyfA | GQATDI |
| 620 | 1.70E-010 | 1tyfA | IYDTMQ |
| 621 | 1.70E-010 | 1tyfA | LFLEAEN |
| 622 | 1.70E-010 | 1tyfA | MANLIVAQ |
| 623 | 1.70E-010 | 1tyfA | MNELMA |
| 624 | 1.70E-010 | 1tyfA | SIYDTM |
| 625 | 1.70E-010 | 1tyfA | TGQSLE |
| 626 | 1.70E-010 | 1tyfA | VEYGLVD |
| 627 | 1.70E-010 | 1tyfA | YDTMQFIKP |
| 628 | 3.03E-010 | 1xdoA | PRRRKP |
| 629 | 3.03E-010 | 1xdoA | DWMTR |
| 630 | 3.03E-010 | 1xdoA | ELQARFD |
| 631 | 3.03E-010 | 1xdoA | FDEEAN |
| 632 | 3.03E-010 | 1xdoA | RDAEYD |
| 633 | 3.03E-010 | 1xdoA | VRFAELK |
| 634 | 3.03E-010 | 1xdoA | APGLKIH |
| 635 | 3.03E-010 | 1xdoA | ARFDEE |
| 636 | 3.03E-010 | 1xdoA | FKGFFDYD |
| 637 | 3.03E-010 | 1xdoA | IKINIYRVAKDS |
| 638 | 3.03E-010 | 1xdoA | ISIVDRYLEHDR |
| 639 | 3.03E-010 | 1xdoA | KELSWLSFNERVLQEAADK |
| 640 | 3.03E-010 | 1xdoA | LEHDRVY |
| 641 | 3.03E-010 | 1xdoA | LLYYPYHTF |
| 642 | 3.03E-010 | 1xdoA | LRQHITPILIN |
| 643 | 3.03E-010 | 1xdoA | LVQFLKDDYTYLAVEIIRG |
| 644 | 3.03E-010 | 1xdoA | MIHAAHN |
| 645 | 3.03E-010 | 1xdoA | NNLVDKG |
| 646 | 3.03E-010 | 1xdoA | RGMCSL |
| 647 | 3.03E-010 | 1xdoA | RYAHIGTGNF |
| 648 | 3.03E-010 | 1xdoA | SNNLDEFYKVRF |
| 649 | 1.84E-009 | 1h4lA | DFGLAR |
| 650 | 1.84E-009 | 1h4lA | EIVALK |
| 651 | 1.84E-009 | 1h4lA | WSAGC |
| 652 | 1.84E-009 | 1h4lA | ADFGLA |
| 653 | 1.84E-009 | 1h4lA | HRDLKP |
| 654 | 1.84E-009 | 1h4lA | KLADFGL |
| 655 | 1.84E-009 | 1h4lA | DDEGVPSSALREICLLKELKHKNIVRL |
| 656 | 1.84E-009 | 1h4lA | DFGLARAFG |
| 657 | 1.84E-009 | 1h4lA | DQDLKKY |
| 658 | 1.84E-009 | 1h4lA | EIVALKR |
| 659 | 1.84E-009 | 1h4lA | GCIFAE |
| 660 | 1.84E-009 | 1h4lA | GELKLADFGLA |
| 661 | 1.84E-009 | 1h4lA | GRPLFPG |
| 662 | 1.84E-009 | 1h4lA | KLTLVFE |
| 663 | 1.84E-009 | 1h4lA | KPQNLL |
| 664 | 1.84E-009 | 1h4lA | LHRDLKP |
| 665 | 1.84E-009 | 1h4lA | LVFEFCD |
| 666 | 1.84E-009 | 1h4lA | VVTLWYR |
| 667 | 1.84E-009 | 1h4lA | WSAGCI |
| 668 | 1.84E-009 | 1tzlA | IHRDAF |
| 669 | 1.84E-009 | 1tzlA | DPEPQVT |
| 670 | 1.84E-009 | 1tzlA | EAEDMMTDMCVMSAKIGGFLPGS |
| 671 | 1.84E-009 | 1tzlA | HKKNTVEYQKNIDKFVNVIQGQLM |
| 672 | 1.84E-009 | 1tzlA | IGEIDSGLKIG |
| 673 | 1.84E-009 | 1tzlA | LVFCQTVMSTELI |
| 674 | 1.84E-009 | 1tzlA | QIHRDAFSYGAV |
| 675 | 1.84E-009 | 1tzlA | QRPLLVK |
| 676 | 1.84E-009 | 1tzlA | RFNLFPAVAC |
| 677 | 1.84E-009 | 1tzlA | VGGMSTHWTC |
| 678 | 2.51E-009 | 1k75A | IPGGSA |
| 679 | 2.51E-009 | 1k75A | PGGSAP |
| 680 | 2.51E-009 | 1k75A | AGPSEVL |
| 681 | 2.51E-009 | 1k75A | AHKNAV |
| 682 | 2.51E-009 | 1k75A | DLLSQAEH |
| 683 | 2.51E-009 | 1k75A | GDYASGTNH |
| 684 | 2.51E-009 | 1k75A | GGAQAIAA |
| 685 | 2.51E-009 | 1k75A | GPGNAFV |
| 686 | 2.51E-009 | 1k75A | IETFHTAQKLPPVDVETQ |
| 687 | 2.51E-009 | 1k75A | KIFGPGN |
| 688 | 2.51E-009 | 1k75A | LPTYGY |
| 689 | 2.51E-009 | 1k75A | NHVLPT |
| 690 | 2.51E-009 | 1k75A | PKVDKI |
| 691 | 2.51E-009 | 1k75A | QAEHG |
| 692 | 2.51E-009 | 1k75A | QQRQLLMRPAISASESITRTVNDILD |
| 693 | 2.51E-009 | 1k75A | SSLGLADF |
| 694 | 2.51E-009 | 1k75A | STVLMLA |
| 695 | 2.51E-009 | 1k75A | VAEAVERQLAELPRA |
| 696 | 2.51E-009 | 1k75A | VTEAKRQV |
| 697 | 5.04E-009 | 1fugA | GGAFSG |
| 698 | 5.04E-009 | 1fugA | RHGGGA |
| 699 | 5.04E-009 | 1fugA | RKIIVD |
| 700 | 5.04E-009 | 1fugA | SYAIGV |
| 701 | 5.04E-009 | 1fugA | VGGEITT |
| 702 | 5.04E-009 | 1fugA | ARVACE |
| 703 | 5.04E-009 | 1fugA | FSGKDP |
| 704 | 5.04E-009 | 1fugA | FVIGGP |
| 705 | 5.04E-009 | 1fugA | GAGDQG |
| 706 | 5.04E-009 | 1fugA | GEITTS |
| 707 | 5.04E-009 | 1fugA | GGPMGD |
| 708 | 5.04E-009 | 1fugA | GLTGRKI |
| 709 | 5.04E-009 | 1fugA | HGGGAFSGKD |
| 710 | 5.04E-009 | 1fugA | IGKQSPDI |
| 711 | 5.04E-009 | 1fugA | LRPDAKSQV |
| 712 | 5.04E-009 | 1fugA | NPTGRF |
| 713 | 5.04E-009 | 1fugA | QSPDINQGVDR |
| 714 | 5.04E-009 | 1fugA | SGKDPSK |
| 715 | 5.04E-009 | 1fugA | SYAIGVA |
| 716 | 5.04E-009 | 1fugA | VACET |
| 717 | 5.04E-009 | 1fugA | YAIGVAEP |
| 718 | 1.00E-008 | 1ck7A | YSLFLV |
| 719 | 1.00E-008 | 1ck7A | WCATT |
| 720 | 1.00E-008 | 1ck7A | KWGFCPD |
| 721 | 1.00E-008 | 1ck7A | KYGFCP |
| 722 | 1.00E-008 | 1ck7A | LFLVAAHE |
| 723 | 1.00E-008 | 1ck7A | CNLFVLKDTLKKMQKFFGLP |
| 724 | 1.00E-008 | 1ck7A | GGDSHFDDDELWTLGEGQVVRVKYGNADGEYCKFPF |
| 725 | 1.00E-008 | 1ck7A | KPRCGNPDVANYNFFPRKPKW |
| 726 | 1.00E-008 | 1ck7A | LPEKIDAVYE |
| 727 | 1.00E-008 | 1ck7A | NKKTYIF |
| 728 | 1.00E-008 | 1ck7A | YDDDRKWGFCPDQGYSLFLVAAHEFGHAMGLEHS |
| 729 | 1.00E-008 | 1oqsA | CECDR |
| 730 | 1.00E-008 | 1oqsA | CYCGWGG |
| 731 | 1.00E-008 | 1oqsA | DATDRCC |
| 732 | 1.00E-008 | 1oqsA | GCYCG |
| 733 | 1.00E-008 | 1oqsA | HDCCY |
| 734 | 1.00E-008 | 1oqsA | NGDIVCG |
| 735 | 1.00E-008 | 1oqsA | VCECD |
| 736 | 1.00E-008 | 1oqsA | YGCYC |
| 737 | 2.11E-008 | 1crwG | AAQNII |
| 738 | 2.11E-008 | 1crwG | GFGRIG |
| 739 | 2.11E-008 | 1crwG | ASCTTN |
| 740 | 2.11E-008 | 1crwG | ESTGVF |
| 741 | 2.11E-008 | 1crwG | GFGRIGRLV |
| 742 | 2.11E-008 | 1crwG | INGFGR |
| 743 | 2.11E-008 | 1crwG | KDWRGGR |
| 744 | 2.11E-008 | 1crwG | NIIPSSTGA |
| 745 | 2.11E-008 | 1crwG | RVPTPNVS |
| 746 | 2.11E-008 | 1crwG | SNASCTT |
| 747 | 2.11E-008 | 1crwG | STGAAK |
| 748 | 2.11E-008 | 1crwG | AAQNIIP |
| 749 | 2.11E-008 | 1crwG | APSADAPM |
| 750 | 2.11E-008 | 1crwG | AVNDPFI |
| 751 | 2.11E-008 | 1crwG | ESTGVFT |
| 752 | 2.11E-008 | 1crwG | GPSAKDWRGGR |
| 753 | 2.11E-008 | 1crwG | GRGAAQN |
| 754 | 2.11E-008 | 1crwG | INGFGRIGRLVLR |
| 755 | 2.11E-008 | 1crwG | KDWRGGRG |
| 756 | 2.11E-008 | 1crwG | KIGINGFGRIGRLV |
| 757 | 2.11E-008 | 1crwG | KTVDGPS |
| 758 | 2.11E-008 | 1crwG | SWYDNEFGYS |
| 759 | 2.11E-008 | 1crwG | TATQKTV |
| 760 | 2.11E-008 | 1crwG | TGAAKAVG |
| 761 | 2.11E-008 | 1crwG | VIISAPS |
| 762 | 3.29E-008 | 1p8jA | IYSASWGP |
| 763 | 3.29E-008 | 1p8jA | YSEACS |
| 764 | 3.29E-008 | 1p8jA | GNVPWYSEACSSTLATTYSSGNQNEKQIVTTDLRQKCT |
| 765 | 3.29E-008 | 1p8jA | IGGVRMLDGEVTDAVEARSLGLNPNHIHIYSASWGPEDDGKTVDGPA |
| 766 | 3.29E-008 | 1p8jA | LVLYGTA |
| 767 | 3.29E-008 | 1p8jA | QGRGGLGSI |
| 768 | 3.29E-008 | 1p8jA | TNGVGRKVSHSYGYGLLDAGAMVA |
| 769 | 3.29E-008 | 1p8jA | VWASGNGGREHDSCNCDGYTNSIYTLSISS |
| 770 | 4.52E-008 | 1m8tA | AAICFA |
| 771 | 4.52E-008 | 1m8tA | APYNKEN |
| 772 | 4.52E-008 | 1m8tA | CCQVHD |
| 773 | 4.52E-008 | 1m8tA | CDRVAA |
| 774 | 4.52E-008 | 1m8tA | CTIPGS |
| 775 | 4.52E-008 | 1m8tA | DELDRC |
| 776 | 4.52E-008 | 1m8tA | DNDEC |
| 777 | 4.52E-008 | 1m8tA | GGSGTP |
| 778 | 4.52E-008 | 1m8tA | GSGTPVD |
| 779 | 4.52E-008 | 1m8tA | HDNCY |
| 780 | 4.52E-008 | 1m8tA | LDRCCQ |
| 781 | 4.52E-008 | 1m8tA | VCDCDR |
| 782 | 9.26E-008 | 1kywA | AHNPG |
| 783 | 9.26E-008 | 1kywA | GMTAFE |
| 784 | 9.26E-008 | 1kywA | STITMK |
| 785 | 9.26E-008 | 1kywA | AFEYHG |
| 786 | 9.26E-008 | 1kywA | AMQLAS |
| 787 | 9.26E-008 | 1kywA | APVMLDR |
| 788 | 9.26E-008 | 1kywA | DLPHVI |
| 789 | 9.26E-008 | 1kywA | GGIPFNKAYGM |
| 790 | 9.26E-008 | 1kywA | GGKERT |
| 791 | 9.26E-008 | 1kywA | GMSDHS |
| 792 | 9.26E-008 | 1kywA | HDWSD |
| 793 | 9.26E-008 | 1kywA | HVIEDAP |
| 794 | 9.26E-008 | 1kywA | LKDAVL |
| 795 | 9.26E-008 | 1kywA | RFNKVFN |
| 796 | 9.26E-008 | 1kywA | VDVGGGTG |
| 797 | 9.26E-008 | 1kywA | VGGDMF |
| 798 | 9.26E-008 | 1kywA | DVIMLAHNPGGKERT |
| 799 | 9.26E-008 | 1kywA | ECILPVAPD |
| 800 | 1.30E-007 | 1j0xO | GFGRIG |
| 801 | 1.30E-007 | 1j0xO | GMAFRV |
| 802 | 1.30E-007 | 1j0xO | VIISAP |
| 803 | 1.30E-007 | 1j0xO | ASCTTN |
| 804 | 1.30E-007 | 1j0xO | ESTGVF |
| 805 | 1.30E-007 | 1j0xO | GFGRIGRLV |
| 806 | 1.30E-007 | 1j0xO | RVPTPNVS |
| 807 | 1.30E-007 | 1j0xO | STGAAK |
| 808 | 1.30E-007 | 1j0xO | SWYDNE |
| 809 | 1.30E-007 | 1j0xO | AAQNIIP |
| 810 | 1.30E-007 | 1j0xO | ESTGVFT |
| 811 | 1.30E-007 | 1j0xO | FDAGAGI |
| 812 | 1.30E-007 | 1j0xO | IVSNASCTTNCLAP |
| 813 | 1.30E-007 | 1j0xO | KTVDGPS |
| 814 | 1.30E-007 | 1j0xO | PSGKLWR |
| 815 | 1.30E-007 | 1j0xO | SNASCTTNCLAPL |
| 816 | 1.30E-007 | 1j0xO | SWYDNEFGYS |
| 817 | 1.30E-007 | 1j0xO | TATQKTV |
| 818 | 1.30E-007 | 1j0xO | VAINDPFI |
| 819 | 1.30E-007 | 1j0xO | VIISAPS |
| 820 | 1.30E-007 | 1j0xO | VKLISWYDNE |
| 821 | 1.30E-007 | 1j0xO | WRDGRGA |
| 822 | 2.25E-007 | 1x23A | FPTDYP |
| 823 | 2.25E-007 | 1x23A | CSAGPVG |
| 824 | 2.25E-007 | 1x23A | DPNPDDPLVP |
| 825 | 2.25E-007 | 1x23A | FHWQATIMGP |
| 826 | 2.25E-007 | 1x23A | GGVFFL |
| 827 | 2.25E-007 | 1x23A | ICLDIL |
| 828 | 2.25E-007 | 1x23A | PLVPEIA |
| 829 | 2.25E-007 | 1x23A | QWSPALTISKVLLSICSLL |
| 830 | 2.25E-007 | 1x23A | YPFKPP |
| 831 | 9.23E-007 | 1b6cB | DIYAMG |
| 832 | 9.23E-007 | 1b6cB | MRECW |
| 833 | 9.23E-007 | 1b6cB | GEEVAVK |
| 834 | 9.23E-007 | 1b6cB | GSGSGLP |
| 835 | 9.23E-007 | 1b6cB | IADLGLA |
| 836 | 9.23E-007 | 1b6cB | NILGFIA |
| 837 | 9.23E-007 | 1b6cB | RPNIPNRW |
| 838 | 9.23E-007 | 1b6cB | RVGTKRYM |
| 839 | 9.23E-007 | 1b6cB | VAVKIF |
| 840 | 9.23E-007 | 1b6cB | VPSDPS |
| 841 | 1.20E-006 | 1dc3A | GFGRIG |
| 842 | 1.20E-006 | 1dc3A | ASCTTN |
| 843 | 1.20E-006 | 1dc3A | INGFGR |
| 844 | 1.20E-006 | 1dc3A | NIIPSSTGA |
| 845 | 1.20E-006 | 1dc3A | SNASCTT |
| 846 | 1.20E-006 | 1dc3A | STGAAK |
| 847 | 1.20E-006 | 1dc3A | SWYDNE |
| 848 | 1.20E-006 | 1dc3A | AYMLKYDS |
| 849 | 1.20E-006 | 1dc3A | ERDPAN |
| 850 | 1.20E-006 | 1dc3A | GINGFGRIGRIV |
| 851 | 1.20E-006 | 1dc3A | IVSNASCTTNCLAP |
| 852 | 1.20E-006 | 1dc3A | KLVSWYDNE |
| 853 | 1.20E-006 | 1dc3A | SNASCTTNCLAPL |
| 854 | 1.20E-006 | 1dc3A | TATQKTV |
| 855 | 1.20E-006 | 1dc3A | TGAAKAVG |
| 856 | 1.20E-006 | 1dc3A | TGPSKD |
| 857 | 1.20E-006 | 1dc3A | VGINGFGRIGR |
| 858 | 1.20E-006 | 1dc3A | YDSTHG |
| 859 | 1.31E-006 | 1fjmA | DLLRLF |
| 860 | 1.31E-006 | 1fjmA | LLWSDP |
| 861 | 1.31E-006 | 1fjmA | SDPDKD |
| 862 | 1.31E-006 | 1fjmA | FYDEC |
| 863 | 1.31E-006 | 1fjmA | GYEFFA |
| 864 | 1.31E-006 | 1fjmA | RPTDVP |
| 865 | 1.31E-006 | 1fjmA | CKRRYNI |
| 866 | 1.31E-006 | 1fjmA | DECKRR |
| 867 | 1.31E-006 | 1fjmA | DLDLICR |
| 868 | 1.31E-006 | 1fjmA | DLLRLFEYGG |
| 869 | 1.31E-006 | 1fjmA | DLLWSDP |
| 870 | 1.31E-006 | 1fjmA | DVPDQG |
| 871 | 1.31E-006 | 1fjmA | GAMMSV |
| 872 | 1.31E-006 | 1fjmA | GLLCDL |
| 873 | 1.31E-006 | 1fjmA | NDRGVS |
| 874 | 1.31E-006 | 1fjmA | RPTDVPD |
| 875 | 1.31E-006 | 1fjmA | VVEDGYEFF |
| 876 | 1.40E-006 | 2h12A | HEQNA |
| 877 | 1.40E-006 | 2h12A | LYPNVD |
| 878 | 1.40E-006 | 2h12A | GFGHRVYK |
| 879 | 1.40E-006 | 2h12A | HGGANE |
| 880 | 1.40E-006 | 2h12A | DAHPMA |
| 881 | 1.40E-006 | 2h12A | GVFTFDPG |
| 882 | 1.40E-006 | 2h12A | HGGANEA |
| 883 | 1.40E-006 | 2h12A | LHADHEQNASTSTVR |
| 884 | 1.40E-006 | 2h12A | LWGPAHGGANE |
| 885 | 1.40E-006 | 2h12A | PNVDFYS |
| 886 | 1.40E-006 | 2h12A | RPRQLY |
| 887 | 1.40E-006 | 2h12A | VNPVLARAM |
| 888 | 1.93E-006 | 1jn0A | VVGVNE |
| 889 | 1.93E-006 | 1jn0A | APGKGDIPT |
| 890 | 1.93E-006 | 1jn0A | KVLITAP |
| 891 | 1.93E-006 | 1jn0A | SNASCTT |
| 892 | 1.93E-006 | 1jn0A | TGAAKAVALV |
| 893 | 1.93E-006 | 1jn0A | HLLKYDSILGTF |
| 894 | 1.93E-006 | 1jn0A | KGTMTTTHSYTGDQRLLDASHRDLRRARAA |
| 895 | 1.98E-006 | 1i1iP | ATPGTNMPATFGHLAGGYD |
| 896 | 1.98E-006 | 1i1iP | FYLDLYPREGKY |
| 897 | 1.98E-006 | 1i1iP | HAACFGLQPGCL |
| 898 | 1.98E-006 | 1i1iP | LCIDFNKNLNED |
| 899 | 1.98E-006 | 1i1iP | LDFPQHVS |
| 900 | 1.98E-006 | 1i1iP | QYYGYLWSEV |
| 901 | 1.98E-006 | 1i1iP | TLKYPHYFP |
| 902 | 1.98E-006 | 1i1iP | TYFHEFGHVMHQ |
| 903 | 1.98E-006 | 1i1iP | AGRPSLLRHDEVRTYFHEFGHVMHQICAQTDFARFSGTNVETDFVEVPSQMLENWVWD |
| 904 | 1.98E-006 | 1i1iP | LLEKLVASRLVNTGLLTLRQIVLSKVDQSLHTN |
| 905 | 1.98E-006 | 1i1iP | THADFVLE |
| 906 | 1.98E-006 | 1i1iP | TLKYPHYFPVMKKCC |
| 907 | 1.99E-006 | 1t09A | GTVTRH |
| 908 | 1.99E-006 | 1t09A | NYDGDV |
| 909 | 1.99E-006 | 1t09A | CKNIPRLV |
| 910 | 1.99E-006 | 1t09A | EAAHGTVTRH |
| 911 | 1.99E-006 | 1t09A | GDQYRAT |
| 912 | 1.99E-006 | 1t09A | KNYDGDVQSD |
| 913 | 1.99E-006 | 1t09A | KPIIIGRHA |
| 914 | 1.99E-006 | 1t09A | MTSVLVCPDGKT |
| 915 | 1.99E-006 | 1t09A | SPNGTIRNIL |
| 916 | 1.99E-006 | 1t09A | TKNTILK |
| 917 | 1.99E-006 | 1t09A | TSTNPIASIFAW |
| 918 | 1.99E-006 | 1t09A | VAQGYGSLG |
| 919 | 1.99E-006 | 1t09A | VKCATITPDE |
| 920 | 2.02E-006 | 1a0dA | DIAPEG |
| 921 | 2.02E-006 | 1a0dA | ELDNLARF |
| 922 | 2.02E-006 | 1a0dA | EPTKHQYD |
| 923 | 2.02E-006 | 1a0dA | GHTFEHEL |
| 924 | 2.02E-006 | 1a0dA | GWDTDEF |
| 925 | 2.02E-006 | 1a0dA | HGAATS |
| 926 | 2.02E-006 | 1a0dA | HQYDFD |
| 927 | 2.02E-006 | 1a0dA | LGSVDANQG |
| 928 | 2.02E-006 | 1a0dA | LLGWDTD |
| 929 | 2.02E-006 | 1a0dA | NFDAKVRR |
| 930 | 2.02E-006 | 1a0dA | NIEANHATL |
| 931 | 2.02E-006 | 1a0dA | TTLAMYEIL |
| 932 | 2.02E-006 | 1a0dA | WGGREG |
| 933 | 2.02E-006 | 1a0dA | YWHTF |
| 934 | 2.06E-006 | 1wddA | AAESSTGT |
| 935 | 2.06E-006 | 1wddA | AGVKDY |
| 936 | 2.06E-006 | 1wddA | GNVFGFKA |
| 937 | 2.06E-006 | 1wddA | HRAMHA |
| 938 | 2.06E-006 | 1wddA | KPKLGLS |
| 939 | 2.06E-006 | 1wddA | LLHIHRAMH |
| 940 | 2.06E-006 | 1wddA | LRGGLDFTKDDEN |
| 941 | 2.06E-006 | 1wddA | TWTTVWTD |
| 942 | 2.06E-006 | 1wddA | VASGGIH |
| 943 | 2.06E-006 | 1wddA | VGKLEG |
| 944 | 2.06E-006 | 1wddA | VLQFGGGT |
| 945 | 2.06E-006 | 1wddA | WKAIKFEFEPVD |
| 946 | 2.84E-006 | 1y8gA | GSLPFD |
| 947 | 2.84E-006 | 1y8gA | LYLVME |
| 948 | 2.84E-006 | 1y8gA | VHRDLK |
| 949 | 2.84E-006 | 1y8gA | GEVFDYLV |
| 950 | 2.84E-006 | 1y8gA | GSPPYA |
| 951 | 2.84E-006 | 1y8gA | IVSAVQYCHQK |
| 952 | 2.84E-006 | 1y8gA | LNHPNIVKLFEVIETEKTLYL |
| 953 | 2.84E-006 | 1y8gA | SLQKLFREVRIMK |
| 954 | 2.84E-006 | 1y8gA | TFCGS |
| 955 | 2.87E-006 | 1v6aA | GMVGMA |
| 956 | 2.87E-006 | 1v6aA | LDSARF |
| 957 | 2.87E-006 | 1v6aA | GEHGDS |
| 958 | 2.87E-006 | 1v6aA | LDSARFR |
| 959 | 2.87E-006 | 1v6aA | SILLKD |
| 960 | 2.87E-006 | 1v6aA | VSNPVD |
| 961 | 2.87E-006 | 1v6aA | AYEVIKLKG |
| 962 | 2.87E-006 | 1v6aA | GEHGDSSV |
| 963 | 2.87E-006 | 1v6aA | KGYTSW |
| 964 | 2.87E-006 | 1v6aA | KVTVVGVG |
| 965 | 2.87E-006 | 1v6aA | LVVSNPVD |
| 966 | 2.87E-006 | 1v6aA | NLDSARFR |
| 967 | 2.87E-006 | 1v6aA | NPVDIL |
| 968 | 2.87E-006 | 1v6aA | SILLKDL |
| 969 | 2.87E-006 | 1v6aA | SNPVDI |
| 970 | 2.87E-006 | 1v6aA | TAGARQ |
| 971 | 2.87E-006 | 1v6aA | VVDSAYE |
| 972 | 3.37E-006 | 1cw3A | ELGGKS |
| 973 | 3.37E-006 | 1cw3A | LELGGK |
| 974 | 3.37E-006 | 1cw3A | TPLTAL |
| 975 | 3.37E-006 | 1cw3A | GGKSPNI |
| 976 | 3.37E-006 | 1cw3A | TLELGGK |
| 977 | 3.37E-006 | 1cw3A | YTEVKTVT |
| 978 | 3.37E-006 | 1cw3A | ISYLVDLDMVLKCLRYYAGWADKYHGKTIPIDGD |
| 979 | 3.37E-006 | 1cw3A | YGLAAAVFTKDLDKANYLSQALQAGTVW |
| 980 | 4.76E-006 | 1bxsA | GGFKMSG |
| 981 | 4.76E-006 | 1bxsA | PFGGFK |
| 982 | 4.76E-006 | 1bxsA | QGQCC |
| 983 | 4.76E-006 | 1bxsA | TPLTAL |
| 984 | 4.76E-006 | 1bxsA | AGWADK |
| 985 | 4.76E-006 | 1bxsA | GWADKI |
| 986 | 4.76E-006 | 1bxsA | LADLIERD |
| 987 | 4.76E-006 | 1bxsA | LLNKLADL |
| 988 | 4.76E-006 | 1bxsA | PFGGFKMSGNGRE |
| 989 | 4.76E-006 | 1bxsA | FYHQGQCC |
| 990 | 6.13E-006 | 1sc6B | DVVSLHVPE |
| 991 | 6.13E-006 | 1sc6B | EAQENIG |
| 992 | 6.13E-006 | 1sc6B | GAAIDVFP |
| 993 | 6.13E-006 | 1sc6B | GCFCIGTNQVDL |
| 994 | 6.13E-006 | 1sc6B | HRGVWNK |
| 995 | 6.13E-006 | 1sc6B | KYSDNGSTLS |
| 996 | 6.13E-006 | 1sc6B | RGIPVFNAPFSNTRSVAELV |
| 997 | 6.13E-006 | 1sc6B | RGTVVDI |
| 998 | 6.13E-006 | 1sc6B | VYFYDIENKLPLGNA |
| 999 | 6.40E-006 | 1qwlA | ERIPER |
| 1000 | 6.40E-006 | 1qwlA | YMQNG |
| 1001 | 6.40E-006 | 1qwlA | ANVVPGI |
| 1002 | 6.40E-006 | 1qwlA | DAIKFPD |
| 1003 | 6.40E-006 | 1qwlA | FAEVEQ |
| 1004 | 6.40E-006 | 1qwlA | FFIRDA |
| 1005 | 6.40E-006 | 1qwlA | GRLFSY |
| 1006 | 6.40E-006 | 1qwlA | KFPDFIH |
| 1007 | 6.40E-006 | 1qwlA | NPENYF |
| 1008 | 6.40E-006 | 1qwlA | PERVVHAKG |
| 1009 | 6.40E-006 | 1qwlA | PVFFIRD |
| 1010 | 6.40E-006 | 1qwlA | RERIPERVVHA |
| 1011 | 6.40E-006 | 1qwlA | RFSTVAG |
| 1012 | 6.40E-006 | 1qwlA | RYRLGVN |
| 1013 | 6.40E-006 | 1qwlA | WFLEKLA |
| 1014 | 6.47E-006 | 1yqzA | AHRAAS |
| 1015 | 6.47E-006 | 1yqzA | FEKDRDMSFANCALPY |
| 1016 | 6.47E-006 | 1yqzA | IIEGVGTHPNS |
| 1017 | 6.47E-006 | 1yqzA | KQITVKTYHEVI |
| 1018 | 6.47E-006 | 1yqzA | LGNNIVKFFDYT |
| 1019 | 6.47E-006 | 1yqzA | LILSPGASAN |
| 1020 | 6.47E-006 | 1yqzA | LTEFEVAYAPPYSHPKDLIN |
| 1021 | 6.47E-006 | 1yqzA | RNLEDT |
| 1022 | 6.47E-006 | 1yqzA | SLEVLENL |
| 1023 | 6.47E-006 | 1yqzA | VGAVAGGATCASQIRRLDKES |
| 1024 | 6.47E-006 | 1yqzA | VPLAWGAHR |
| 1025 | 6.70E-006 | 1bhnA | GRNIIH |
| 1026 | 6.70E-006 | 1bhnA | GTIRGD |
| 1027 | 6.70E-006 | 1bhnA | HGSDSV |
| 1028 | 6.70E-006 | 1bhnA | IIKRFEQKGFRLVAMKF |
| 1029 | 6.70E-006 | 1bhnA | NIIHGSD |
| 1030 | 6.70E-006 | 1bhnA | TIRGDF |
| 1031 | 7.43E-006 | 1cqeA | CDCTRTG |
| 1032 | 7.43E-006 | 1cqeA | FNEYRKRF |
| 1033 | 7.43E-006 | 1cqeA | FSNVSYYTR |
| 1034 | 7.43E-006 | 1cqeA | FVNATFIR |
| 1035 | 7.43E-006 | 1cqeA | GHGVDL |
| 1036 | 7.43E-006 | 1cqeA | LYHWHPL |
| 1037 | 7.43E-006 | 1cqeA | WLREHNRVCD |
| 1038 | 7.43E-006 | 1cqeA | YSYEQFLFNTSMLVDYGVEALVDAFSRQ |
| 1039 | 7.83E-006 | 2a7rA | KVGIGPGS |
| 1040 | 7.83E-006 | 2a7rA | DVANGYSEHFV |
| 1041 | 7.83E-006 | 2a7rA | FYGMSS |
| 1042 | 7.83E-006 | 2a7rA | GADFVMLGGM |
| 1043 | 7.83E-006 | 2a7rA | GGVAEYRA |
| 1044 | 7.83E-006 | 2a7rA | GSVCTTR |
| 1045 | 7.83E-006 | 2a7rA | IAANMD |
| 1046 | 7.83E-006 | 2a7rA | ICLDVANGYSE |
| 1047 | 7.83E-006 | 2a7rA | KLDFKDVLLRPKRS |
| 1048 | 7.83E-006 | 2a7rA | NMDTVGTF |
| 1049 | 7.83E-006 | 2a7rA | RPKRSTLKSRS |
| 1050 | 7.83E-006 | 2a7rA | YRASEGK |
| 1051 | 8.09E-006 | 1a0cA | DIAPEG |
| 1052 | 8.09E-006 | 1a0cA | EPTKHQYD |
| 1053 | 8.09E-006 | 1a0cA | FTADGTD |
| 1054 | 8.09E-006 | 1a0cA | GWDTDQFP |
| 1055 | 8.09E-006 | 1a0cA | LLGWDTD |
| 1056 | 8.09E-006 | 1a0cA | LWGTANLFS |
| 1057 | 8.09E-006 | 1a0cA | NFDAKVRR |
| 1058 | 8.09E-006 | 1a0cA | NIEANHATL |
| 1059 | 8.09E-006 | 1a0cA | SGRQELLE |
| 1060 | 8.09E-006 | 1a0cA | TMQRPW |
| 1061 | 8.09E-006 | 1a0cA | WGGREG |
| 1062 | 8.09E-006 | 1a0cA | YWHTF |
| 1063 | 8.27E-006 | 2aw5A | ASGSPF |
| 1064 | 8.27E-006 | 2aw5A | GIPVGKL |
| 1065 | 8.27E-006 | 2aw5A | PGQGNN |
| 1066 | 8.27E-006 | 2aw5A | RNEKLFY |
| 1067 | 8.27E-006 | 2aw5A | YTPTVG |
| 1068 | 8.27E-006 | 2aw5A | FASGSPFDPV |
| 1069 | 8.27E-006 | 2aw5A | FMPIVYTPTVGLACQ |
| 1070 | 8.27E-006 | 2aw5A | FQGAGEAA |
| 1071 | 8.27E-006 | 2aw5A | GMGIPVGKLALYTA |
| 1072 | 8.27E-006 | 2aw5A | LAALRIT |
| 1073 | 8.39E-006 | 1a49A | ANAVLD |
| 1074 | 8.39E-006 | 1a49A | IPAEKV |
| 1075 | 8.39E-006 | 1a49A | NAVLDG |
| 1076 | 8.39E-006 | 1a49A | DLKFGVEQ |
| 1077 | 8.39E-006 | 1a49A | DVANAVLDG |
| 1078 | 8.39E-006 | 1a49A | FDEILE |
| 1079 | 8.39E-006 | 1a49A | LKEMIKSGMN |
| 1080 | 8.39E-006 | 1a49A | NAVLDGAD |
| 1081 | 8.39E-006 | 1a49A | RPTRAE |
| 1082 | 8.39E-006 | 1a49A | SDVANA |
| 1083 | 8.39E-006 | 1a49A | VDLPAVS |
| 1084 | 1.51E-005 | 2g1iA | IAGSYAEH |
| 1085 | 1.51E-005 | 2g1iA | LHHTLG |
| 1086 | 1.51E-005 | 2g1iA | TTFGVGELSA |
| 1087 | 1.51E-005 | 2g1iA | GDGSLQLT |
| 1088 | 1.51E-005 | 2g1iA | ISQVLWGSIG |
| 1089 | 1.51E-005 | 2g1iA | LLLHHTLGNGD |
| 1090 | 1.51E-005 | 2g1iA | NNDGYTIER |
| 1091 | 1.51E-005 | 2g1iA | PGDFNL |
| 1092 | 1.51E-005 | 2g1iA | QRPVYLGLPANLVDL |
| 1093 | 1.51E-005 | 2g1iA | TETGTSAFGINQT |
| 1094 | 1.51E-005 | 2g1iA | YAEHVGVLH |
| 1095 | 1.63E-005 | 7mdhA | LYPLLR |
| 1096 | 1.63E-005 | 7mdhA | IWGNHS |
| 1097 | 1.63E-005 | 7mdhA | LEGVAMEL |
| 1098 | 1.63E-005 | 7mdhA | NAPDIP |
| 1099 | 1.63E-005 | 7mdhA | QGKALNAVA |
| 1100 | 1.63E-005 | 7mdhA | QLALKAGV |
| 1101 | 1.63E-005 | 7mdhA | VKVLVVGNP |
| 1102 | 1.63E-005 | 7mdhA | VVGNPCNTN |
| 1103 | 1.63E-005 | 7mdhA | MELEDSLYPLLREVSIGI |
| 1104 | 1.63E-005 | 7mdhA | VSIGIDPY |
| 1105 | 1.63E-005 | 7mdhA | VVGNPCNTNALICLKN |
| 1106 | 1.63E-005 | 7mdhA | WALLIGAKPRGPGMERA |
| 1107 | 1.64E-005 | 1gvhA | GPVGFM |
| 1108 | 1.64E-005 | 1gvhA | APAGDF |
| 1109 | 1.64E-005 | 1gvhA | CGPVGFMQ |
| 1110 | 1.64E-005 | 1gvhA | GDQREALFNAI |
| 1111 | 1.64E-005 | 1gvhA | GGQVSNWLH |
| 1112 | 1.64E-005 | 1gvhA | GVGQTPMLAMLDTLAK |
| 1113 | 1.64E-005 | 1gvhA | HTAQVNWFHAAENG |
| 1114 | 1.64E-005 | 1gvhA | IAQKHTS |
| 1115 | 1.64E-005 | 1gvhA | IFNMSNQ |
| 1116 | 1.64E-005 | 1gvhA | IKPEQY |
| 1117 | 1.64E-005 | 1gvhA | LISAGVG |
| 1118 | 1.64E-005 | 1gvhA | QEIRQYSL |
| 1119 | 1.64E-005 | 1gvhA | VAEYRPGQYLGVWLKPEGF |
| 1120 | 1.64E-005 | 1gvhA | YGVLANVFI |
| 1121 | 1.64E-005 | 1gvhA | YRIAVKRE |
| 1122 | 2.45E-005 | 1dbvO | GFGRIG |
| 1123 | 2.45E-005 | 1dbvO | ASCTTN |
| 1124 | 2.45E-005 | 1dbvO | INGFGR |
| 1125 | 2.45E-005 | 1dbvO | NGFGRIGRNVFRA |
| 1126 | 2.45E-005 | 1dbvO | PHKDLRRAR |
| 1127 | 2.45E-005 | 1dbvO | PTTTGAAKA |
| 1128 | 2.45E-005 | 1dbvO | SNASCTT |
| 1129 | 2.45E-005 | 1dbvO | SWYDNE |
| 1130 | 2.45E-005 | 1dbvO | TGAAKAVALV |
| 1131 | 2.45E-005 | 1dbvO | VISNAS |
| 1132 | 2.45E-005 | 1dbvO | KVVSWYDNE |
| 1133 | 2.45E-005 | 1dbvO | LDLPHKD |
| 1134 | 2.45E-005 | 1dbvO | TNDQRILD |
| 1135 | 2.45E-005 | 1dbvO | TTVHSYT |
| 1136 | 2.45E-005 | 1dbvO | VGINGFGRIGR |
| 1137 | 2.62E-005 | 1vbgA | GLPASP |
| 1138 | 2.62E-005 | 1vbgA | GTNDLTQ |
| 1139 | 2.62E-005 | 1vbgA | EHMFF |
| 1140 | 2.62E-005 | 1vbgA | ETSPEDVGGMHAA |
| 1141 | 2.62E-005 | 1vbgA | EVNPMLG |
| 1142 | 2.62E-005 | 1vbgA | FSFGTNDLTQ |
| 1143 | 2.62E-005 | 1vbgA | GICGEHGG |
| 1144 | 2.62E-005 | 1vbgA | GLPASPGAA |
| 1145 | 2.62E-005 | 1vbgA | KYRSINQITGL |
| 1146 | 2.62E-005 | 1vbgA | LDQLLHP |
| 1147 | 2.62E-005 | 1vbgA | LGGKGANLAEM |
| 1148 | 2.62E-005 | 1vbgA | MQDIEFT |
| 1149 | 2.62E-005 | 1vbgA | NAQGEDVVAG |
| 1150 | 2.62E-005 | 1vbgA | RGGMTSHAAVVA |
| 1151 | 2.62E-005 | 1vbgA | SMPGMMDT |
| 1152 | 4.68E-005 | 1t5aA | DLGIEI |
| 1153 | 4.68E-005 | 1t5aA | IPAEKV |
| 1154 | 4.68E-005 | 1t5aA | SKKGVN |
| 1155 | 4.68E-005 | 1t5aA | ATQMLESM |
| 1156 | 4.68E-005 | 1t5aA | DDGLISL |
| 1157 | 4.68E-005 | 1t5aA | DTKGPE |
| 1158 | 4.68E-005 | 1t5aA | GPASRSV |
| 1159 | 4.68E-005 | 1t5aA | GSKKGVN |
| 1160 | 4.68E-005 | 1t5aA | IYVDDG |
| 1161 | 4.68E-005 | 1t5aA | KGVNLP |
| 1162 | 4.68E-005 | 1t5aA | MLSGET |
| 1163 | 4.68E-005 | 1t5aA | MVARGDLG |
| 1164 | 4.68E-005 | 1t5aA | NFSHGTHEYH |
| 1165 | 4.68E-005 | 1t5aA | RLNFSHG |
| 1166 | 4.68E-005 | 1t5aA | RPTRAE |
| 1167 | 4.79E-005 | 1i10A | AYEVIK |
| 1168 | 4.79E-005 | 1i10A | GEHGDS |
| 1169 | 4.79E-005 | 1i10A | LGEHGD |
| 1170 | 4.79E-005 | 1i10A | VSNPVD |
| 1171 | 4.79E-005 | 1i10A | AYEVIKLKG |
| 1172 | 4.79E-005 | 1i10A | GEHGDSSV |
| 1173 | 4.79E-005 | 1i10A | GWVLGEHGD |
| 1174 | 4.79E-005 | 1i10A | IGSGCNLD |
| 1175 | 4.79E-005 | 1i10A | NPVDIL |
| 1176 | 4.79E-005 | 1i10A | SNPVDI |
| 1177 | 4.79E-005 | 1i10A | TAGARQ |
| 1178 | 4.95E-005 | 1oj4A | GLTLGAD |
| 1179 | 4.95E-005 | 1oj4A | LTPVEGVE |
| 1180 | 4.95E-005 | 1oj4A | AFAEGVGE |
| 1181 | 4.95E-005 | 1oj4A | EDNLIVRAARLLMK |
| 1182 | 4.95E-005 | 1oj4A | GGGSSNAAT |
| 1183 | 4.95E-005 | 1oj4A | GLGGGSS |
| 1184 | 4.95E-005 | 1oj4A | IRLLTPVEGVEH |
| 1185 | 4.95E-005 | 1oj4A | LGADVPVF |
| 1186 | 4.95E-005 | 1oj4A | LQTLFQF |
| 1187 | 4.95E-005 | 1oj4A | RADGYH |
| 1188 | 4.95E-005 | 1oj4A | TGTGACVF |
| 1189 | 5.09E-005 | 2gbcA | CTFITKGAWEVI |
| 1190 | 5.09E-005 | 2gbcA | GNSSIFLENSTF |
| 1191 | 5.09E-005 | 2gbcA | IEALTSDYLYYISN |
| 1192 | 5.09E-005 | 2gbcA | IPNNTQWITWS |
| 1193 | 5.09E-005 | 2gbcA | LRVLEDNSALDKMLQ |
| 1194 | 5.09E-005 | 2gbcA | RNLYKIQL |
| 1195 | 5.09E-005 | 2gbcA | VPLIEYSFYSDESLQYP |
| 1196 | 5.09E-005 | 2gbcA | VYAGPCSQK |
| 1197 | 5.19E-005 | 1e9iA | SIEDGL |
| 1198 | 5.19E-005 | 1e9iA | ANSILIK |
| 1199 | 5.19E-005 | 1e9iA | GDDLFVTN |
| 1200 | 5.19E-005 | 1e9iA | GQIKTGS |
| 1201 | 5.19E-005 | 1e9iA | IEDGLDE |
| 1202 | 5.19E-005 | 1e9iA | INGGEHADN |
| 1203 | 5.19E-005 | 1e9iA | KIQLVGDDLF |
| 1204 | 5.19E-005 | 1e9iA | SGETED |
| 1205 | 5.19E-005 | 1e9iA | SHRSGET |
| 1206 | 5.19E-005 | 1e9iA | TAVGDEGG |
| 1207 | 5.19E-005 | 1e9iA | TAVISHRSGE |
| 1208 | 5.19E-005 | 1e9iA | TLAMDCAASEF |
| 1209 | 5.23E-005 | 2i6lA | KIGDFG |
| 1210 | 5.23E-005 | 2i6lA | KHALREIKIIRRLDHDNIVKV |
| 1211 | 5.23E-005 | 2i6lA | LFAGAHELEQMQLIL |
| 1212 | 5.23E-005 | 2i6lA | TEDLVLKIGDFGLARI |
| 1213 | 6.06E-005 | 1mtoA | EVMGR |
| 1214 | 6.06E-005 | 1mtoA | TVLGH |
| 1215 | 6.06E-005 | 1mtoA | TVLGHV |
| 1216 | 6.06E-005 | 1mtoA | LGHVQRGG |
| 1217 | 6.06E-005 | 1mtoA | PGTIDND |
| 1218 | 6.06E-005 | 1mtoA | RVTVLGH |
| 1219 | 6.06E-005 | 1mtoA | TVLGHVQRGG |
| 1220 | 6.06E-005 | 1mtoA | VIGGDGS |
| 1221 | 6.06E-005 | 1mtoA | VLTSGGD |
| 1222 | 7.92E-005 | 1igwA | EAGFGG |
| 1223 | 7.92E-005 | 1igwA | SPSFNW |
| 1224 | 7.92E-005 | 1igwA | EVGTGYFD |
| 1225 | 7.92E-005 | 1igwA | GKVLVP |
| 1226 | 7.92E-005 | 1igwA | PGKLLAYNCSPSFNW |
| 1227 | 7.92E-005 | 1igwA | PIVADAEAGFGG |
| 1228 | 7.92E-005 | 1igwA | TPDLELA |
| 1229 | 7.92E-005 | 1igwA | VHFEDQLAS |
| 1230 | 7.92E-005 | 1igwA | YLSGWQVA |
| 1231 | 7.92E-005 | 1igwA | YPDQSLYPANSVP |
| 1232 | 8.64E-005 | 1auwA | AGKLHT |
| 1233 | 8.64E-005 | 1auwA | AVLQVATGVISTLQ |
| 1234 | 8.64E-005 | 1auwA | FSTGSS |
| 1235 | 8.64E-005 | 1auwA | GSMAYAKAL |
| 1236 | 8.64E-005 | 1auwA | GVISTLQI |
| 1237 | 8.64E-005 | 1auwA | HTANERRL |
| 1238 | 8.64E-005 | 1auwA | LKGLPSTYN |
| 1239 | 8.64E-005 | 1auwA | PFRQAH |
| 1240 | 8.64E-005 | 1auwA | PIRWSQFLLSHAVAL |
| 1241 | 8.64E-005 | 1auwA | PQKKNPDSLELIRSKAGRVFGR |
| 1242 | 8.64E-005 | 1auwA | RLKELIG |
| 1243 | 9.15E-005 | 1ob3A | DFGLAR |
| 1244 | 9.15E-005 | 1ob3A | ADFGLA |
| 1245 | 9.15E-005 | 1ob3A | HRDLKP |
| 1246 | 9.15E-005 | 1ob3A | IGEGTY |
| 1247 | 9.15E-005 | 1ob3A | ALKKIRL |
| 1248 | 9.15E-005 | 1ob3A | DFGLARAFG |
| 1249 | 9.15E-005 | 1ob3A | EKIGEG |
| 1250 | 9.15E-005 | 1ob3A | KIGEGT |
| 1251 | 9.15E-005 | 1ob3A | KPQNLL |
| 1252 | 9.15E-005 | 1ob3A | LHRDLKP |
| 1253 | 9.15E-005 | 1ob3A | VVTLWYR |
| 1254 | 9.29E-005 | 1igwC | EAGFGG |
| 1255 | 9.29E-005 | 1igwC | SPSFNW |
| 1256 | 9.29E-005 | 1igwC | EVGTGYFD |
| 1257 | 9.29E-005 | 1igwC | GKVLVP |
| 1258 | 9.29E-005 | 1igwC | PGKLLAYNCSPSFNW |
| 1259 | 9.29E-005 | 1igwC | PIVADAEAGFGG |
| 1260 | 9.29E-005 | 1igwC | TPDLELA |
| 1261 | 9.29E-005 | 1igwC | VHFEDQLAS |
| 1262 | 9.29E-005 | 1igwC | YLSGWQVA |
| 1263 | 9.29E-005 | 1igwC | YPDQSLYPANSVP |
| 1264 | 9.79E-005 | 13pkA | MGVFE |
| 1265 | 9.79E-005 | 13pkA | AHRDSATMTGIPK |
| 1266 | 9.79E-005 | 13pkA | ASAAELS |
| 1267 | 9.79E-005 | 13pkA | DIGPKT |
| 1268 | 9.79E-005 | 13pkA | GGAKVSDK |
| 1269 | 9.79E-005 | 13pkA | ITNDYRIR |
| 1270 | 9.79E-005 | 13pkA | KIQLLDNM |
| 1271 | 9.79E-005 | 13pkA | LDIGPK |
| 1272 | 9.79E-005 | 13pkA | LELLEGK |
| 1273 | 9.79E-005 | 13pkA | YTFLKAQG |
| 1274 | 1.13E-004 | 1jxaA | CGTSY |
| 1275 | 1.13E-004 | 1jxaA | IIENHE |
| 1276 | 1.13E-004 | 1jxaA | RNLAKSV |
| 1277 | 1.13E-004 | 1jxaA | ACGTSY |
| 1278 | 1.13E-004 | 1jxaA | ASDQLALL |
| 1279 | 1.13E-004 | 1jxaA | EIGVASTK |
| 1280 | 1.13E-004 | 1jxaA | GSPLVIG |
| 1281 | 1.13E-004 | 1jxaA | HNGIIEN |
| 1282 | 1.13E-004 | 1jxaA | QSGETAD |
| 1283 | 1.13E-004 | 1jxaA | RWATHG |
| 1284 | 1.13E-004 | 1jxaA | SETDTEV |
| 1285 | 1.13E-004 | 1jxaA | VASTKA |
| 1286 | 1.13E-004 | 1jxaA | VHNGII |
| 1287 | 1.17E-004 | 1i0zA | AYEVIK |
| 1288 | 1.17E-004 | 1i0zA | GEHGDS |
| 1289 | 1.17E-004 | 1i0zA | LGEHGD |
| 1290 | 1.17E-004 | 1i0zA | VSNPVD |
| 1291 | 1.17E-004 | 1i0zA | AYEVIKLKG |
| 1292 | 1.17E-004 | 1i0zA | GEHGDSSV |
| 1293 | 1.17E-004 | 1i0zA | GQVGMA |
| 1294 | 1.17E-004 | 1i0zA | IGSGCNLD |
| 1295 | 1.17E-004 | 1i0zA | LKGYTNWAIG |
| 1296 | 1.17E-004 | 1i0zA | NPVDIL |
| 1297 | 1.17E-004 | 1i0zA | SNPVDI |
| 1298 | 1.17E-004 | 1i0zA | VTAGVRQ |
| 1299 | 1.21E-004 | 1g6kA | LDVMINNAG |
| 1300 | 1.21E-004 | 1g6kA | ASYVTGITLFADGGMT |
| 1301 | 1.21E-004 | 1g6kA | IPWPLFVHYAASKGG |
| 1302 | 1.21E-004 | 1g6kA | KVVVNYRS |
| 1303 | 1.21E-004 | 1g6kA | MTETLALEYAPKGIRVNNIGPGA |
| 1304 | 1.21E-004 | 1g6kA | VINMSSVHE |
| 1305 | 1.44E-004 | 2hj2A | LDLKKPI |
| 1306 | 1.44E-004 | 2hj2A | YGHFGR |
| 1307 | 1.70E-004 | 1gv1A | TAGLPR |
| 1308 | 1.70E-004 | 1gv1A | GMAGVLD |
| 1309 | 1.70E-004 | 1gv1A | VLGGHGD |
| 1310 | 1.70E-004 | 1gv1A | KRVLPCA |
| 1311 | 1.70E-004 | 1gv1A | PRKPGM |
| 1312 | 1.70E-004 | 1gv1A | RTRNGG |
| 1313 | 1.70E-004 | 1gv1A | VLGGHGDAMVP |
| 1314 | 1.93E-004 | 1ee1A | IAVRLP |
| 1315 | 1.93E-004 | 1ee1A | GDGGAD |
| 1316 | 1.93E-004 | 1ee1A | SGGQDS |
| 1317 | 1.93E-004 | 1ee1A | FIAVRLP |
| 1318 | 1.93E-004 | 1ee1A | GTDHAAE |
| 1319 | 1.93E-004 | 1ee1A | PLTGLTKR |
| 1320 | 1.93E-004 | 1ee1A | RMIAQY |
| 1321 | 1.93E-004 | 1ee1A | SGGQDSTLAG |
| 1322 | 1.93E-004 | 1ee1A | TKYGDG |
| 1323 | 1.93E-004 | 1ee1A | VLGISGGQDS |
| 1324 | 2.03E-004 | 1fdyB | GVKLTLD |
| 1325 | 2.03E-004 | 1fdyB | SFEEHC |
| 1326 | 2.03E-004 | 1fdyB | IIDSADGLPMVVYNIPALSGV |
| 1327 | 2.03E-004 | 1fdyB | LQTECNKVIDLLIKTGVFRGLKTVLHYMDVVSVPLCRKPF |
| 1328 | 2.03E-004 | 1fdyB | PVDEKYLP |
| 1329 | 2.04E-004 | 1fdyA | GVKLTLD |
| 1330 | 2.04E-004 | 1fdyA | SFEEHC |
| 1331 | 2.04E-004 | 1fdyA | IIDSADGLPMVVYNIPALSGV |
| 1332 | 2.04E-004 | 1fdyA | LQTECNKVIDLLIKTGVFRGLKTVLHYMDVVSVPLCRKPF |
| 1333 | 2.04E-004 | 1fdyA | PVDEKYLP |
| 1334 | 2.17E-004 | 1e5tA | AYGLSASGSDWVTIKFMKVDGAKEL |
| 1335 | 2.17E-004 | 1e5tA | DHDDRVVP |
| 1336 | 2.17E-004 | 1e5tA | EIFYQFTSFLSPG |
| 1337 | 2.17E-004 | 1e5tA | FKKGKRYFYFYNTGLQNQRVLYVQDSLEGEARV |
| 1338 | 2.17E-004 | 1e5tA | GILKWVKLIDNFEGEYDYVTNEGTVFTFKTNR |
| 1339 | 2.17E-004 | 1e5tA | TIGHAWTTDYGCSD |
| 1340 | 2.17E-004 | 1e5tA | YGGFNIS |
| 1341 | 2.31E-004 | 2fug1 | GRGGAGF |
| 1342 | 2.31E-004 | 2fug1 | KPPFPA |
| 1343 | 2.31E-004 | 2fug1 | GAGAYICGEETA |
| 1344 | 2.31E-004 | 2fug1 | GLWGKPT |
| 1345 | 2.31E-004 | 2fug1 | NADESEP |
| 1346 | 2.31E-004 | 2fug1 | PGGSSTP |
| 1347 | 2.31E-004 | 2fug1 | PRLKPP |
| 1348 | 2.31E-004 | 1i0eA | KPVSPL |
| 1349 | 2.31E-004 | 1i0eA | IQTGVDNPG |
| 1350 | 2.31E-004 | 1i0eA | LIDDHFLF |
| 1351 | 2.31E-004 | 1i0eA | SSRVRTGR |
| 1352 | 2.31E-004 | 1i0eA | EQQQLIDDHFLFDKPVSPLL |
| 1353 | 2.31E-004 | 1i0eA | FLVWVNEEDHLRVISM |
| 1354 | 2.31E-004 | 1i0eA | IQTGVDNPGHP |
| 1355 | 2.31E-004 | 1i0eA | LTCPSNLGTGLR |
| 1356 | 2.31E-004 | 1i0eA | RLQKRGTGGVDT |
| 1357 | 2.31E-004 | 1i0eA | YVLSSRVRTGRSI |
| 1358 | 2.71E-004 | 1xwoA | AQPIRW |
| 1359 | 2.71E-004 | 1xwoA | DEDIHT |
| 1360 | 2.71E-004 | 1xwoA | DLQEDKE |
| 1361 | 2.71E-004 | 1xwoA | FSTGSS |
| 1362 | 2.71E-004 | 1xwoA | GSSLMPQKKN |
| 1363 | 2.71E-004 | 1xwoA | HTANERRL |
| 1364 | 2.71E-004 | 1xwoA | LKGLPSTYN |
| 1365 | 2.71E-004 | 1xwoA | MPQKKNPD |
| 1366 | 2.71E-004 | 1xwoA | PQKKNPDSLELIRSKAGRVFGR |
| 1367 | 2.94E-004 | 1a4zA | GGKSPNI |
| 1368 | 3.24E-004 | 1f4cB | FNIASY |
| 1369 | 3.24E-004 | 1f4cB | DRTGTGT |
| 1370 | 3.24E-004 | 1f4cB | HLYSNH |
| 1371 | 3.24E-004 | 1f4cB | IWDEW |
| 1372 | 3.24E-004 | 1f4cB | MALAPCH |
| 1373 | 3.31E-004 | 1v0bB | DFGLAR |
| 1374 | 3.31E-004 | 1v0bB | ADFGLA |
| 1375 | 3.31E-004 | 1v0bB | HRDLKP |
| 1376 | 3.31E-004 | 1v0bB | IGEGTY |
| 1377 | 3.31E-004 | 1v0bB | DFGLARAFG |
| 1378 | 3.31E-004 | 1v0bB | EKIGEG |
| 1379 | 3.31E-004 | 1v0bB | FGIPVR |
| 1380 | 3.31E-004 | 1v0bB | KIGEGT |
| 1381 | 3.31E-004 | 1v0bB | KPQNLL |
| 1382 | 3.31E-004 | 1v0bB | LHRDLKP |
| 1383 | 3.31E-004 | 1v0bB | VVTLWYR |
| 1384 | 3.68E-004 | 1orvA | CTFITKGAWEVI |
| 1385 | 3.68E-004 | 1orvA | FNAEYGNSS |
| 1386 | 3.68E-004 | 1orvA | GNSSIFLENSTF |
| 1387 | 3.68E-004 | 1orvA | IPNNTQWITWS |
| 1388 | 3.68E-004 | 1orvA | LRVLEDNSALDKMLQ |
| 1389 | 3.68E-004 | 1orvA | VPLIEYSFYSDESLQYP |
| 1390 | 3.68E-004 | 1orvA | VYAGPCSQK |
| 1391 | 3.68E-004 | 1orvA | YTERYM |
| 1392 | 3.76E-004 | 1brmC | ELATPS |
| 1393 | 3.76E-004 | 1brmC | VGGNCT |
| 1394 | 3.76E-004 | 1brmC | FFSTSQ |
| 1395 | 3.76E-004 | 1brmC | GGNCTVSLMLM |
| 1396 | 3.76E-004 | 1brmC | GWRGMVGSVL |
| 1397 | 3.76E-004 | 1brmC | HMRELLTQ |
| 1398 | 3.76E-004 | 1brmC | QGGDYT |
| 1399 | 3.76E-004 | 1brmC | WIDAAS |
| 1400 | 3.93E-004 | 1ej7L | HGIHFRVL |
| 1401 | 3.93E-004 | 1ej7L | KPKLGLS |
| 1402 | 3.93E-004 | 1ej7L | RGIYFTQ |
| 1403 | 3.93E-004 | 1ej7L | TLGFVD |
| 1404 | 3.93E-004 | 1ej7L | VASGGIH |
| 1405 | 3.93E-004 | 1ej7L | VLQFGGGT |
| 1406 | 4.06E-004 | 1brmA | ELATPS |
| 1407 | 4.06E-004 | 1brmA | VGGNCT |
| 1408 | 4.06E-004 | 1brmA | FFSTSQ |
| 1409 | 4.06E-004 | 1brmA | GGNCTVSLMLM |
| 1410 | 4.06E-004 | 1brmA | GWRGMVGSVL |
| 1411 | 4.06E-004 | 1brmA | HMRELLTQ |
| 1412 | 4.06E-004 | 1brmA | QGGDYT |
| 1413 | 4.06E-004 | 1brmA | WIDAAS |
| 1414 | 4.59E-004 | 1ajkA | FLGKDT |
| 1415 | 4.59E-004 | 1ajkA | DTTKVQFNYYTNG |
| 1416 | 4.59E-004 | 1ajkA | HTYAFDWQP |
| 1417 | 4.59E-004 | 1ajkA | KIMMNLW |
| 1418 | 4.59E-004 | 1ajkA | VSSFFTYTG |
| 1419 | 4.59E-004 | 1ajkA | WDEIDIEFLG |
| 1420 | 5.16E-004 | 2cfoA | KLSKRD |
| 1421 | 5.16E-004 | 2cfoA | FAHTPLILN |
| 1422 | 5.16E-004 | 2cfoA | KLSKRDG |
| 1423 | 5.16E-004 | 2cfoA | LHIGTARTAVFNWL |
| 1424 | 5.16E-004 | 2cfoA | RGEDHI |
| 1425 | 5.16E-004 | 2cfoA | VRVRLAPSPTG |
| 1426 | 5.69E-004 | 1o9jA | EEIFGP |
| 1427 | 5.69E-004 | 1o9jA | EIFGPV |
| 1428 | 5.69E-004 | 1o9jA | AGWADK |
| 1429 | 5.69E-004 | 1o9jA | AHQGVF |
| 1430 | 5.69E-004 | 1o9jA | GPTAGAAI |
| 1431 | 5.69E-004 | 1o9jA | MDASERG |
| 1432 | 5.69E-004 | 1o9jA | MRIAKEEIFGPV |
| 1433 | 5.69E-004 | 1o9jA | VTLELG |
| 1434 | 5.85E-004 | 1adoA | DFAKWRCVLKI |
| 1435 | 5.85E-004 | 1adoA | FSYGRALQ |
| 1436 | 5.85E-004 | 1adoA | GILAADES |
| 1437 | 5.85E-004 | 1adoA | GRALQAS |
| 1438 | 5.85E-004 | 1adoA | LEGTLLKPNMVT |
| 1439 | 5.85E-004 | 1adoA | LSGGQSE |
| 1440 | 5.85E-004 | 1adoA | PDGDHDL |
| 1441 | 5.97E-004 | 1yrpA | EFKNIFGTPEFVAPEIVNYEPLGLEADMWSIGVITYILLSGASPFLG |
| 1442 | 5.97E-004 | 1yrpA | EYAAKFIKKR |
| 1443 | 5.97E-004 | 1yrpA | GEELGSGQFAIV |
| 1444 | 5.97E-004 | 1yrpA | TDVVLILELVSGGELFDFLA |
| 1445 | 6.36E-004 | 1wxeA | IAVRLP |
| 1446 | 6.36E-004 | 1wxeA | VALGVT |
| 1447 | 6.36E-004 | 1wxeA | SGGQDS |
| 1448 | 6.36E-004 | 1wxeA | ALGVTY |
| 1449 | 6.36E-004 | 1wxeA | FIAVRLP |
| 1450 | 6.36E-004 | 1wxeA | GTDHAAE |
| 1451 | 6.36E-004 | 1wxeA | KARERMK |
| 1452 | 6.36E-004 | 1wxeA | KTEHKR |
| 1453 | 6.36E-004 | 1wxeA | LPYGVQ |
| 1454 | 6.36E-004 | 1wxeA | LVLGISGG |
| 1455 | 6.36E-004 | 1wxeA | SGGQDSTLAG |
| 1456 | 6.36E-004 | 1wxeA | TKYGDG |
| 1457 | 6.36E-004 | 1wxeA | VLGISGGQDS |
| 1458 | 7.12E-004 | 1fcjA | GAGFIP |
| 1459 | 7.12E-004 | 1fcjA | GVGTGG |
| 1460 | 7.12E-004 | 1fcjA | LLLQQFSN |
| 1461 | 7.12E-004 | 1fcjA | AAARGYK |
| 1462 | 7.12E-004 | 1fcjA | GISSGAA |
| 1463 | 7.12E-004 | 1fcjA | GMKGAI |
| 1464 | 7.12E-004 | 1fcjA | GYKLTLTMP |
| 1465 | 7.12E-004 | 1fcjA | IQGIGAGF |
| 1466 | 7.12E-004 | 1fcjA | LTMPETMS |
| 1467 | 7.12E-004 | 1fcjA | MSIERRKLLK |
| 1468 | 7.12E-004 | 1fcjA | SVKCRIGA |
| 1469 | 7.12E-004 | 1fcjA | TPLVRLNRI |
| 1470 | 7.12E-004 | 1fcjA | TSGNTGIALA |
| 1471 | 7.23E-004 | 1oroA | VITAGT |
| 1472 | 7.23E-004 | 1oroA | CFNRKE |
| 1473 | 7.23E-004 | 1oroA | DDVITAGT |
| 1474 | 7.23E-004 | 1oroA | FGPAYKGI |
| 1475 | 7.23E-004 | 1oroA | GLFNTG |
| 1476 | 7.23E-004 | 1oroA | KDHGEGG |
| 1477 | 7.23E-004 | 1oroA | SAIQEVERD |
| 1478 | 7.23E-004 | 1oroA | SPYFFNAG |
| 1479 | 7.23E-004 | 1oroA | TAGTAI |
| 1480 | 8.94E-004 | 1be4A | GRNIIH |
| 1481 | 8.94E-004 | 1be4A | GTIRGD |
| 1482 | 8.94E-004 | 1be4A | HGSDSV |
| 1483 | 8.94E-004 | 1be4A | IIKRFEQKGFRLVAMKF |
| 1484 | 8.94E-004 | 1be4A | NIIHGSD |
| 1485 | 8.94E-004 | 1be4A | TIRGDF |
| 1486 | 9.13E-004 | 1wdkC | NEAFAAQ |
| 1487 | 9.13E-004 | 1wdkC | GMMGLTAEML |
| 1488 | 9.13E-004 | 1wdkC | RTPMGRSKGG |
| 1489 | 9.20E-004 | 1xm0A | INSAAL |
| 1490 | 9.20E-004 | 1xm0A | CGWPSF |
| 1491 | 9.20E-004 | 1xm0A | GTEPPF |
| 1492 | 9.20E-004 | 1xm0A | HLGHVFNDGP |
| 1493 | 9.20E-004 | 1xm0A | LRYCINSAALRF |
| 1494 | 9.20E-004 | 1xm0A | RTEVRS |
| 1495 | 9.20E-004 | 1xm0A | SHLGHVF |
| 1496 | 9.20E-004 | 1xm0A | YVDIVSG |
| 1497 | 9.39E-004 | 1dqwA | ADIGN |
| 1498 | 9.39E-004 | 1dqwA | ADIGNT |
| 1499 | 9.39E-004 | 1dqwA | DIGNTV |
| 1500 | 9.39E-004 | 1dqwA | DWLIMTPGVGL |
| 1501 | 9.39E-004 | 1dqwA | GFIAQR |
| 1502 | 9.39E-004 | 1dqwA | GQQYRT |
| 1503 | 9.39E-004 | 1dqwA | IIVGRG |
| 1504 | 9.39E-004 | 1dqwA | KGSLATGEY |
| 1505 | 9.39E-004 | 1dqwA | LGQQYR |
| 1506 | 9.83E-004 | 1ebdA | CLNVGCIP |
| 1507 | 9.83E-004 | 1ebdA | GCIPSKAL |
| 1508 | 9.83E-004 | 1ebdA | GGYVAAI |
| 1509 | 9.83E-004 | 1ebdA | GLLKGNKV |
| 1510 | 9.83E-004 | 1ebdA | KVAAEAI |
| 1511 | 9.83E-004 | 1ebdA | TIHAHPTL |
| 1512 | 9.83E-004 | 1ebdA | TVGRRPNTDELGLE |
| 1513 | 1.01E-003 | 1s4bP | QDGSRQ |
| 1514 | 1.01E-003 | 1s4bP | ATPGTNMPATFGHLAGGYD |
| 1515 | 1.01E-003 | 1s4bP | DFVEAPSQMLE |
| 1516 | 1.01E-003 | 1s4bP | HAACFGLQPGCL |
| 1517 | 1.01E-003 | 1s4bP | LCIDFNKNLNED |
| 1518 | 1.01E-003 | 1s4bP | QYYGYLWSEV |
| 1519 | 1.01E-003 | 1s4bP | TLKYPHYFP |
| 1520 | 1.01E-003 | 1s4bP | TYFHEFGHVMHQ |
| 1521 | 1.18E-003 | 1k3tA | GFGRIG |
| 1522 | 1.18E-003 | 1k3tA | ASCTTN |
| 1523 | 1.18E-003 | 1k3tA | INGFGR |
| 1524 | 1.18E-003 | 1k3tA | KDWRGGR |
| 1525 | 1.18E-003 | 1k3tA | SNASCTT |
| 1526 | 1.18E-003 | 1k3tA | SWYDNE |
| 1527 | 1.18E-003 | 1k3tA | WYDNEWG |
| 1528 | 1.18E-003 | 1k3tA | HSYTATQK |
| 1529 | 1.18E-003 | 1k3tA | KVVISAP |
| 1530 | 1.18E-003 | 1k3tA | TATQKTV |
| 1531 | 1.18E-003 | 1k3tA | TGAAKAVG |
| 1532 | 1.18E-003 | 1k3tA | TTIHSYT |
| 1533 | 1.18E-003 | 1k3tA | VGINGFGRIGR |
| 1534 | 1.18E-003 | 1k3tA | VSWYDNEWGYS |
| 1535 | 1.22E-003 | 2gl6E | RGIWHN |
| 1536 | 1.22E-003 | 2gl6E | GRSEVELVQIVIDGVNYLVDCEKKLE |
| 1537 | 1.22E-003 | 2gl6E | RHNGYDPR |
| 1538 | 1.22E-003 | 2gl6E | RLQKRGTGGVDT |
| 1539 | 1.22E-003 | 1q24A | FLAKAK |
| 1540 | 1.22E-003 | 1q24A | KPENLL |
| 1541 | 1.22E-003 | 1q24A | LGTGSF |
| 1542 | 1.22E-003 | 1q24A | TGSFGR |
| 1543 | 1.22E-003 | 1q24A | CGTPEY |
| 1544 | 1.22E-003 | 1q24A | RDLKPENLL |
| 1545 | 1.22E-003 | 1q24A | YRDLKPEN |
| 1546 | 1.22E-003 | 1q24A | DFGFAK |
| 1547 | 1.22E-003 | 1q24A | ESVKEFLAKAKE |
| 1548 | 1.22E-003 | 1q24A | GTGSFG |
| 1549 | 1.22E-003 | 1q24A | GTPEYL |
| 1550 | 1.22E-003 | 1q24A | RDLKPENLLID |
| 1551 | 1.23E-003 | 1k9aA | IVTEYM |
| 1552 | 1.23E-003 | 1k9aA | GRVPYP |
| 1553 | 1.23E-003 | 1k9aA | HRDLAARN |
| 1554 | 1.23E-003 | 1k9aA | KGDVLTIVAVTKDPNWYKAKNKVGREGIIPANYVQKREGVKAG |
| 1555 | 1.23E-003 | 1k9aA | WPSGTECIAKYNFHGTAEQDLPF |
| 1556 | 1.29E-003 | 1euqA | TRFPPEP |
| 1557 | 1.29E-003 | 1euqA | CTLEF |
| 1558 | 1.29E-003 | 1euqA | EFSRLN |
| 1559 | 1.29E-003 | 1euqA | GHAKSICLNF |
| 1560 | 1.29E-003 | 1euqA | IYPMYDF |
| 1561 | 1.29E-003 | 1euqA | MSKRKL |
| 1562 | 1.29E-003 | 1euqA | QYEFSRL |
| 1563 | 1.29E-003 | 1euqA | SKRKLNLLV |
| 1564 | 1.29E-003 | 1euqA | TRFPPEPNG |
| 1565 | 1.29E-003 | 1euqA | YDFTHC |
| 1566 | 1.42E-003 | 1d8cA | ANWLRHG |
| 1567 | 1.42E-003 | 1d8cA | FINTGFLDRTGDE |
| 1568 | 1.42E-003 | 1d8cA | GCSKVPDI |
| 1569 | 1.42E-003 | 1d8cA | LGYVVRW |
| 1570 | 1.42E-003 | 1d8cA | LMEDRATLRISSQ |
| 1571 | 1.42E-003 | 1d8cA | MDEERRT |
| 1572 | 1.42E-003 | 1d8cA | MEAGPM |
| 1573 | 1.42E-003 | 1d8cA | QHIANWL |
| 1574 | 1.42E-003 | 1d8cA | TAWVPSPTAATLHA |
| 1575 | 1.42E-003 | 1d8cA | TPWIKAYERNNV |
| 1576 | 1.42E-003 | 1d8cA | YIVKPK |
| 1577 | 1.48E-003 | 1hqsA | HKGNI |
| 1578 | 1.48E-003 | 1hqsA | NVALRQELD |
| 1579 | 1.48E-003 | 1hqsA | SLNVALR |
| 1580 | 1.48E-003 | 1hqsA | FLQQIL |
| 1581 | 1.48E-003 | 1hqsA | GVPSPVKRP |
| 1582 | 1.48E-003 | 1hqsA | KGNIMK |
| 1583 | 1.48E-003 | 1hqsA | KGPLTTP |
| 1584 | 1.48E-003 | 1hqsA | QQILTR |
| 1585 | 1.48E-003 | 1hqsA | RSLNVALRQ |
| 1586 | 1.48E-003 | 1hqsA | SDALAAQVGGIGI |
| 1587 | 1.48E-003 | 1hqsA | VGGIGIAPGAN |
| 1588 | 1.48E-003 | 1hqsA | VTLVHKGNI |
| 1589 | 1.48E-003 | 1hqsA | VTYDFARLMDGA |
| 1590 | 1.60E-003 | 1akmA | FHPTQ |
| 1591 | 1.60E-003 | 1akmA | SQIGH |
| 1592 | 1.60E-003 | 1akmA | EFHPTQ |
| 1593 | 1.60E-003 | 1akmA | GDARNN |
| 1594 | 1.60E-003 | 1akmA | GPSGSQIG |
| 1595 | 1.60E-003 | 1akmA | IALIFEK |
| 1596 | 1.60E-003 | 1akmA | IGHKES |
| 1597 | 1.60E-003 | 1akmA | STRTRC |
| 1598 | 1.60E-003 | 1akmA | VLGRMYD |
| 1599 | 1.60E-003 | 1akmA | VTYLGP |
| 1600 | 1.62E-003 | 1vl2A | GFWFSP |
| 1601 | 1.62E-003 | 1vl2A | GNDQVRF |
| 1602 | 1.62E-003 | 1vl2A | GRLDMVE |
| 1603 | 1.62E-003 | 1vl2A | HGATGKGNDQ |
| 1604 | 1.62E-003 | 1vl2A | IARPLIAK |
| 1605 | 1.62E-003 | 1vl2A | KSRGVYETPG |
| 1606 | 1.62E-003 | 1vl2A | KVVLAYSGG |
| 1607 | 1.62E-003 | 1vl2A | SGGLDT |
| 1608 | 1.62E-003 | 1vl2A | VGQKDDF |
| 1609 | 1.62E-003 | 1vl2A | YNGFWF |
| 1610 | 1.98E-003 | 2i3aA | AVSGTSGAGRA |
| 1611 | 1.98E-003 | 2i3aA | GASGYAG |
| 1612 | 1.98E-003 | 2i3aA | HRHTPE |
| 1613 | 1.98E-003 | 2i3aA | IAVPGC |
| 1614 | 1.98E-003 | 2i3aA | LGEHHPHLTPLA |
| 1615 | 1.98E-003 | 2i3aA | PGCYPT |
| 1616 | 1.98E-003 | 2i3aA | YLMPEGQLP |
| 1617 | 2.03E-003 | 1liuA | NIVRVV |
| 1618 | 2.03E-003 | 1liuA | IAREAEAA |
| 1619 | 2.03E-003 | 1liuA | RPTRAE |
| 1620 | 2.28E-003 | 1hdiA | GGMAFTF |
| 1621 | 2.28E-003 | 1hdiA | GLDCGPES |
| 1622 | 2.28E-003 | 1hdiA | STGGGASL |
| 1623 | 2.31E-003 | 2gl6A | GRSEVELVQIVIDGVNYLVDCEKKLE |
| 1624 | 2.31E-003 | 2gl6A | RHNGYDPR |
| 1625 | 2.31E-003 | 2gl6A | RLQKRGTGGVDT |
| 1626 | 2.36E-003 | 1tcoA | EKVTEM |
| 1627 | 2.36E-003 | 1tcoA | VMNIR |
| 1628 | 2.36E-003 | 1tcoA | MNIRQ |
| 1629 | 2.36E-003 | 1tcoA | FLGDYVDRG |
| 1630 | 2.36E-003 | 1tcoA | FMDVFTWSLPFVGEKVTEMLV |
| 1631 | 2.36E-003 | 1tcoA | NVMNIRQF |
| 1632 | 2.36E-003 | 1tcoA | TGFPSLIT |
| 1633 | 2.36E-003 | 1tcoA | VALRIITEGA |
| 1634 | 2.36E-003 | 1tcoA | VRGCSY |
| 1635 | 2.66E-003 | 1v8bA | DDGGDAT |
| 1636 | 2.66E-003 | 1v8bA | EETTTG |
| 1637 | 2.66E-003 | 1v8bA | GCLHMT |
| 1638 | 2.66E-003 | 1v8bA | GDVGKG |
| 1639 | 2.66E-003 | 1v8bA | LPKHLDE |
| 1640 | 2.66E-003 | 1v8bA | NIGHFD |
| 1641 | 2.73E-003 | 1j2eA | SFDGRG |
| 1642 | 2.73E-003 | 1j2eA | FNAEYGNSS |
| 1643 | 2.73E-003 | 1j2eA | LRVLEDNSALDKMLQ |
| 1644 | 2.73E-003 | 1j2eA | MWYTDEDHGIAS |
| 1645 | 2.73E-003 | 1j2eA | TFLAYAQFNDT |
| 1646 | 2.73E-003 | 1j2eA | VPLIEYSFYSDESLQYP |
| 1647 | 2.99E-003 | 1dhpA | AIVSVGTTGE |
| 1648 | 2.99E-003 | 1dhpA | EPNPI |
| 1649 | 2.99E-003 | 1dhpA | GTTGES |
| 1650 | 2.99E-003 | 1dhpA | GVISVTAN |
| 1651 | 2.99E-003 | 1dhpA | PYYNRP |
| 1652 | 2.99E-003 | 1dhpA | RTGCDLLP |
| 1653 | 2.99E-003 | 1dhpA | VPSRTG |
| 1654 | 2.99E-003 | 1dhpA | YNVPSR |
| 1655 | 2.99E-003 | 1gw6A | GGMENPCLTFVTP |
| 1656 | 2.99E-003 | 1gw6A | NSEIRF |
| 1657 | 2.99E-003 | 1gw6A | ALGGWGELQN |
| 1658 | 2.99E-003 | 1gw6A | YGGMENPCLTFVTPTLLAGDKSLSNVIAHEISH |
| 1659 | 3.02E-003 | 1wkbA | LHVGH |
| 1660 | 3.02E-003 | 1wkbA | GHARTY |
| 1661 | 3.02E-003 | 1wkbA | KMSKSK |
| 1662 | 3.02E-003 | 1wkbA | SKSKGN |
| 1663 | 3.02E-003 | 1wkbA | ILPAEFVD |
| 1664 | 3.02E-003 | 1wkbA | KDLIPNHLTF |
| 1665 | 3.05E-003 | 1c7gA | HQGRGAE |
| 1666 | 3.05E-003 | 1c7gA | YIDLLTDSGTNAMSD |
| 1667 | 3.05E-003 | 1c7gA | CLAVTVNLAGGQPVSM |
| 1668 | 3.05E-003 | 1c7gA | DGCTMSGKKDCLVNIGGFL |
| 1669 | 3.05E-003 | 1c7gA | FGFKHIVPTHQGRGAEN |
| 1670 | 3.05E-003 | 1c7gA | GNMYFTTTR |
| 1671 | 3.05E-003 | 1c7gA | MERGIVSAGR |
| 1672 | 3.05E-003 | 1c7gA | PKLETVR |
| 1673 | 3.27E-003 | 1dmtA | TLGENIADNGG |
| 1674 | 3.27E-003 | 1dmtA | DLNHKQLFFLNFAQVWCGTYRPEYAVNSIKTDVHSPGNFRIIG |
| 1675 | 3.35E-003 | 1f0yA | HFFNPVP |
| 1676 | 3.35E-003 | 1f0yA | ANATTRQDRFAGLHFFNPVP |
| 1677 | 3.35E-003 | 1f0yA | LGKHPVSCKDTPGFIVNRLLVPYL |
| 1678 | 3.60E-003 | 1egcA | GAVGLAQRALDEATKYAL |
| 1679 | 3.60E-003 | 1egcA | IKTKAEKKGDEY |
| 1680 | 3.68E-003 | 1dfoB | HGGHL |
| 1681 | 3.68E-003 | 1dfoB | TTHKT |
| 1682 | 3.68E-003 | 1dfoB | GGHLTHG |
| 1683 | 3.68E-003 | 1dfoB | HSGSQAN |
| 1684 | 3.68E-003 | 1dfoB | LAHGGHL |
| 1685 | 3.68E-003 | 1dfoB | NKNSVP |
| 1686 | 3.68E-003 | 1dfoB | TTHKTL |
| 1687 | 3.68E-003 | 1dfoB | TTTTHK |
| 1688 | 3.80E-003 | 1a7kA | GFGRIG |
| 1689 | 3.80E-003 | 1a7kA | ASCTTN |
| 1690 | 3.80E-003 | 1a7kA | INGFGR |
| 1691 | 3.80E-003 | 1a7kA | KDWRGGR |
| 1692 | 3.80E-003 | 1a7kA | SWYDNE |
| 1693 | 3.80E-003 | 1a7kA | HSYTATQK |
| 1694 | 3.80E-003 | 1a7kA | KVVISAP |
| 1695 | 3.80E-003 | 1a7kA | KVVSWYDNE |
| 1696 | 3.80E-003 | 1a7kA | TATQKTV |
| 1697 | 3.80E-003 | 1a7kA | TGAAKAVG |
| 1698 | 3.80E-003 | 1a7kA | TTIHSYT |
| 1699 | 3.80E-003 | 1a7kA | VGINGFGRIGR |
| 1700 | 3.84E-003 | 1kfuL | EDGEFWM |
| 1701 | 3.84E-003 | 1kfuL | GATRTDICQGALGDCWLLAAI |
| 1702 | 3.84E-003 | 1kfuL | GHAYSVTGA |
| 1703 | 3.84E-003 | 1kfuL | GSLLGCSI |
| 1704 | 3.84E-003 | 1kfuL | KLVKGHAYSVT |
| 1705 | 3.84E-003 | 1kfuL | DFLRHYSRLEICNLTPDTL |
| 1706 | 3.84E-003 | 1kfuL | GGATRTDICQGALGDCWLLAAIASLTLNEEILARVVP |
| 1707 | 3.84E-003 | 1kfuL | QKLVKGHAYSVTGAEEV |
| 1708 | 4.00E-003 | 1h6vA | GGSGGLA |
| 1709 | 4.00E-003 | 1h6vA | GGTCVNV |
| 1710 | 4.00E-003 | 1h6vA | VGASYV |
| 1711 | 4.00E-003 | 1h6vA | ELTPVAI |
| 1712 | 4.00E-003 | 1h6vA | GGGSGGLA |
| 1713 | 4.00E-003 | 1h6vA | IGGGSGG |
| 1714 | 4.00E-003 | 1h6vA | QLDSTIGIHPVCAE |
| 1715 | 4.00E-003 | 1h6vA | TTVFTPLEY |
| 1716 | 4.00E-003 | 1h6vA | TVMVRSI |
| 1717 | 4.00E-003 | 1h6vA | VGVKINEKTGKIPVT |
| 1718 | 4.20E-003 | 1e9iD | ANSILIK |
| 1719 | 4.20E-003 | 1e9iD | GDDLFVTN |
| 1720 | 4.20E-003 | 1e9iD | KIQLVGDDLF |
| 1721 | 4.20E-003 | 1e9iD | PSGASTG |
| 1722 | 4.20E-003 | 1e9iD | SGETED |
| 1723 | 4.20E-003 | 1e9iD | SHRSGET |
| 1724 | 4.20E-003 | 1e9iD | TAVISHRSGE |
| 1725 | 4.20E-003 | 1e9iD | TLAMDCAASEF |
| 1726 | 4.43E-003 | 2ht5A | DTPRGED |
| 1727 | 4.43E-003 | 2ht5A | ILRTQESSCTCI |
| 1728 | 4.43E-003 | 2ht5A | LVISPDLSY |
| 1729 | 4.43E-003 | 2ht5A | RTQESSCTCIQG |
| 1730 | 4.45E-003 | 1np3A | LHELKL |
| 1731 | 4.45E-003 | 1np3A | APKAPGHTVR |
| 1732 | 4.45E-003 | 1np3A | DLFGEQAVL |
| 1733 | 4.45E-003 | 1np3A | ETDLFGEQ |
| 1734 | 4.45E-003 | 1np3A | GSQGHAHA |
| 1735 | 4.45E-003 | 1np3A | GYGSQGHA |
| 1736 | 4.45E-003 | 1np3A | KLIVDLM |
| 1737 | 4.45E-003 | 1np3A | LHELKLIVDL |
| 1738 | 4.45E-003 | 1np3A | SNNAEYG |
| 1739 | 4.74E-003 | 1lewA | DFGLAR |
| 1740 | 4.74E-003 | 1lewA | HRDLKP |
| 1741 | 4.74E-003 | 1lewA | IHRDLK |
| 1742 | 4.74E-003 | 1lewA | RDLKPSN |
| 1743 | 4.74E-003 | 1lewA | ADPYDQSFESRDL |
| 1744 | 4.74E-003 | 1lewA | HRDLKPSN |
| 1745 | 4.74E-003 | 1lewA | ILDFGLA |
| 1746 | 4.74E-003 | 1lewA | KKLSRPFQ |
| 1747 | 4.74E-003 | 1lewA | LMGADL |
| 1748 | 4.74E-003 | 1lewA | RTYREL |
| 1749 | 4.74E-003 | 1lewA | VYLVTHLM |
| 1750 | 4.74E-003 | 1lewA | YRELRLLKHM |
| 1751 | 4.96E-003 | 1civA | DENRAK |
| 1752 | 4.96E-003 | 1civA | PRGPGMER |
| 1753 | 4.96E-003 | 1civA | VVGNPCNTN |
| 1754 | 4.96E-003 | 1civA | VVGNPCNTNALICLKN |
| 1755 | 4.96E-003 | 1civA | WALLIGAKPRGPGMERA |
| 1756 | 5.14E-003 | 2hroA | LTPSDT |
| 1757 | 5.14E-003 | 2hroA | AIAKAYLLVEPDL |
| 1758 | 5.14E-003 | 2hroA | DLTPSDTA |
| 1759 | 5.14E-003 | 2hroA | ELTKIRNNAE |
| 1760 | 5.14E-003 | 2hroA | GGRTSHSAIM |
| 1761 | 5.14E-003 | 2hroA | HILGVELPNPS |
| 1762 | 5.14E-003 | 2hroA | KAAIFDAH |
| 1763 | 5.14E-003 | 2hroA | RSLEIPA |
| 1764 | 5.44E-003 | 1z7wA | GAGFIP |
| 1765 | 5.44E-003 | 1z7wA | GIGTGGT |
| 1766 | 5.44E-003 | 1z7wA | GISSGAA |
| 1767 | 5.44E-003 | 1z7wA | GMKGAI |
| 1768 | 5.44E-003 | 1z7wA | IQGIGAGF |
| 1769 | 5.44E-003 | 1z7wA | MEPCSSVKDRIG |
| 1770 | 5.44E-003 | 1z7wA | QFENPAN |
| 1771 | 5.44E-003 | 1z7wA | SVKDRIGFSMISDAE |
| 1772 | 5.44E-003 | 1z7wA | SVLIEPTSGNTG |
| 1773 | 5.46E-003 | 1a4uA | INGAGI |
| 1774 | 5.46E-003 | 1a4uA | ANICSVTGFN |
| 1775 | 5.46E-003 | 1a4uA | LGGIGLDTSRELVKR |
| 1776 | 5.46E-003 | 1a4uA | LINGAGIL |
| 1777 | 5.46E-003 | 1a4uA | NGAGILDD |
| 1778 | 5.46E-003 | 1a4uA | VEPRVAELLL |
| 1779 | 5.46E-003 | 1a4uA | YSINPGIT |
| 1780 | 5.59E-003 | 1a2n | KVSVGA |
| 1781 | 5.59E-003 | 1a2n | LPGGC |
| 1782 | 5.59E-003 | 1a2n | VSVGAT |
| 1783 | 5.59E-003 | 1a2n | ETVFENR |
| 1784 | 5.59E-003 | 1a2n | IGARPVD |
| 1785 | 5.59E-003 | 1a2n | KNAALPI |
| 1786 | 5.59E-003 | 1a2n | LVKTMRAS |
| 1787 | 5.59E-003 | 1a2n | MDKVSVGAT |
| 1788 | 5.59E-003 | 1a2n | RPVDLH |
| 1789 | 5.59E-003 | 1a2n | VSLPGGC |
| 1790 | 5.83E-003 | 1bg0 | LIDDHFLF |
| 1791 | 5.83E-003 | 1bg0 | LIDDHFLFKEGDRFLQ |
| 1792 | 5.83E-003 | 1bg0 | LQVRGTRGEHTE |
| 1793 | 5.83E-003 | 1bg0 | NEEDHLRIISM |
| 1794 | 5.83E-003 | 1bg0 | NLDSGVG |
| 1795 | 5.83E-003 | 1bg0 | STRVRCGRS |
| 1796 | 5.85E-003 | 2gl6H | GRSEVELVQIVIDGVNYLVDCEKKLE |
| 1797 | 5.85E-003 | 2gl6H | RHNGYDPR |
| 1798 | 5.85E-003 | 2gl6H | RLQKRGTGGVDT |
| 1799 | 6.52E-003 | 1fwxA | GNKVRV |
| 1800 | 6.52E-003 | 1fwxA | FDGRGNAYT |
| 1801 | 6.52E-003 | 1fwxA | GEKVDPI |
| 1802 | 6.52E-003 | 1fwxA | GVYWYYC |
| 1803 | 6.52E-003 | 1fwxA | KWNIEDA |
| 1804 | 6.89E-003 | 1te6A | IGMDVAAS |
| 1805 | 6.89E-003 | 1te6A | KYGKDATNVG |
| 1806 | 6.89E-003 | 1te6A | LKVNQIG |
| 1807 | 6.89E-003 | 1te6A | LLLKVNQ |
| 1808 | 6.89E-003 | 1te6A | PSGASTG |
| 1809 | 6.89E-003 | 1te6A | SGETED |
| 1810 | 6.89E-003 | 1te6A | SHRSGET |
| 1811 | 6.89E-003 | 1te6A | VGDDLTVT |
| 1812 | 6.90E-003 | 1ibqA | YGDGSS |
| 1813 | 6.90E-003 | 1ibqA | GGIQSNSGLGLSILGDVFLKSQYVVF |
| 1814 | 6.95E-003 | 1dliA | AGSGYVGLSL |
| 1815 | 6.95E-003 | 1dliA | IFSPEFLRE |
| 1816 | 6.95E-003 | 1dliA | IIATPTNY |
| 1817 | 6.95E-003 | 1dliA | KALYDNLYPSRI |
| 1818 | 6.95E-003 | 1dliA | KLFANTYLA |
| 1819 | 6.95E-003 | 1dliA | YNNPSFGYGGYCLPKDTKQL |
| 1820 | 6.95E-003 | 1dliA | YRLIMKS |
| 1821 | 7.02E-003 | 1euzA | DIPAPDV |
| 1822 | 7.02E-003 | 1euzA | GGVTVSYFEW |
| 1823 | 7.02E-003 | 1euzA | NAGGVTVS |
| 1824 | 7.02E-003 | 1euzA | PTKGGIR |
| 1825 | 7.02E-003 | 1euzA | EKGILQIPDFLCNAGGVTVSYFEWVQNI |
| 1826 | 7.02E-003 | 1euzA | ITGKPLSIGGSLGR |
| 1827 | 7.02E-003 | 1euzA | NWARGPTKGGIRWHP |
| 1828 | 7.02E-003 | 1euzA | QGYGNAGYY |
| 1829 | 7.22E-003 | 1f1oA | QAKAGIE |
| 1830 | 7.22E-003 | 1f1oA | GKISGAVG |
| 1831 | 7.22E-003 | 1f1oA | LWHERD |
| 1832 | 7.23E-003 | 1pmmA | INDELY |
| 1833 | 7.23E-003 | 1pmmA | AINFSR |
| 1834 | 7.23E-003 | 1pmmA | ADLWHAP |
| 1835 | 7.23E-003 | 1pmmA | GSSEACMLGG |
| 1836 | 7.23E-003 | 1pmmA | GWQVPA |
| 1837 | 7.23E-003 | 1pmmA | QIINDEL |
| 1838 | 7.23E-003 | 1pmmA | RSELLDSRFGAK |
| 1839 | 7.23E-003 | 1pmmC | INDELY |
| 1840 | 7.23E-003 | 1pmmC | AINFSR |
| 1841 | 7.23E-003 | 1pmmC | ADLWHAP |
| 1842 | 7.23E-003 | 1pmmC | GSSEACMLGG |
| 1843 | 7.23E-003 | 1pmmC | GWQVPA |
| 1844 | 7.23E-003 | 1pmmC | QIINDEL |
| 1845 | 7.23E-003 | 1pmmC | RSELLDSRFGAK |
| 1846 | 7.51E-003 | 1ne7A | ANARFF |
| 1847 | 7.51E-003 | 1ne7A | GHIAFNEP |
| 1848 | 7.51E-003 | 1ne7A | HPESYHSFM |
| 1849 | 7.51E-003 | 1ne7A | TFNMDEY |
| 1850 | 7.60E-003 | 1g59A | LRAALTG |
| 1851 | 7.60E-003 | 1g59A | PHVGTAY |
| 1852 | 7.60E-003 | 1g59A | PTYHLAN |
| 1853 | 7.60E-003 | 1g59A | RIAPSPTG |
| 1854 | 7.60E-003 | 1g59A | RIEDTD |
| 1855 | 7.63E-003 | 1yp2A | ILAGDH |
| 1856 | 7.63E-003 | 1yp2A | MGIYV |
| 1857 | 7.63E-003 | 1yp2A | TQFNS |
| 1858 | 7.63E-003 | 1yp2A | ASMGIYV |
| 1859 | 7.63E-003 | 1yp2A | GDHLYRM |
| 1860 | 7.63E-003 | 1yp2A | NDFGSE |
| 1861 | 7.63E-003 | 1yp2A | WFQGTA |
| 1862 | 7.63E-003 | 1yp2A | YWEDIGT |
| 1863 | 7.74E-003 | 1ypfA | IADGGI |
| 1864 | 7.74E-003 | 1ypfA | AGADATKVGIGPGKVCITK |
| 1865 | 7.74E-003 | 1ypfA | KPIIADGGIRT |
| 1866 | 7.74E-003 | 1ypfA | MIGSLFAGH |
| 1867 | 7.74E-003 | 1ypfA | MVMIGS |
| 1868 | 7.74E-003 | 1ypfA | PANMQT |
| 1869 | 8.61E-003 | 1pr9A | VTGAGKGIG |
| 1870 | 8.61E-003 | 1pr9A | LVTGAGKGIGR |
| 1871 | 8.61E-003 | 1pr9A | VGPVDLLVNNAAVA |
| 1872 | 8.61E-003 | 1pr9A | VNPTVVMT |
| 1873 | 8.61E-003 | 1pr9A | MALELGPHKIRVNAVNPTVVMT |
| 1874 | 8.90E-003 | 1a50B | AETGAGQHG |
| 1875 | 8.90E-003 | 1a50B | CVGGGSN |
| 1876 | 8.90E-003 | 1a50B | GGGSNAIG |
| 1877 | 8.90E-003 | 1a50B | GRGDKDIFTV |
| 1878 | 8.90E-003 | 1a50B | HGGAHK |
| 1879 | 8.90E-003 | 1a50B | RQSPNVFRM |
| 1880 | 8.90E-003 | 1a50B | SGRGDKD |
| 1881 | 8.90E-003 | 1a50B | SISAGLDFP |
| 1882 | 8.90E-003 | 1a50B | VGGGSNA |
| 1883 | 8.91E-003 | 1jwhA | GRGKYS |
| 1884 | 8.91E-003 | 1jwhA | VKPHN |
| 1885 | 8.91E-003 | 1jwhA | GRGKYSEVF |
| 1886 | 8.91E-003 | 1jwhA | HRDVKP |
| 1887 | 8.91E-003 | 1jwhA | NNTDFK |
| 1888 | 8.91E-003 | 1jwhA | NVMIDH |
| 1889 | 8.91E-003 | 1jwhA | RKLRLIDWGLAEFYHPG |
| 1890 | 9.25E-003 | 1clkA | DQDLRFG |
| 1891 | 9.25E-003 | 1clkA | EPKPNEPRGDI |
| 1892 | 9.25E-003 | 1clkA | GRDPFGD |
| 1893 | 9.25E-003 | 1clkA | IEPKP |
| 1894 | 9.25E-003 | 1clkA | TFHDDDL |
| 1895 | 9.25E-003 | 1clkA | VPMATTNLFTHPVFKD |
| 1896 | 9.25E-003 | 1clkA | WGGREG |
| 1897 | 9.63E-003 | 11baA | RQHMD |
| 1898 | 9.63E-003 | 11baA | CKNGQTNC |
| 1899 | 9.63E-003 | 11baA | CKPVNTF |
| 1900 | 9.63E-003 | 11baA | KFERQH |
| 1901 | 9.63E-003 | 11baA | RQHMDS |
| 1902 | 9.63E-003 | 11baA | SKYPNC |
| 1903 | 9.63E-003 | 11baA | VPVHFDA |
| 1904 | 9.63E-003 | 11baA | YPNCAY |
| 1905 | 1.00E-002 | 1ek5A | TGVRDY |
| 1906 | 1.00E-002 | 1ek5A | FSSSATVYG |
| 1907 | 1.00E-002 | 1ek5A | GGAGYIGSH |
| 1908 | 1.00E-002 | 1ek5A | GIPNNL |
| 1909 | 1.00E-002 | 1ek5A | LLRYFNP |
| 1910 | 1.00E-002 | 1ek5A | VIHFAGLK |
